# Stepwise neofunctionalization of the NF-κB family member Rel during vertebrate evolution

**DOI:** 10.1038/s41590-025-02138-2

**Published:** 2025-04-30

**Authors:** Allison E. Daly, Abraham B. Chang, Prabhat K. Purbey, Kevin J. Williams, Shuxing Li, Benjamin D. Redelings, George Yeh, Yongqing Wu, Scott D. Pope, Byrappa Venkatesh, Sibon Li, Kaylin Nguyen, Joseph Rodrigues, Kelsey Jorgensen, Ananya Dasgupta, Trevor Siggers, Lin Chen, Stephen T. Smale

**Affiliations:** 1 Department of Microbiology, Immunology, and Molecular Genetics, UCLA, Los Angeles, CA USA; 2 Molecular Biology Institute, UCLA, Los Angeles, CA USA; 3 Department of Medicine, UCLA, Los Angeles, CA USA; 4 Department of Biological Sciences, University of Southern California, Los Angeles, CA USA; 5 Department of Ecology and Evolutionary Biology, University of Kansas, Lawrence, KS USA; 6 Comparative Genomics Lab, Institute of Molecular and Cell Biology, Agency for Science, Technology, and Research, Singapore, Singapore; 7 Department of Human Genetics, UCLA, Los Angeles, CA USA; 8 Department of Anthropology, University of Kansas, Lawrence, KS USA; 9 Howard Hughes Medical Institute, Chevy Chase, MD USA; 10 Department of Biology, Boston University, Boston, MA USA

**Keywords:** Gene regulation in immune cells, Innate immune cells

## Abstract

Adaptive immunity and the five vertebrate NF-κB family members first emerged in cartilaginous fish, suggesting that NF-κB family divergence helped to facilitate adaptive immunity. One specialized function of the NF-κB Rel protein in macrophages is activation of *Il12b*, which encodes a key regulator of T cell development. We found that *Il12b* exhibits much greater Rel dependence than inducible innate immunity genes in macrophages, with the unique function of Rel dimers depending on a heightened intrinsic DNA-binding affinity. Chromatin immunoprecipitation followed by sequencing experiments defined differential DNA-binding preferences of NF-κB family members genome-wide, and X-ray crystallography revealed a key residue that supports the heightened DNA-binding affinity of Rel dimers. Unexpectedly, this residue, the heightened affinity of Rel dimers, and the portion of the *Il12b* promoter bound by Rel dimers were largely restricted to mammals. Our findings reveal major structural transitions in an NF-κB family member and one of its key target promoters at a late stage of vertebrate evolution that apparently contributed to immunoregulatory rewiring in mammalian species.

## Abstract (web summary)

Smale and colleagues explore evolutionary divergence in the NF-κB family. They show that intrinsic DNA-binding affinity rather than specificity led to neofunctionalization in mammals within this transcription factor family, supporting immunoregulatory rewiring in mammalian species.

## Main

The V(D)J adaptive immune system, which is found in almost all jawed vertebrates (Gnathostomata), relies on interactions among hundreds of cell types^1–4^. Although this system is fundamentally similar among gnathostomes, numerous differences between species have been described^4^. These differences include the absence of immunoglobulins in some teleost fish and the absence of γδ T cells in squamate reptiles^5,6^. Biological reasons for some of these differences have been proposed. For example, in some deep-sea anglerfish species, males permanently attach to host females in a form of sexual parasitism^7^. To prevent immune incompatibility between the attached partners, anglerfish genomes have lost several genes that contribute to adaptive immunity^7^. Other differences between species are likely to reflect the rewiring of immunoregulatory pathways. For example, a placental mammal-specific enhancer has been identified in the gene encoding FoxP3, which controls regulatory T cell development^8^. This new regulatory strategy may have evolved to support mammalian fetal tolerance^8^.

Gene duplication helps to drive evolution through multiple mechanisms^9,10^. Subfunctionalization occurs when the functions of an ancestral protein are divided among its descendants. Neofunctionalization represents the emergence of new functions in descendants, through either mutations that generate new protein functions or noncoding mutations that alter the expression patterns of duplicated genes. Evolutionary trajectories are influenced by the epistatic effects of mutations on protein structure, and also on the interactions of proteins with other molecules^11,12^. For transcription factors, evolutionary trajectories must accommodate epistasis between the factor and its genomic binding sites^11,12^.

The vertebrate NF-κB Rel transcription factor family comprises five members that have prominent roles in innate and adaptive immunity^13–15^. NF-κB proteins are characterized by a Rel homology region (RHR) that supports DNA binding and assembly of homodimers and heterodimers^14^. Most dimers are retained in the cytoplasm in association with IκB inhibitor proteins, with IκB degradation directed by signaling pathways upon sensing of microbial and environmental threats. Loss-of-function studies have revealed the biological functions of each family member^14,16^. However, much less is known about the regulatory mechanisms of each dimeric species.

RelA and Rel are the most closely related NF-κB family members. RelA is broadly expressed, whereas high Rel expression occurs primarily in hematopoietic cells^14,16^. Mice deficient in *Rela* (encoding RelA) exhibit embryonic lethality, whereas mice deficient in *Rel* exhibit various immune abnormalities, including diminished T cell and B cell responses, defective regulatory T cell development, and loss of T helper 1 (T_H_1) and T helper 17 (T_H_17) cells^14,17,18^. Although Rel and RelA often act redundantly^14,19^, Rel is required for induction of several genes^17^. We and others have described a potent role for Rel in *Il12b* activation in mouse and human macrophages and some dendritic cell subsets^20–23^. Regulation of *Il12b* by Rel, which extends to patients with inherited human *Rel* deficiency^23^, helps to explain the importance of Rel in T_H_1 and T_H_17 responses, as *Il12b* encodes a common subunit of the IL-12 and IL-23 cytokines needed for T_H_1 and T_H_17 cell development, respectively^24^.

In a chimeric protein analysis, the Rel requirement for *Il12b* induction was localized to a portion of the RHR containing 46 amino acid differences between RelA and Rel^25^. Despite identical DNA-contacting residues and consensus recognition motifs for RelA and Rel^26–28^, these residues enable Rel homodimers to bind to NF-κB motifs with a much higher intrinsic affinity than RelA homodimers^25,28^. Thus, Rel-dependent *Il12b* induction does not reflect a distinct motif preference but rather a heightened intrinsic DNA-binding affinity of Rel homodimers that is important for functional interactions at a subset of NF-κB sites. We have proposed that this heightened binding affinity may be especially important at nonconsensus motifs at which RelA binding may be too weak to support transcriptional induction^25,28^.

Here, we showed that in activated macrophages, the strong dependence of *Il12b* on Rel is extremely rare among inducible genes. Moreover, Rel homodimers bound to motifs that bore little resemblance to canonical NF-κB motifs, owing to their heightened intrinsic DNA-binding affinity. Rel-dependent transcription appears to involve preferential Rel binding to nonconsensus motifs combined with a promoter architecture conducive to selective activation. Notably, the difference in affinity between Rel and RelA was not observed in orthologs from most nonmammalian vertebrates. Structural analyses identified a mammal-specific Rel residue that makes a major contribution to the difference in affinity. In addition, a key segment of the *Il12b* promoter recognized by Rel homodimers was found only in mammalian species. Together, these results reveal major structural transitions within an NF-κB family member and the promoter of one of its critical target genes during vertebrate evolution, presumably for the purpose of rewiring adaptive immune regulation in mammalian species.

## Results

### Evolution of the NF-κB Rel family

**Fig. 1:**
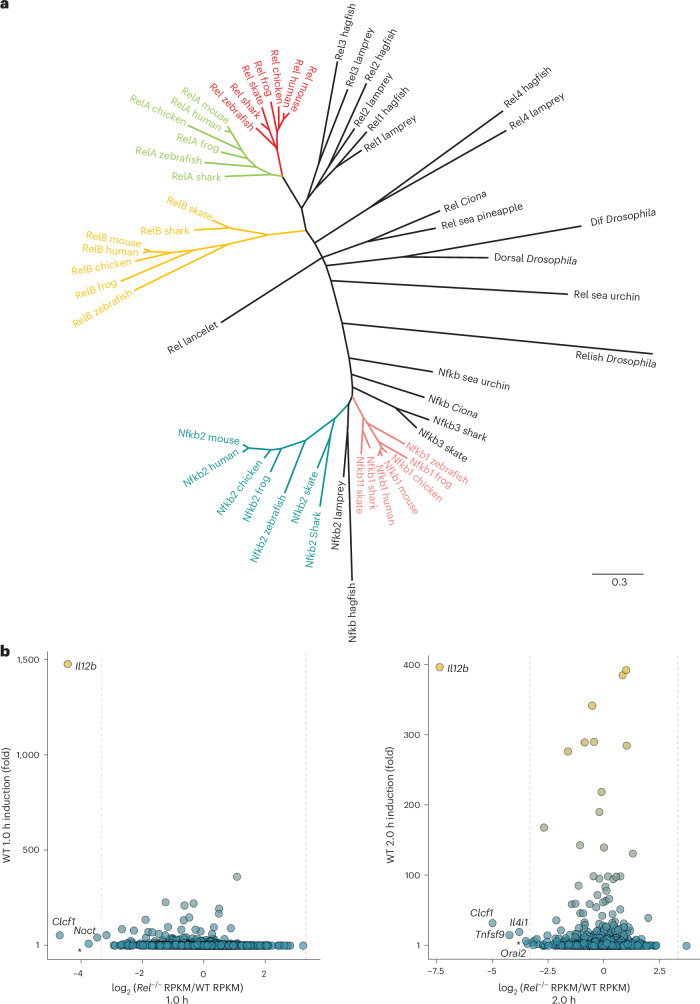
NF-κB phylogenetic analysis and highly selective Rel requirement. **a**, An unrooted phylogenetic tree was prepared with the RHR amino acid sequences of NF-κB family members from representative invertebrate and vertebrate species. All sequences were from public databases (Supplementary Tables 1 and 2). No thorny shark ortholog of RelA could be identified. Clusters of the five vertebrate family members are colored. **b**, Scatter plots comparing nascent transcript RNA-seq datasets from lipid-A-expressed genes (RPKM > 3; *n* = 2,980 genes) in WT versus *Rel*^−/−^ C56BL/6 macrophages. The plots show the log_2_ *Rel*^−/−^ RPKM/WT RPKM ratios (*x* axis) and the fold induction values at the indicated time point (*y* axis), with all values representing averages of two independent biological replicates of the time course (the WT and mutant samples were examined in parallel). Plots are shown for the 1 h and 2 h stimulation time points. Genes exhibiting the strongest *Rel* dependence (fold change >10) are labeled. Vertical dashed lines denote ten-fold difference. All dependent genes were induced at least 15-fold, except *Orai2* (2.9-fold). One additional gene that was not reproducibly dependent on *Rel* (*Mir6903*) is labeled with an asterisk.

**Extended Data Fig. 1:**
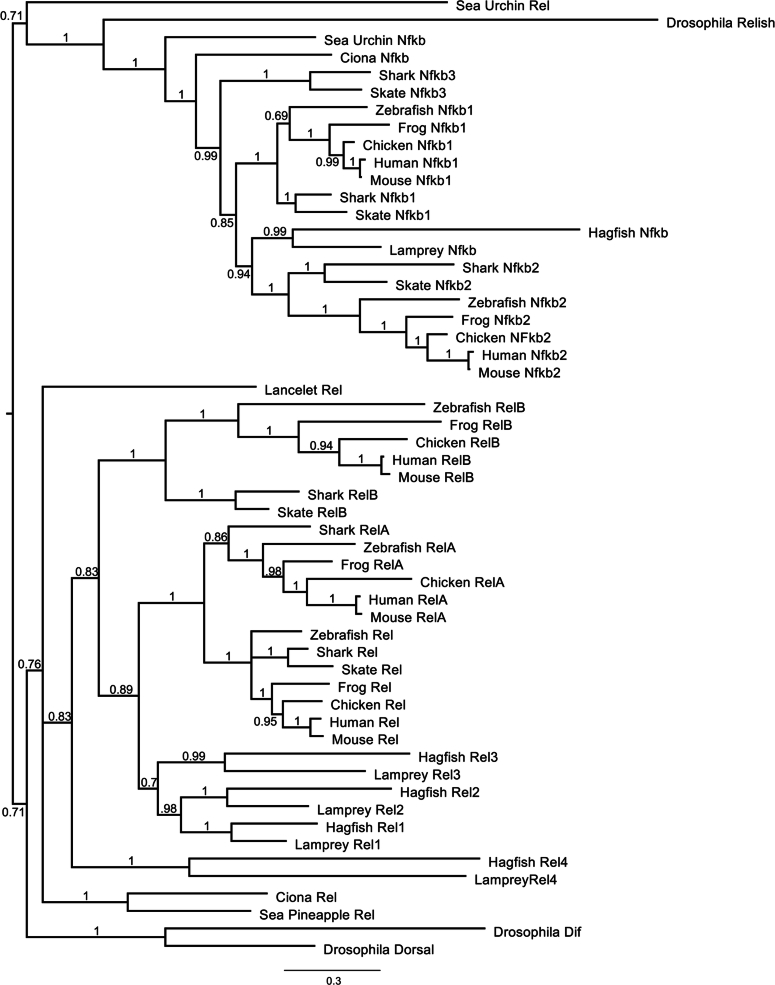
Rooted phylogenetic tree of NF-κB family members. A rooted phylogenetic tree prepared with the same sequences used to generate the unrooted tree in Fig. 1a is shown. For the Bayesian phylogeny estimate, branches are labeled with posterior probability to indicate the degree of support. The figure shows the 66% consensus tree, which shows every branch with support >0.66. The scale at the bottom provides an absolute scale for the amount of evolutionary change between any two tips (average number of amino acid changes per site).

To better understand the evolution of the NF-κB Rel family, we prepared phylogenetic trees from RHR sequences (Fig. 1a, Extended Data Fig. 1 and Supplementary Tables 1 and 2). The RHRs of each family member—Nfkb1/p50 (*Nfkb1*), Nfkb2/p52 (*Nfkb2*), RelA (*Rela*), Rel (*Rel*) and RelB (*Relb*)—were conserved in all gnathostome species examined. Notably, the shark and skate genomes, representative of the most primitive gnathostome class (Chondrichthyes), encoded an additional NF-κB protein related to their Nfkb1 and Nfkb2 paralogs (Fig. 1a; Nfkb3; note that no thorny shark RelA ortholog could be identified).

Invertebrate NF-κBs diverged considerably from the vertebrate clusters (Fig. 1a). It was therefore of interest to examine NF-κBs in *Petromyzon marinus* (sea lamprey) and *Eptatretus atami* (hagfish), which are species of cyclostomes (jawless fish) that diverged from gnathostomes 450 million years ago. By mining genomes of these species, we identified five Rel NF-κB genes in each, including three previously described in lamprey^29^. All five family members were aligned more closely among these species than with the other vertebrate species (Fig. 1a). Notably, none of the lamprey or hagfish NF-κBs clustered closely with RelA, Rel or RelB, suggesting that the distinguishing features of these proteins appeared after the cyclostomata–gnathostomata divergence.

**Extended Data Fig. 2:**
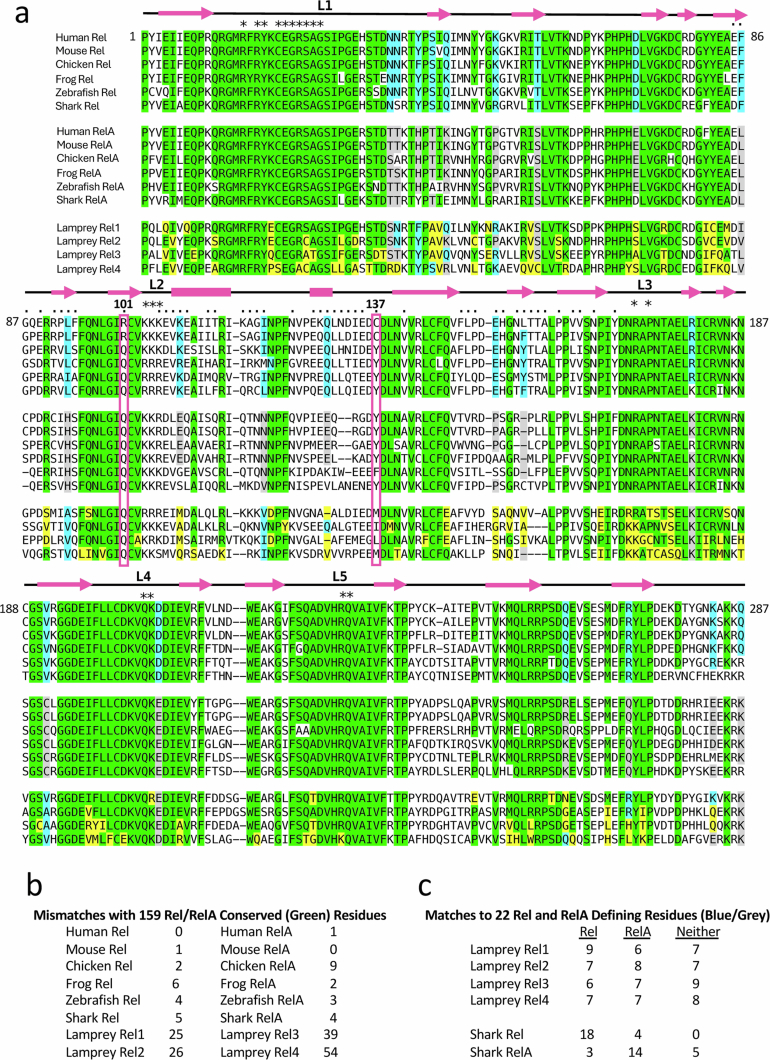
Comparison of RHR sequences. **a**, RHR sequences are shown for Rel and RelA orthologs from six gnathostome species, plus four sea lamprey Rel proteins. The fifth sea lamprey RHR is not shown due to its homology to the NF-κB p50 and p52 RHRs. Amino acids are shaded in green that are perfectly conversed in at least five of six species for Rel and in at least five of six species for RelA. These residues are also in green in the lamprey RHRs if they match the gnathostome residue, and in yellow if they do not match. 22 ‘defining’ residues that most consistently distinguish the Rel and RelA RHRs are highlighted in blue (Rel) and grey (RelA). These residues are sometimes identical in all six gnathostome Rel or RelA RHRs, or they may be members of the same amino acid class. These residues are shaded in blue or grey in the lamprey RHR sequences if they match the characteristics of the Rel or RelA defining residues, respectively, and they are shaded in yellow if they do not match either the Rel or RelA characteristics. Structural features are indicated, including the locations of α helices (bars), β-strands (arrows), and the L1-L5 loops. DNA-contacting residues are depicted with asterisks and the 46 residues shown in a previous study to be responsible for the DNA-binding affinity differences^25^ between Rel homodimers and RelA homodimers are shown as dots. RHR positions 101 and 137, examined in Fig. 7a, b, are highlighted with a red box. **b**, For the 159 residues shaded in green in panel A, the number of mismatches in each of the sixteen RHRs is shown. Note that all four of the lamprey RHRs contain more mismatches than any of the RHRs in the six gnathostome species. **c**, For the 22 defining residues that consistently distinguish the Rel RHRs from the RelA RHRs, the numbers of residues in each lamprey RHR that match the Rel residue, match the RelA residue, or do not match either residue are indicated. Matches are also shown for elephant shark Rel and RelA.

Closer analysis provided further evidence that the lamprey and hagfish RHRs lacked the characteristic features of RelA and Rel. All four lamprey Rel RHRs contain similar numbers of mismatches to the 159 residues that are most highly conserved between Rel and RelA in other vertebrates (Extended Data Fig. 2a,b). In addition, in an analysis of 22 residues that most consistently distinguished Rel and RelA, none of the lamprey proteins was strongly biased toward either Rel or RelA (Extended Data Fig. 2c). Owing to genome tetraploidization events that occurred before and after cyclostome–gnathostome divergence^30^, the ancestral relationship between the lamprey and hagfish Rel proteins and the RelA and Rel proteins is difficult to predict. However, the fact that the Rel and RelA RHRs exhibit greater similarity to each other than to any of the lamprey or hagfish RHRs suggests that Rel and RelA arose by duplication of a single gnathostome ancestral gene. Moreover, because Rel and RelA, like V(D)J adaptive immunity, are characteristic of gnathostomes, their divergence may have helped to support the emergence of adaptive immunity.

### Highly selective roles of Rel in lipid-A-stimulated bone-marrow-derived macrophages

Despite redundancy in some settings, Rel and RelA contribute unique functions to the regulation of adaptive immunity^14,16,22,31^. To improve our understanding of the functions of Rel in an innate immune cell type, we performed RNA sequencing (RNA-seq) with wild-type (WT) and *Rel*^−/−^ bone-marrow-derived macrophages (BMDMs) stimulated with lipid A for 0, 60 and 120 min. For this analysis, we used nascent transcript RNA-seq (Fig. 1b) to enable quantitative analysis of the impact of Rel on transcription. Surprisingly, strong Rel-dependent expression was observed for only a small number of genes, including *Il12b*, *Clcf1* (BSF-3), *Tnfsf9* (4-1BB-L), *Noct*, *Il4i1* and *Orai2* (Fig. 1b). Moreover, among the Rel-dependent genes, *Il12b* was the most potently induced by lipid A (Fig. 1b; *Il12b* was induced 1,475-fold at the 60-min time point, with the other genes induced no more than 54-fold at either time point).

A primary function of the protein encoded by *Il12b* is to regulate T_H_1 and T_H_17 adaptive immunity. Moreover, regulation of *Il12b* expression by Rel has been linked to regulation of T_H_1 and T_H_17 cell development^23,32,33^. The functions of the other genes exhibiting strong Rel dependence in innate immunity are poorly understood, but roles for a subset of these genes in the regulation of adaptive immune responses have been described^34–36^. Thus, the RNA-seq results are consistent with the hypothesis that during vertebrate evolution, neofunctionalization of Rel enabled it to support adaptive immunity, via its regulation of *Il12b* and other macrophage-expressed genes that regulate adaptive immunity, combined with its numerous functions in T and B cells.

### Rel homodimer binding to highly divergent motifs

**Fig. 2:**
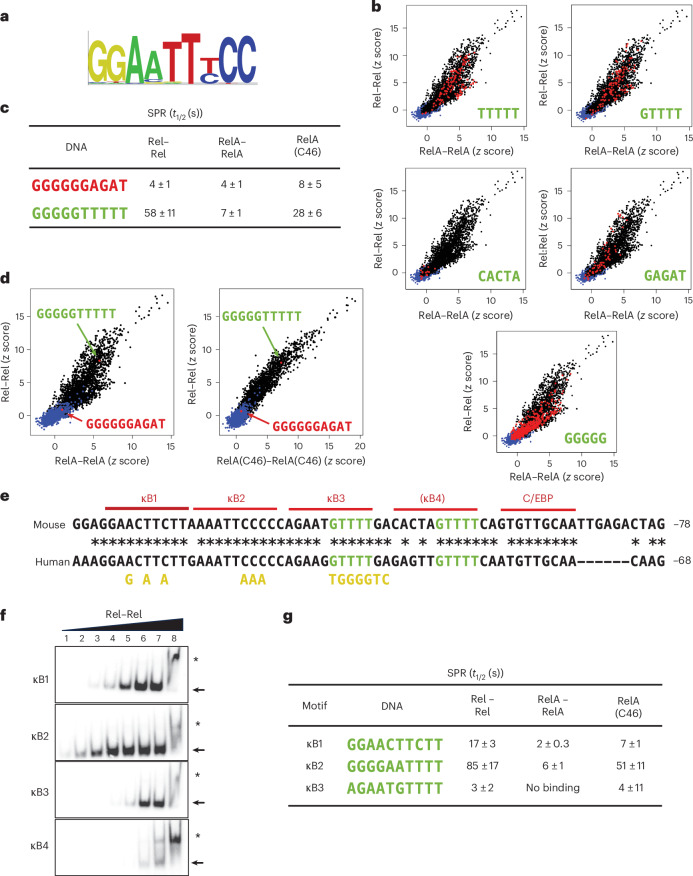
PBM, SPR and EMSA evidence of NF-κB binding to a novel DNA motif. **a**, A consensus motif for RelA and Rel homodimers is shown^28^. **b**, PBM scatter plots comparing binding (*z* score) of RelA (*x* axis) and Rel (*y* axis) homodimers to a range of oligonucleotides. Oligonucleotides containing the sequence shown in each graph are highlighted (red), revealing strong binding (*z* > 4) to many oligonucleotides containing TTTTT or GTTTT. Strong binding was observed for smaller percentages of oligonucleotides containing CACTA, GAGAT or GGGGG. The diagonal shows that RelA and Rel had similar DNA preferences. However, determination of their relative affinities for a motif requires use of SPR. **c**, SPR results showing that Rel homodimers bound to GGGGGTTTTT with a slower off-rate than GGGGGGAGAT. Mean values with standard deviations are shown from three independent experiments. The RelA(C46) chimera exhibited a slower off-rate than RelA homodimers, confirming the importance of the 46 Rel residues. **d**, The locations of the oligonucleotides from **c** are shown on PBM profiles. **e**, Partial mouse and human *Il12b* promoter sequences are shown, with conserved nucleotides indicated (asterisks). The previously described Rel homodimer binding sites (κB1 and κB2) and a binding site for C/EBP proteins are indicated. Two additional potential NF-κB binding motifs (κB3 and κB4) are also indicated. GTTTT sequences are highlighted (green). The κB4 motif is shown in parentheses because of its uncertain functional relevance. Sequences in yellow correspond to mutations used for the functional experiment shown in Fig. 3c. **f**, EMSAs were performed with probes containing each potential NF-κB motif from the mouse *Il12b* promoter. Increasing concentrations of recombinant Rel RHR homodimer were used. An image representative of two independent experiments is shown. The locations of the predicted homodimer–DNA complexes are indicated (arrowheads), as well as complexes that may represent aggregates (asterisks). **g**, SPR was used to determine off-rates of Rel, RelA and RelA(C46) RHR homodimers with the *Il12b* κB1, κB2 and κB3 motifs (note that the κB2 motif sequence is the opposite strand of the motif in **e**). No reproducible binding was observed with the κB4 sequence. Mean values and standard deviations from three independent experiments for each protein and DNA probe are shown.

A study using protein-binding microarrays (PBMs) previously revealed that Rel and RelA homodimers bind to similar DNA-recognition motifs^28^, resulting in the same consensus sequence (Fig. 2a)^28^. However, surface plasmon resonance (SPR) experiments showed that Rel homodimers bound with a much higher affinity than RelA homodimers to the full range of sequences evaluated by PBM^28^. That is, the PBM results showed the relative strength of binding of a given protein to the motifs present on the array, but SPR compared the relative affinities of Rel homodimers versus RelA homodimers.

Although most sequences displaying high *z* scores for Rel and RelA homodimer binding exhibited similarity to consensus NF-κB motifs^28^, scrutiny of the previously determined PBM profiles revealed that oligonucleotides containing either four or five tandem T:A base pairs also frequently exhibited high *z* scores. As examples, PBM profiles are shown for oligonucleotides containing either TTTTT or GTTTT in Fig. 2b (top). High *z* scores were much less frequent for oligonucleotides containing many other sequences (for example, Fig. 2b, middle and bottom).

Binding to oligonucleotides with tandem T:A base pairs was unexpected, because NF-κB binding energy is primary due to contacts with G:C base pairs at the recognition sequence flanks (Fig. 2a)^26^. However, SPR results revealed that five T:A bps in one motif half-site resulted in a much greater Rel homodimer half-life than that observed with a different half-site sequence (Fig. 2c; see Fig. 2d for locations of oligonucleotides examined within the PBM profiles). RelA homodimer binding to both oligonucleotides was much weaker (Fig. 2c). The Rel–RelA binding difference was largely due to the 46 Rel residues responsible for the high DNA-binding affinity of Rel^25,28^, as binding of a RelA(C46) chimeric protein (containing 46 Rel residues inserted into RelA) to the motif with tandem T:A bps exhibited a half-life that was only moderately reduced in comparison with that of WT Rel (Fig. 2c).

### A novel, unrecognizable NF-κB motif in the *Il12b* promoter

Unexpectedly, the mouse and human *Il12b* promoter sequences contained two conserved sequences with four tandem T:A base pairs each (Fig. 2e, κB3 and κB4) immediately downstream of two previously described nonconsensus NF-κB motifs (Fig. 2e, *Il12b* κB1 and κB2)^25,37^. The nonconsensus *Il12b* κB1 and κB2 motifs (but not the *Il12b* κB3 or κB4 motifs) contained the two or three tandem G:C base pairs in one half-site that support stable binding. Although κB3 and κB4 are unrecognizable as NF-κB motifs (owing to the absence of tandem G:C bps), we considered the possibility that they may contribute to *Il12b* activation.

**Fig. 3:**
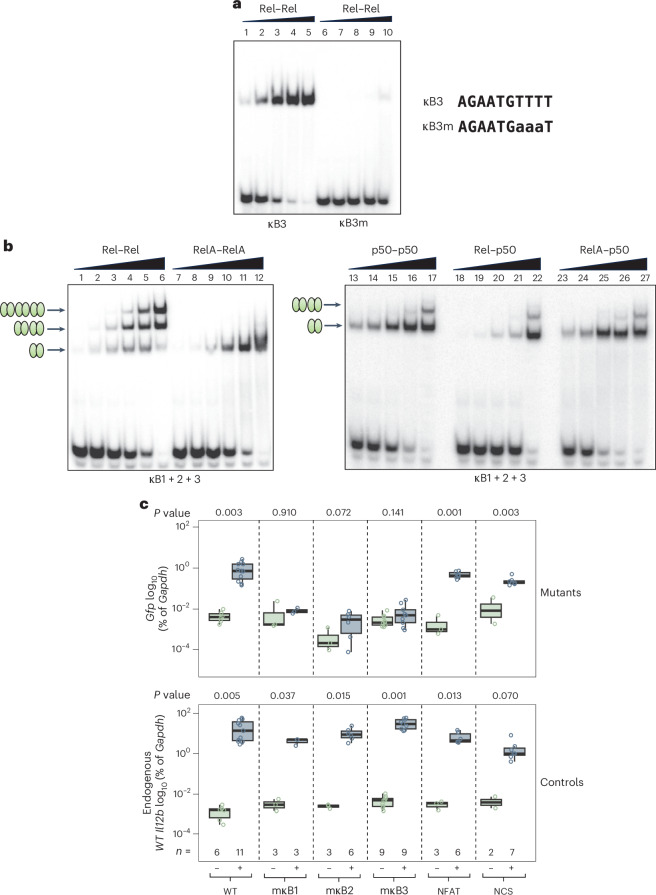
Simultaneous Rel homodimer-specific binding to the tandem *Il12b* promoter motifs and functional roles of the motifs. **a**, EMSA was used to examine binding of increasing concentrations of the recombinant Rel RHR protein to probes containing the WT κB3 motif or a mutant motif (κB3M). An image representative of two independent experiments is shown. **b**, EMSAs were used to examine binding of purified recombinant Rel–Rel, RelA–RelA, p50–p50, Rel–p50 and RelA–p50 RHR dimers to a probe containing *Il12b* promoter sequences spanning the κB1, κB2 and κB3 motifs. The locations of complexes containing one, two and three bound dimers are indicated. The unbound probe is visible at the bottom. Images representative of three independent experiments are shown **c**, A 200-kb BAC encompassing the mouse *Il12b* locus was engineered in *E. coli* to contain *Gfp*-expressing sequences and further engineered with mutations in the *Il12b* κB1, κB2 or κB3 sequence (Extended Data Fig. 4b; mutations shown in Fig. 2e), with mutations introduced into an NFAT recognition sequence and a nonconserved sequence (NCS) in the promoter as controls. The recombinant BACs were then stably integrated into mouse ES cells, and 2–6 independent clones containing single-copy BAC integrants were selected and analyzed for each mutant. Following differentiation of the ES cells into terminally differentiated macrophages, cells were stimulated with lipopolysaccharide for 0 or 2 h, followed by mRNA isolation. qRT–PCR was then used to quantify relative levels in each clone of the endogenous *Il12b* mRNA (as a control to confirm cell activation) and the BAC *Il12b*–*Gfp* mRNA (detected using *Gfp* primers). Data (percentage expression of *Gfp* or endogenous *Il12b* mRNA relative to *Gapdh*) for WT and mutant clones are shown as box plots, with *P* values between unstimulated (−) and lipopolysaccharide-stimulated (+) samples calculated using unpaired Welch’s *t*-test. The numbers of replicates (*n*) examined for each WT or mutant construct are indicated. The box limits represent the interquartile range (25th to 75th percentile), and the central line in each box shows the median value. The whiskers extend to the most extreme data points with 1.5 times the interquartile range. Individual points outside the whiskers represent outliers.

We first performed electrophoretic mobility shift assay (EMSA) experiments with the Rel RHR. Rel homodimers bound to each of the four *Il12b* promoter motifs, albeit with highly variable affinities (Fig. 2f). SPR confirmed that Rel homodimers bound most stably to the κB2 motif, followed by the κB1 and κB3 motifs (Fig. 2g). Binding to κB4 was not detected by SPR, consistent with the very weak binding observed with EMSA (Fig. 2f). As κB4 binding was barely detectable, we did not further study this motif (and we have shown it in parentheses in Fig. 2e). Notably, introduction of mutations in the GTTTT half-site of κB3 confirmed that this half-site is critical for Rel binding (Fig. 3a).

**Extended Data Fig. 3:**
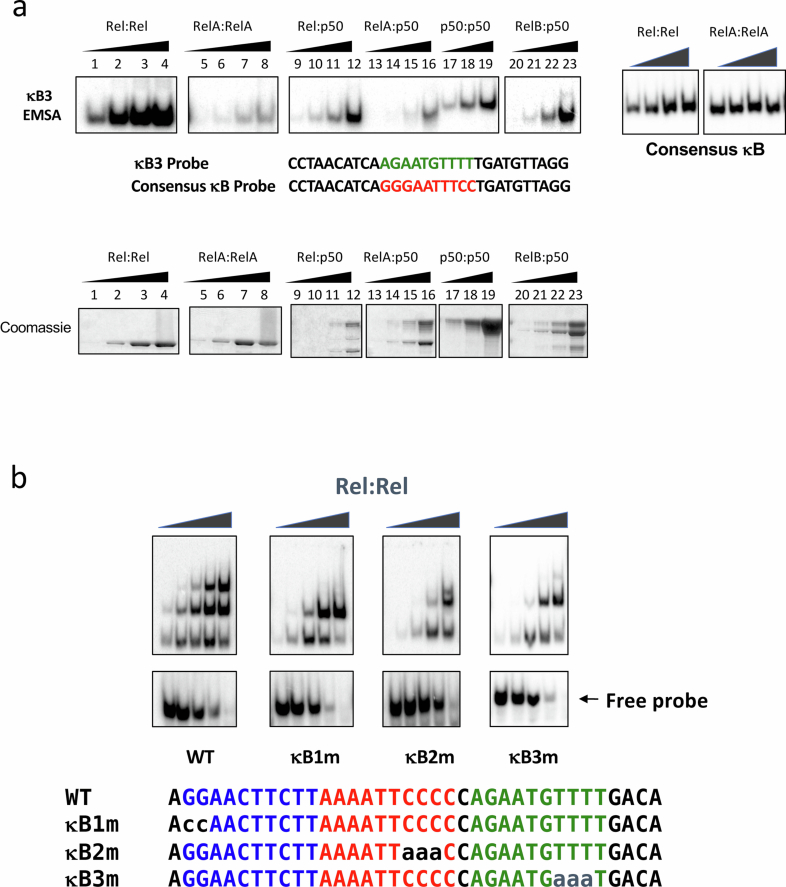
Binding of NF-κB dimers to *Il12b* motifs. **a**, EMSAs were performed to examine the relative binding strength of various NF-κB RHR homodimers and heterodimers to a radiolabeled probe containing the *Il12b* κB3 sequence. For heterodimers, two NF-κB family members were co-expressed in E. coli. Coomassie stains of the purified are shown at the bottom. Binding of Rel and RelA homodimers to a probe containing a consensus NF-κB motif is shown at the right as a control. Representative images from two or three independent experiments are shown **b**, EMSAs were used to examine Rel RHR binding to probes containing substitution mutations in the *Il12b* κB1, 2, and 3 motifs in a radiolabeled probe containing these three motifs in tandem, as in the WT *Il12b* promoter. The images are representative of two independent experiments.

Despite the readily detected binding of Rel homodimers to κB3, RelA binding could not be detected by SPR (Fig. 2g). Nevertheless, the half-life of the chimeric RelA(C46) protein was only moderately reduced compared with that of Rel (Fig. 2g). Binding to κB3 by Rel homodimers was stronger than the binding observed with six other homodimeric and heterodimeric NF-κB species (Extended Data Fig. 3a).

We next examined binding to a probe containing the *Il12b* κB1, κB2 and κB3 sequences and observed that Rel homodimers could simultaneously bind to all three motifs (Fig. 3b). Mutation of any of the three motifs eliminated the slowest mobility band (Extended Data Fig. 3b). By contrast, only one RelA homodimer could bind to this oligonucleotide (Fig. 3b). Other dimeric species were also incapable of simultaneous binding (Fig. 3b, right). Together, these results suggest that preferential binding of Rel homodimers to three tandem *Il12b* promoter motifs in the *Il12b* promoter may underlie the Rel dependence of *Il12b* transcription.

### Functional significance of the κB3 motif

**Extended Data Fig. 4:**
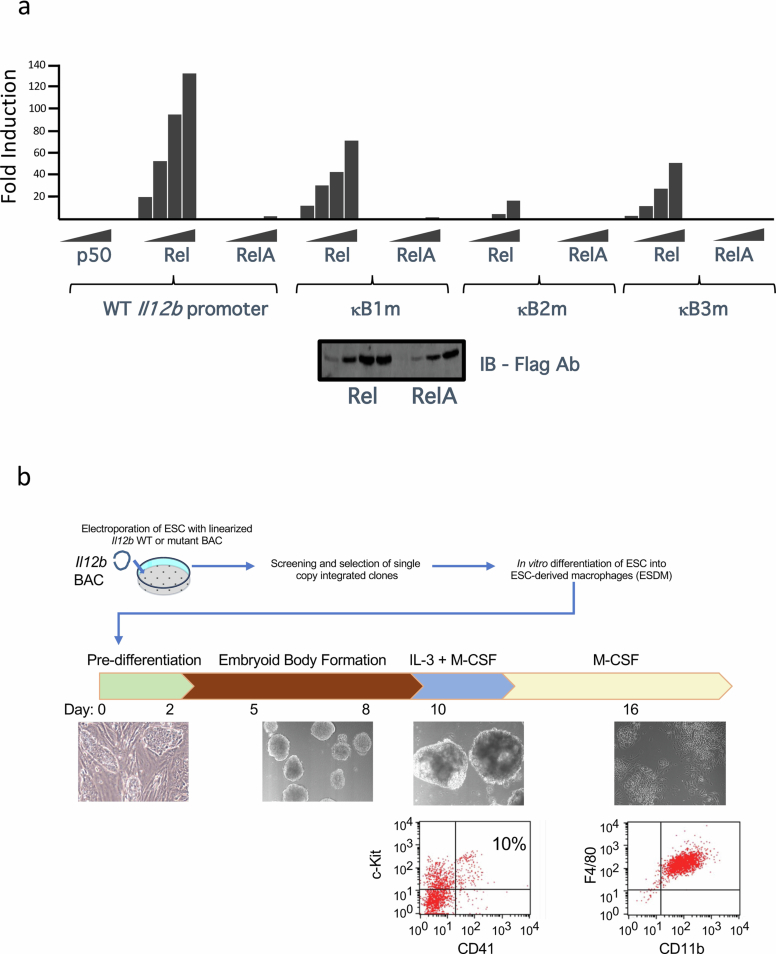
Functional analysis of non-consensus NF-κB motifs. **a**, HEK293T cells were transiently transfected with a mouse *Il12b* promoter-luciferase reporter plasmid and expression plasmids for full-length Flag epitope-tagged RelA, Rel, and p50. Luciferase activity was monitored with plasmids containing the WT *Il12b* promoter and plasmids containing substitution mutations in the *Il12b* κB1, κB2, and κB3 motifs. Titration results from two independent experiments are shown. As previously observed^37^, Rel overexpression activated *Il12b* promoter activity more strongly than RelA overexpression. Promoter activity was compromised to variable extents by mutation of each of the three motifs. **b**, The strategy used to analyze substitution mutations in the mouse *Il12b* promoter in a more native chromatin context is shown (performed prior to the emergence of CRISPR-Cas9 in mammalian cells). A bacterial artificial chromosome (BAC) spanning the mouse *Il12b* locus was modified by homologous recombination in E. coli to add a *Gfp* cDNA into the *Il12b* transcription unit. This *Il12b-Gfp* BAC was then further modified by homologous recombination in E. coli to introduce mutations into five locations in the *Il12b* promoter: the κB1, κB2, κB3, and NFAT motifs, and a fifth sequence that is not conserved between species. The recombinant BACs were introduced into mouse ESC by electroporation and multiple independent clones for each BAC were isolated and expanded. Following the identification of clones containing single-copy BAC integrants, those clones were expanded and differentiated in vitro into post-mitotic macrophages. The ESC-derived macrophages were then stimulated for 2 h with LPS. Flow cytometry was used to monitor CD41 and c-kit expression to confirm partial differentiation of ESC into macrophage progenitors. CD11b and F4/80 were then monitored by flow cytometry, demonstrating efficient differentiation into macrophages. The microscopic and flow cytometry histogram images that are shown were captured during one of the differentiation experiments.

To examine the functional significance of κB3, we first tested *Il12b*-promoter–luciferase reporter plasmids. As previously shown^37^, Rel overexpression in HEK 293T cells activated the WT *Il12b* promoter much more strongly than RelA (Extended Data Fig. 4a). Mutations in any of the three NF-κB motifs reduced transactivation (Extended Data Fig. 4a).

To examine the motifs in a native chromosomal context, we developed a bacterial artificial chromosome (BAC) strategy before the emergence of CRISPR–Cas9 methodology (Extended Data Fig. 4b). Using a 200-kb *Il12b* BAC, into which we incorporated an enhanced green fluorescent protein complementary DNA (*Egfp* cDNA) to monitor BAC-derived *Il12b* expression, we introduced substitution mutations into the κB1, κB2 and κB3 motifs (mutant sequences are shown in Fig. 2e in yellow). Two additional mutations (NFAT and nonconserved sequence) were examined as controls. The BACs were then stably transfected into a ES cell line (Extended Data Fig. 4b). After selection of multiple clones for each BAC containing single-copy integrants, the clones were differentiated into macrophages, followed by lipopolysaccharide stimulation (Extended Data Fig. 4b). Quantitative PCR with reverse transcription (qRT–PCR) was used to monitor induction of the *Il12b*–*Gfp* transcription (using *Gfp* primers; Fig. 3c, top). Endogenous *Il12b* transcription (using *Il12b* primers) was examined by qRT–PCR as a control (Fig. 3c, bottom). With this strategy, the WT *Il12b*–*Gfp* gene was activated, on average, >100-fold (Fig. 3c, top). By contrast, *Il12b*–*Gfp* activation was greatly reduced in clones containing mutations in the κB1, κB2 or κB3 motif (Fig. 3c, top). These findings confirm functional roles for all three κB motifs.

### Rel- versus RelA-preferential binding in vivo

**Fig. 4:**
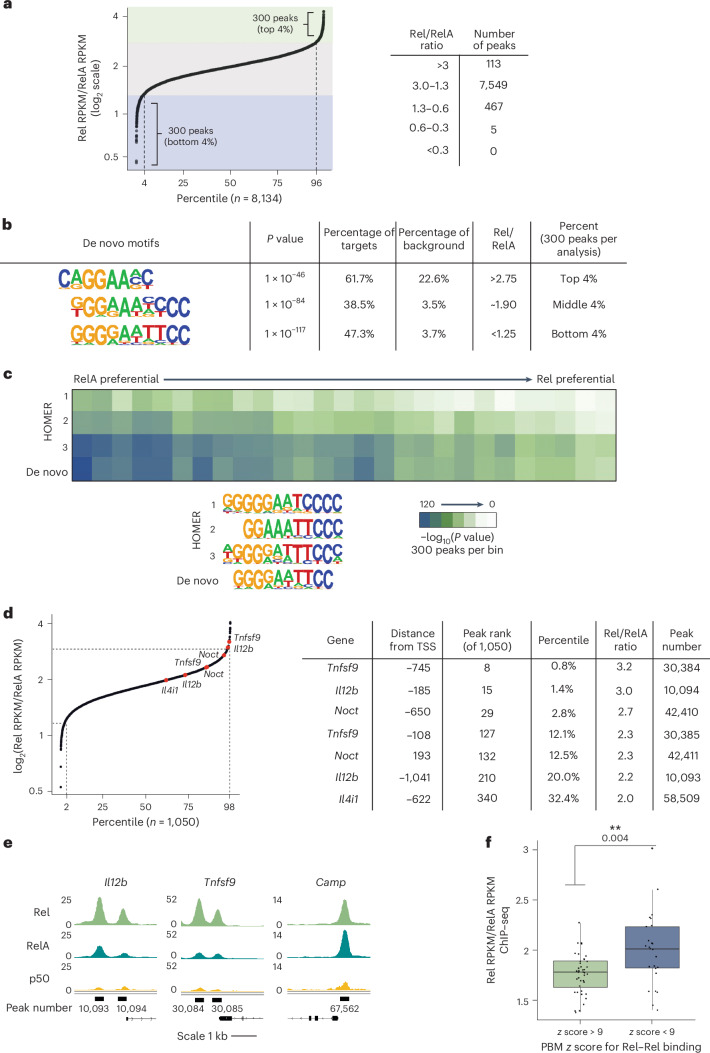
Selective binding of Rel and RelA in mouse BMDMs examined by ChIP–seq. **a**, ChIP–seq was performed with RelA and Rel antibodies in lipid-A-stimulated (1 h) BMDMs (five and three biological replicates, respectively). The 6,700 strongest peaks for each antibody were merged, yielding 8,134 peaks. Rel/RelA RPKM ratios are plotted on the *y* axis, with the 8,134 peaks along the *x* axis (displayed as the percentage of total peaks). On the right, numbers of peaks with different ranges of RPKM ratios are shown. **b**, The most enriched motifs from de novo motif analyses performed with 300 peaks representing the largest or smallest ratios and 300 peaks with intermediate ratios. **c**, The 8,134 peaks were divided into 27 equal bins based on Rel/RelA RPKM ratios. The enrichment of three consensus NF-κB motifs within HOMER, and the consensus NF-κB motif from **b**, was then examined. **d**, Among the 8,134 peaks, 1,050 were annotated to promoter regions (−1,500 bp to +500 bp relative to the transcription start site). The Rel/RelA RPKM ratios of these peaks were plotted and used to rank the peaks in the promoter regions of Rel-dependent genes (Fig. 1b). The table lists the Rel-dependent genes, the peak distance from the transcription start site, the RPKM rank of each peak, the peak rank percentile, the Rel/RelA RPKM ratio and the annotated peak number. **e**, IGV tracks are shown for two Rel-dependent genes (*Il12b* and *Tnfsf9)* and for one promoter peak with strong RelA binding in comparison with Rel (*Camp*). Tracks are for Rel, RelA and p50 from lipid-A-stimulated cells (1 h). **f**, Rel/RelA RPKM ratios calculated for 65 previously defined promoter peaks for lipid-A-inducible genes that coincided with recognizable NF-κB motifs (Supplementary Table 4). These peaks were separated into two groups based on PBM *z* scores for Rel homodimer binding: *z* > 9 (*n* = 40) and *z* < 9 (*n* = 25). The Rel/RelA RPKM ratios for each peak within each group are shown in the box plot. The lower and upper edges of the box represent the first and third quartiles. The upper whisker extends from the third quartile to the maxima within 1.5 times the interquartile range. The lower whisker extends from the first quartile to the minima within 1.5 times the interquartile range. Any values that fell beyond the whiskers were considered to be outliers. The statistical significance of the difference between the two groups (*P* value determined by Welch’s two sample *t*-test (two sided) is shown at the top.

To determine whether preferential Rel binding occurred in vivo, we performed chromatin immunoprecipitation followed by sequencing (ChIP–seq) for Rel and RelA in BMDMs stimulated with lipid A for 0 and 60 min (refs. ^38,39^). We focused on the strongest 6,700 peaks for each antibody, which together yielded 8,134 peaks. We then determined the reads per kilobase per million mapped reads (RPKM) ratio at each of the 8,134 peaks (Fig. 4a). This analysis revealed relatively strong preferences for Rel or RelA binding for small subsets of peaks but comparable binding for the vast majority. Specifically, at 93% of peaks, the Rel/RelA RPKM ratio was between 1.3 and 3 (Fig. 4a). (That the median ratio was 1.89 instead of 1.00 may reflect differences in antibody quality or broadly enhanced Rel interactions due to the heightened binding affinity of Rel.)

We next performed de novo motif analyses with the 300 peaks exhibiting the largest and smallest RPKM ratios. Peaks exhibiting the largest ratios showed the strongest enrichment of motifs resembling an NF-κB half-site (Fig. 4b). This suggests that the heightened affinity of Rel homodimers observed in vitro allows Rel to bind preferentially in vivo to many sites throughout the genome that diverge from consensus dimeric recognition motifs. RelA-preferential peaks exhibited enrichment of consensus motifs defined in vitro for RelA–p50 heterodimers^28^ (Fig. 4b). This finding could reflect a high abundance of RelA–p50 heterodimers in comparison with Rel–p50 heterodimers or noise in the analysis (see below). We also performed de novo motif analysis with 300 peaks from the middle of the Rel/RelA ratio spectrum and observed enrichment of a near-consensus dimeric NF-κB motif (Fig. 4b), which may represent interactions by a mixture of dimeric species.

We next examined the enrichment of four NF-κB consensus motifs across the Rel/RelA RPKM ratio spectrum (Fig. 4c). A trend toward weaker enrichment of all motifs with increasing Rel/RelA RPKM ratio was observed, consistent with the view that preferential Rel binding is most pronounced at divergent motifs. Most importantly, we graphed Rel/RelA RPKM ratios for 1,050 peaks that were annotated to promoter regions (Fig. 4d). Strongly preferential Rel binding was observed at two peaks within this region of *Il12b*, in addition to two promoter peaks for two other Rel-dependent genes, *Tnfsf9* and *Noct*. The *Il12b* peaks ranked 15th and 210th among the 1,050 peaks examined. The *Tnfsf9* peaks were ranked 8th and 127th, and the *Noct* peaks ranked 29th and 132nd (Fig. 4d). Integrative Genome Browser (IGV) tracks are shown for the *Il12b* and *Tnfsf9* promoters, and for the *Camp* promoter, which exhibited a low Rel/RelA RPKM ratio (Fig. 4e).

Examination of the DNA sequences underlying each Rel-preferential peak generally revealed multiple potential NF-κB motifs with variable PBM *z* scores for NF-κB dimers (Supplementary Table 3), with no consistent similarity to the tandem motifs described above for the *Il12b* promoter. The motifs that lead to preferential Rel binding and Rel-dependent transcription were difficult to predict. Nevertheless, the high Rel/RelA RPKM ratios at these peaks supported the hypothesis that preferential Rel binding is important for Rel-dependent transcription of the *Il12b* gene and a subset of other Rel-dependent genes.

### Analysis of Rel- versus RelA-preferential binding

The above results suggest that the most pronounced preferential Rel binding in vivo occurs at weak, nonconsensus motifs. To test this hypothesis further, we took advantage of a previous study in which we compiled the promoters of 65 lipid-A-induced genes that contained NF-κB motifs^38^. We ranked these 65 promoters by NF-κB PBM *z* score and then calculated their Rel/RelA ChIP–seq peak RPKM ratios. A high Rel/RelA RPKM ratio in vivo was strongly correlated (*P* = 0.004) with low in vitro PBM *z* scores (Fig. 4f and Supplementary Table 4). This strong correlation was especially striking given that the ChIP–seq results could not distinguish between homodimer and heterodimer binding and were also unable to consider possible impacts of interactions with other promoter-bound factors. These results further demonstrate that Rel-preferential binding in vivo is more pronounced at weaker motifs. We previously reported that RelA is more abundant than Rel in BMDMs^25^, which may help to explain why the Rel/RelA RPKM ratios were not even higher at Rel-preferential sites.

To further explore the significance of preferential binding, we examined the 30 promoter peaks that exhibited the greatest RelA-preferential binding or the greatest Rel-preferential binding. Among the 30 promoters with the greatest RelA-preferential binding, only two were at genes induced more than two-fold by lipid A (Supplementary Table 5); the other 28 genes exhibited either low expression (RPKM < 1) or were poorly induced, suggesting that these genes may not be functional NF-κB targets. By contrast, of the 30 promoters with the greatest Rel-preferential binding, 25 were at genes that exhibited lipid A induction >two-fold (Supplementary Table 5). Three of these top-30 genes corresponded to Rel-dependent genes defined above (*Il12b*, *Tnfsf9* and *Noct*). Five additional genes (*Plscr1*, *Tmem39a*, *Nudt17*, *Ccl12* and *Ptafr*) exhibited weaker Rel-dependent transcription. Thus, moderate to strong Rel-dependent transcription was observed at eight of the 30 genes whose promoters exhibited the greatest Rel-preferential promoter binding.

These findings add further evidence that Rel-preferential binding is often important for Rel-dependent transcription. Importantly, however, several other genes whose promoters showed strong Rel-preferential binding did not exhibit Rel-dependent transcription. Thus, although Rel-preferential promoter binding by ChIP–seq is common among Rel-dependent genes, it is not always sufficient. We propose that Rel-dependent transcription requires Rel-preferential binding combined with a promoter architecture that is conducive to Rel-dependent transcription. At promoters that exhibit Rel-preferential binding but not Rel-dependent transcription, other NF-κB dimeric species may bind with sufficient affinity for transcriptional induction.

### Rel- versus p50-preferential binding in vivo

**Fig. 5:**
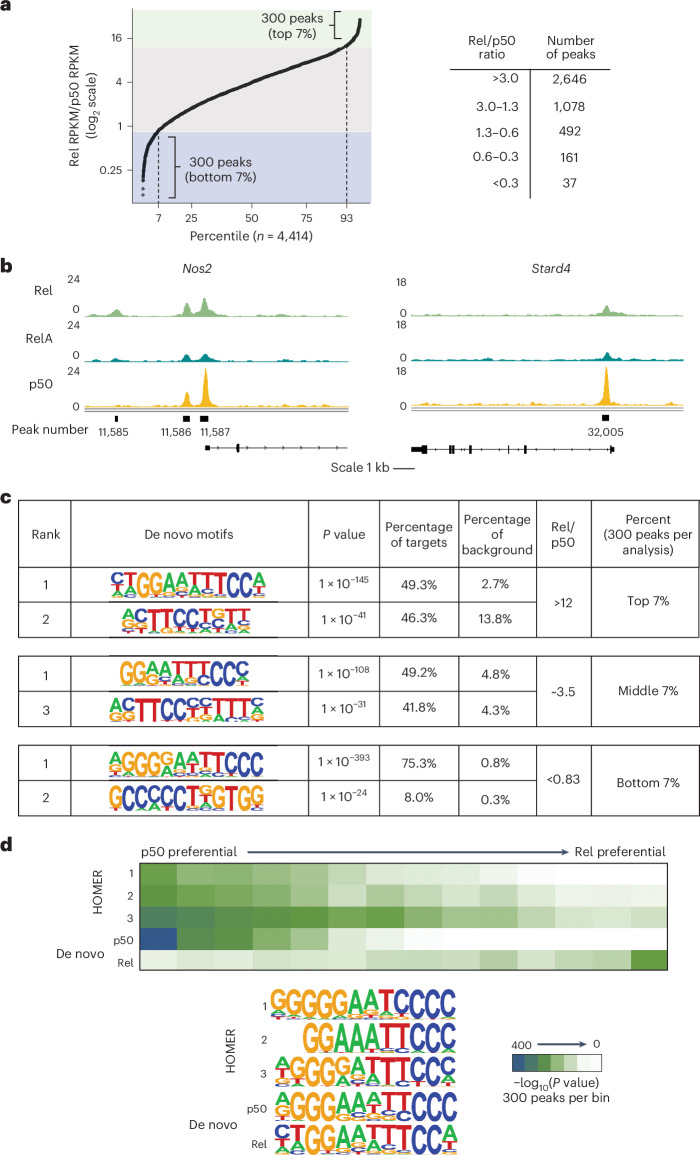
Selective binding of Rel and p50 in mouse BMDMs examined by ChIP–seq. **a**, ChIP–seq datasets obtained with antibodies against Rel and p50 in BMDMs stimulated with lipid A for 0 and 1 h were analyzed. The 2,891 peaks with the strongest peak scores obtained with each antibody at the 1-h time point were selected and merged, yielding 4,414 peaks (the peak number was smaller than in the RelA–Rel comparison because of the small number of peaks obtained with p50 antibody, probably owing to relatively poor antibody quality). Rel/p50 RPKM ratios were then calculated at each peak and are plotted on the *y* axis, with the 4,414 peaks along the *x* axis (displayed as a percentage of total peaks). The top and bottom 300 peaks based on ratio (7%) were then selected for motif analysis. On the right, numbers of peaks with different ranges of RPKM ratios are shown. **b**, IGV tracks are shown for ChIP–seq peaks in the promoter regions of the *Nos2* and *Stard4* genes. The *Nos2* peaks exhibited moderately preferential p50 binding in comparison with Rel and RelA, and the *Stard4* gene exhibited strongly preferential p50 binding. The RPKM scales are shown to the left, and gene annotations and annotated peak numbers are at the bottom. **c**, The top two most enriched motifs from de novo motif analyses performed with HOMER are shown for three different groups of peaks. Motif analysis was performed with 300 peaks (7%) representing the largest peak ratios, 300 peaks representing the smallest peak ratios and 300 peaks from the middle of the ratio distribution. **d**, The 4,414 peaks were divided into 15 bins of equal size across the spectrum of Rel/p50 RPKM ratios. The enrichment of the three consensus NF-κB motifs in HOMER, as well as one of the p50-preferential motifs and one of the Rel-preferential motifs identified in **b**, was then examined across the spectrum. NF-κB half-sites (not shown) were enriched equally across the spectrum, consistent with the presence of half-sites in all of the full consensus motifs examined.

To gain insight into possible relationships between Rel and the NF-κB p50 protein at Rel-dependent genes and genome-wide, we used the same approach as above to compare binding by Rel and p50. As p50 ChIP–seq yielded a much smaller number of peaks (possibly owing to lower antibody quality)^39^, this analysis was limited to the top 2,891 peaks obtained with each antibody, which, when merged, yielded 4,414 peaks (Fig. 5a). Among these peaks, Rel ChIP–seq signals (RPKM) were generally stronger than p50 signals, with 2,646 peaks exhibiting Rel/p50 RPKM ratios greater than 3 (Fig. 5a, right).

p50 ChIP–seq IGV tracks for promoter peaks at two Rel-dependent genes (*Il12b* and *Tnfsf9*) and at a third gene (*Camp*) are shown in Fig. 4e, with all three promoter regions exhibiting weak p50 signals. IGV tracks for two promoters exhibiting p50-preferential binding (*Nos2* and *Stard4*) are also shown in Fig. 5b. The absence of p50 peaks at the Rel-dependent promoters supports the hypothesis that they are primarily bound by Rel homodimers.

De novo motif analysis with 300 peaks showing the largest Rel preference in comparison with p50 revealed strong enrichment of a consensus sequence for Rel and RelA homodimer binding, with two G:C base pairs within each half-site (Fig. 5c, top). Enrichment of an NF-κB half-site motif was also observed, providing further evidence that Rel homodimers often bind sites that lack a dimeric consensus sequence (Fig. 5c). By contrast, motifs showing the smallest Rel/p50 RPKM ratios exhibited three G:C base pairs within each half-site, as preferred for p50 (ref. ^28^) (Fig. 5c, bottom). Strong enrichment was also observed for a motif containing five tandem G:C base pairs (Fig. 5c, bottom), which matched the motif with the greatest preference for p50 homodimers in prior in vitro PBM experiments^28^.

In an analysis of bins representing the spectrum of Rel/p50 RPKM ratios, three NF-κB consensus motifs exhibited their lowest enrichment in bins containing Rel-preferential peaks (Fig. 5d), again reflecting the ability of Rel homodimers to bind divergent motifs and half-sites. The greatest differences in motif enrichment between p50-preferential and Rel-preferential peaks were observed for p50 and Rel de novo motifs defined above (Fig. 5d).

We also examined the 30 promoters with the greatest p50-preferential binding (Supplementary Table 5). Almost all of these promoters contained motifs predicted by PBM analysis to support strong p50 homodimer binding. However, only two were associated with genes exhibiting RNA-seq RPKM > 1.0 and fold induction upon lipid A stimulation >2. Examination of RNA-seq data from *Nfkb1*^−/−^ BMDMs failed to reveal p50 dependence (Supplementary Table 5). These results are consistent with our recent analysis in which we found that p50-dependent genes did not coincide with strongly preferential p50 binding^39^.

To summarize, the Rel–p50 ChIP–seq comparison adds further evidence that binding preferences in vivo are aligned with in vitro binding preferences. Moreover, the results suggest that p50 binding is weak or absent at the promoters of Rel-dependent genes.

### Emergence of the DNA-binding difference during evolution

**Fig. 6:**
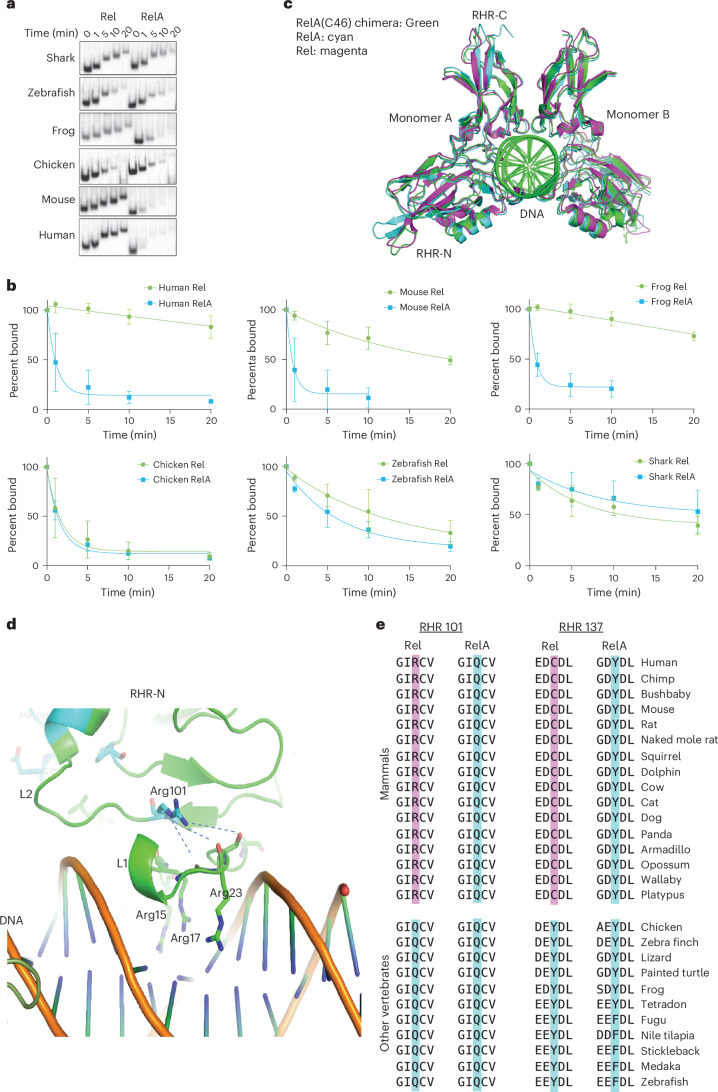
Late evolution of and structural analysis of Rel–RelA DNA-binding differences. **a**, RelA and Rel RHRs from six vertebrate species were expressed in HEK 293T cells. Nuclear extracts were prepared, and EMSA off-rate experiments were performed with a consensus NF-κB motif probe. After prebinding of the protein to the radiolabeled probe, a large excess of the same unlabeled oligonucleotide was added. At 0, 1, 5, 10 and 20 min after the addition of the unlabeled oligonucleotide, a sample was added to the native gel (complex migrations differed because the samples at different time points were loaded as the gel was running). As protein released from the radiolabeled probe is more likely to rebind the excess unlabeled oligonucleotide, the rate of loss of the protein–DNA complex provides an approximate measure of its half-life. **b**, Off-rate profiles from experiments performed with a consensus NF-κB motif. Complex abundances (*y* axis) are presented as the mean percentage (complex abundance at the 0-min time point was set to 100%) and standard deviation at each time point (*x* axis) from three independent experiments for each species. **c**, Comparison between the structure of the mouse RelA(C46) chimeric RHR homodimer (green), a mouse RelA RHR homodimer (cyan) and a mouse Rel RHR homodimer (magenta). RelA(C46) and RelA homodimers were bound to sequence GGGAATTTCC, whereas Rel homodimers were bound to a nonconsensus sequence, GGGTTTAAAGAAATTCCAGA. The N-terminal and C-terminal RHR regions (RHR-N and RHR-C, respectively), the two homodimer monomers and the DNA are labeled. **d**, Positioning of the Rel RHR Arg101 residue within one RHR monomer, relative to the L1 and L2 loops and DNA-interacting residues Arg15, Arg17 and Arg23. Possible stabilizing interactions are shown with dashed lines. **e**, Residues corresponding to Rel RHR Arg101 and Cys137, as well as two flanking residues on either side, for both RelA and Rel for 16 mammalian and 11 nonmammalian vertebrate species. Sequences were derived from the UCSC Genome Browser Comparative Genomics function. Source data

To test the hypothesis that the divergence of Rel and RelA in early gnathostomes was driven by amino acid changes that resulted in the DNA-binding affinity differences between Rel and RelA, we examined elephant shark, zebrafish, frog, chicken, mouse, and human Rel and RelA. After expression of these proteins in HEK 293T cells, competition time course experiments were performed (Fig. 6a). The proteins were prebound to a radiolabeled probe containing a consensus NF-κB motif. Then, an excess of an unlabeled oligonucleotide containing the same sequence was added, and the samples were loaded onto a polyacrylamide gel after different incubation times. When a bound protein dissociates from the radiolabeled probe, rebinding will generally be to the excess unlabeled oligonucleotide, such that the decline in the radiolabeled protein–DNA complex provides a measure of relative binding affinity.

**Extended Data Fig. 5:**
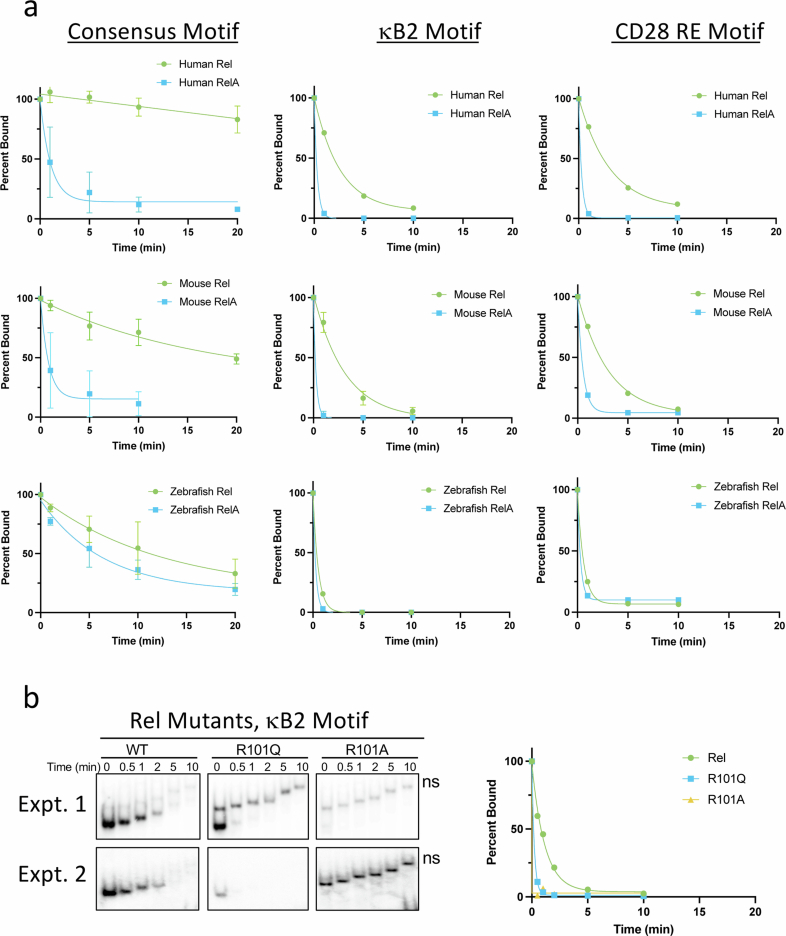
EMSA off-rate analysis at non-consensus NF-κB motifs. **a,** EMSA off-rate profiles are shown for Rel and RelA RHRs from representative species (human, mouse, and zebrafish) expressed in HEK293T cells. Profiles are shown for experiments performed with oligonucleotides containing the non-consensus *Il12b* κB2 motif (middle) and another non-consensus motif from the *Il2* locus (CD28 response element, right)^53^. Complex abundances (y-axis) are presented as the mean percentage (complex abundance at the 0-min time point set at 100%) at each time point (x-axis). Standard deviations are included when results represented the mean of three experiments, but not when the results represented the mean of two experiments. At the left, the profiles obtained with a consensus motif are reproduced from Fig. 6b for comparison. **b**, EMSA off-rate experiments are shown for Rel RHR mutant proteins expressed in HEK 293 T cells bound to the non-consensus *Il12b* κB2 motif. At the left, representative gel images are shown from two independent experiments. Note the appearance of a non-specific band (ns) that remains stable at all time points with the mutant proteins. Also note that the specific band is difficult to detect at the 0 min time point with the R101A mutant, due to the low affinity of the mutant protein for the non-consensus sequence. At the right, the EMSA off-rate profiles are shown for the Rel mutant proteins with the probe containing the *Il12b* κB2 motif. Complex abundances (y-axis) are presented as the mean percentage (complex abundance at the 0-min time point set at 100%) from two independent experiments at each time point (x-axis). Data from only one experiment for the R101A mutation are presented because the complex at the 0 min time point was too weak to quantify in other replicates). Source data

Using this approach with a consensus motif probe, we readily observed a large difference between mouse Rel and RelA (Fig. 6a,b; migration of the protein–DNA complex changed from lane to lane because samples from different time points were loaded as the gel was running). A similar difference was observed with the human and frog Rel and RelA orthologs. Surprisingly, however, RelA and Rel RHRs from elephant shark, zebrafish and chicken exhibited comparable off-rates (Fig. 6a,b). Notably, the large RelA–Rel difference observed in mice, humans and frogs appeared to be due to both increased Rel binding stability and decreased RelA binding stability in comparison with elephant shark, zebrafish and chicken. Similar results were obtained with a probe containing the nonconsensus *Il12b* κB2 motif and a second nonconsensus motif (CD28 RE) (Extended Data Fig. 5a). However, because of the faster off-rates when examining nonconsensus probes, the differences were more difficult to measure. These results suggest that the divergence of Rel and RelA in early vertebrates was not driven by the difference in DNA-binding affinity.

### Structural analysis of the Rel–RelA DNA affinity difference

To understand the difference in DNA affinity between Rel and RelA, we solved the structure of the mouse RelA(C46) chimera homodimer bound to a consensus NF-κB motif. A new structure was solved because prior RelA and Rel homodimer structures had been solved using different motifs, and because the prior Rel structure used chicken Rel^27^. We used the same motif (GGGAATTTCC) as that employed for a prior mouse RelA homodimer structure^40,41^, allowing us to compare two proteins (RelA and RelA(C46)) that differed by only the 46 residues that confer the affinity difference^25,28^.

The RelA(C46)–DNA complex structure was solved by molecular replacement using 2RAM as the search model and refined to 3.1 Å with an *R*_free_ of 32.88%. The structures of RelA(C46) (Fig. 6c, green) and RelA (cyan) were very similar. They were also highly similar to that of the chicken Rel homodimer (Fig. 6c, magenta) bound to a different sequence^27^, as well as to RelA homodimers bound to different sequences^40,41^. In addition, the relative orientation of the amino- and carboxy-terminal subdomains (RHR-N and RHR-C) of one monomer (Fig. 6c, monomer A, left) was very similar among Rel, RelA and RelA(C46). However, the orientation of the second monomer (monomer B, right) showed significant differences in the RelA and RelA(C46) complexes in comparison with the Rel complex. This difference was probably due to the use of a different DNA sequence for the Rel structure, as similar variations were observed with RelA bound to different sequences^40,41^.

**Extended Data Fig. 6:**
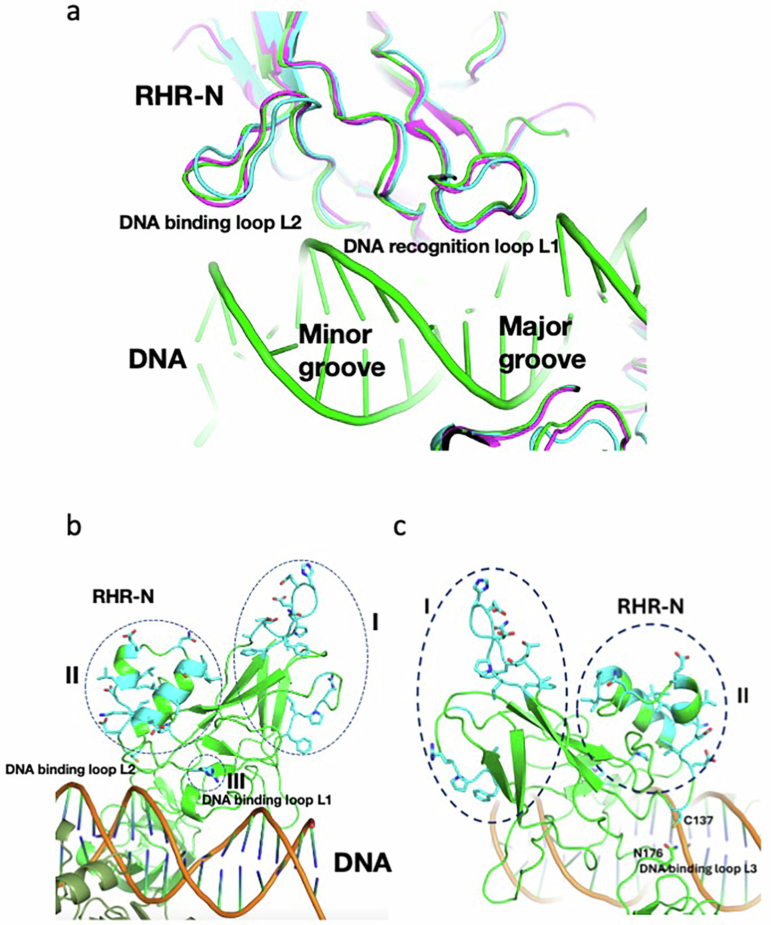
Structure insights into the Rel-RelA DNA affinity difference. **a**, The extensive similarity between the protein-DNA interfaces observed with the RelA (C46) (green), RelA (cyan), and Rel (magenta) proteins is shown. **b**, The locations of three clusters of Rel-specific RHR residues are highlighted with dashed circles and are labeled (I, II, and III). Region III consists of the R101 residue **c**, Reverse view of the structure displays the location of the mammal-specific C137 residue, which is in close proximity to DNA binding loop L3.

Focusing our attention on one monomer, we found that the DNA-binding surface structure was highly similar among the three complexes (Extended Data Fig. 6a). DNA-recognition loop L1 was positioned in the major groove similarly, and DNA-binding loop L2 was also similarly oriented. Furthermore, the direct DNA-binding surface residues of RelA(C46) and RelA were nearly identical. Thus, the different affinities are unlikely to be due to differences in the DNA-binding surface. Although many residues at the RelA(C46) DNA-binding surface showed different side-chain conformations from their RelA counterparts, RelA bound to different DNA sequences showed similar variations.

We identified Rel-specific residues on the RelA(C46) structure (Extended Data Fig. 6b). None of these Rel-specific residues contacted DNA directly. We next grouped the Rel-specific residues into three clusters. Cluster I residues were far away from the protein core and the DNA-binding surfaces and may have little impact on binding. Cluster II residues were involved in the packing interaction with the beta-sandwich core; this region is also connected with the DNA-binding loop L2 that binds DNA in the minor groove. These Rel-specific residues may therefore affect DNA binding by modulating the conformation flexibility of the protein fold and L2. Notably, the residue most likely to contribute to the increased binding affinity of mammalian Rel was Arg101, a sole residue in cluster III. The positively charged Arg101 is located behind the L1 loop, and its long side chain and guanidinium head form a network of hydrogen bonding interactions (dashed lines) with the backbone carbonyl groups on the L1 loop (Fig. 6d). This interaction could stabilize the three Arg residues of the L1 loop (Arg16, Arg18 and Arg 24) in a favorable conformation for DNA binding. In the RelA RHR, the corresponding residue is Gln, which can form fewer hydrogen bonds and with less conformational flexibility^42,43^.

Examination of Rel and RelA sequences from diverse species provided support for the importance of Arg101, as this residue was conserved in the Rel ortholog of mammalian species available in the UCSC Genome Browser Comparative Genomics function (Fig. 6e). Similarly, Gln101 was conserved in all of the mammalian RelA ortholog (Fig. 6e). However, in all nonmammalian vertebrates examined, RHR residue 101 was a Gln in both Rel and RelA orthologs (Fig. 6e). This suggests that the Gln101 to Arg101 change in a mammalian ancestor contributed to the affinity difference between Rel and RelA. Although a Rel–RelA affinity difference was also observed with *Xenopus laevis* proteins (Fig. 6a), the *X. laevis* Rel has a Gln at position 101, suggesting that other sequence changes may have led to the affinity difference in *X. laevis*.

Based on the above discovery, we searched for other residues in the 46-residue RHR region that consistently differed between Rel and RelA only in mammalian species. We found one other residue, Cys137, which was consistently a Cys in mammalian Rel proteins but was a large hydrophobic residue (Tyr or Phe) in nonmammalian Rel proteins and in all mammalian and nonmammalian RelA proteins (Fig. 6e). This residue was located near DNA-binding loop 3 (Extended Data Fig. 6c), but a role in DNA binding was more difficult to predict.

### Arg101 is critical for the Rel–RelA DNA affinity difference

**Fig. 7:**
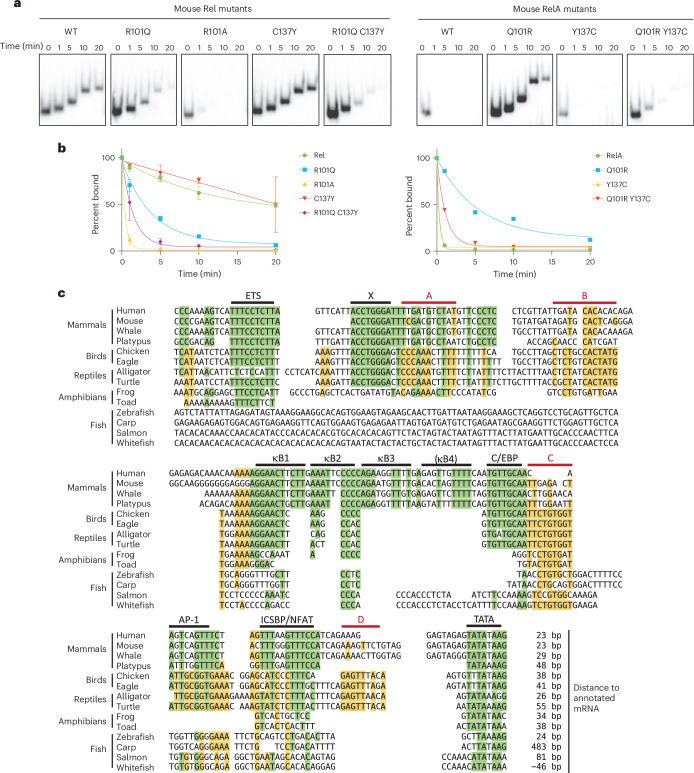
Contributions of Arg101 and Cys137 to the Rel–RelA affinity difference and *Il12b* promoter evolution. **a**, EMSA off-rate experiments were performed with WT and mutant Rel and RelA RHRs expressed in HEK 293T cells. Mutants included Rel R101Q, R101A, C137Y and R101Q C137Y, as well as RelA Q101R, Y137C and Q101R Y137C. RHR amino acid numbering is as in Extended Data Fig. 2a. **b**, Off-rate profiles from experiments performed with a consensus NF-κB motif for all mutant proteins examined. Complex abundances (*y* axis) are presented as the mean percentage (complex abundance at the 0-min time point was set to 100%) and standard deviation at each time point (*x* axis) from three independent experiments for Rel mutants. For RelA mutants, the mean percentages from two independent experiments are shown. **c**, Putative *Il12b* promoter sequences are compared for several vertebrate species. The origin of each sequence is described in Supplementary Table 1. The location of the most proximal nucleotide relative to the start of the annotated mRNA for each species is shown at the bottom right, with the expectation that the annotated mRNA start sites are often imperfect. Known and potential regulatory motifs that were conserved in all four mammalian species are highlighted in green, with green highlights shown when these nucleotides were conserved in other species. Potential regulatory motifs that exhibited stronger conservation in nonmammalian species are highlighted in yellow (red bars labeled A–D), with yellow highlights for these nucleotides when conserved in any species. Black horizontal bars represent motifs that have been shown to contribute to activity of the mouse or human *Il12b* promoters, in addition to two motifs that are highly conserved in mammalian species (X and κB4) but have not been examined for activity in a native chromosomal context. Source data

To determine whether Arg101 and/or Cys137 contributed to the affinity difference between Rel and RelA, we used a competition EMSA. Replacement of Rel Arg101 with Gln (R101Q) resulted in a faster off-rate with both a consensus motif (Fig. 7a,b) and the nonconsensus κB2 motif (Extended Data Fig. 5b). Replacement of Arg101 with Ala (Rel R101A) resulted in an even faster off-rate (Fig. 7a,b), consistent with the ability of Gln to form hydrogen bonds that cannot be formed by Ala. Binding of the Rel R101A protein to the *Il12b* κB2 motif was difficult to detect by EMSA, presumably owing to a low binding affinity. In contrast to the clear importance of Arg101, replacement of Rel Cys137 with Tyr (Rel C137Y) had no impact (Fig. 7a,b). However, combining C137Y with R101Q resulted in a moderately faster off-rate in comparison with R101Q alone (Fig. 7a,b).

An examination of mutations in the mouse RelA RHR confirmed the importance of Rel Arg101. Replacement of RelA Gln101 with Arg (RelA Q101R) resulted in a much slower off-rate (Fig. 7a,b). Conversely, replacing RelA Tyr137 with Cys (RelA Y137C) had no effect (Fig. 7a,b). Notably, combining Y137C with Q101R resulted in a faster off-rate in comparison with Q101R alone (Fig. 7a,b). Thus, the Y137C mutation appears to be incompatible with Q101R in the context of the RelA RHR, most likely reflecting the complex interactions that occur within the RHR.

These results establish a critical role for the mammalian Rel Arg101 residue in the difference in DNA-binding affinity between Rel and RelA. Other RHR residues, possibly including Cys137, must also contribute to the difference and may reflect a complex evolutionary path, based in part on epistatic considerations^16^, that ultimately resulted in the large affinity difference in mammals.

### *Il12b* promoter transitions during vertebrate evolution

Finally, we examined the *Il12b* promoter. The promoter was conserved in the four mammalian species shown in Fig. 7c and in all mammalian species examined. However, *Il12b* promoter sequences from nonmammalian vertebrates differed substantially, with complete absence of the κB3 and putative κB4 sequences and portions of the κB1 and κB2 motifs.

Further examination suggested that *Il12b* regulatory strategies may have undergone major transitions through vertebrate evolution. Sequences resembling a TATA box are apparent in all species (Fig. 7c). However, several other potential motifs that have been conserved through tens of millions of years of evolution appear to have undergone transitions. For example, hypothetical motif C is well conserved in fish, amphibians, reptiles and birds, but its conservation is diminished in mammals (Fig. 7c). In addition, a previously defined ETS motif^44^ is first apparent in amphibians and retained in reptiles, birds and mammals, whereas a previously defined C/EBP motif^45^ is first apparent in reptiles and retained in birds and mammals. These results point to dynamic changes in *Il12b* regulatory strategies, consistent with the observed changes in binding by NF-κB family members.

## Discussion

We combined biochemical, genomic, functional, structural and evolutionary analyses to examine a critical difference between NF-κB paralogs Rel and RelA. Our results add support to prior evidence^25^ that a key functional difference between the two proteins results from an intrinsic DNA-binding affinity difference rather than a specificity difference. In vivo, this affinity difference leads to the greatest Rel-preferential binding at nonconsensus motifs, perhaps because RelA interactions with these sites are especially unstable in vivo. Rel-dependent transcription appears to require preferential Rel binding combined with a suitable promoter/enhancer architecture. At *Il12b*, this promoter architecture consists of three tandem nonconsensus motifs, one of which diverges from the NF-κB consensus to such a great extent that its discovery relied on analyses of PBM datasets.

The Rel and RelA binding affinities appear to have diverged in mammalian ancestors along with major transitions in the promoter for at least one critical NF-κB target gene, *Il12b*. Comparison of the *Il12b* promoter provides a glimpse of possible broad transitions in *Il12b* regulatory strategies during vertebrate evolution; there are several putative motifs that appear to be highly conserved through tens of millions of years of evolution, but with the conservation gained or lost at defined evolutionary times. Importantly, these changes would need to accommodate the roles of Rel and RelA in regulating large numbers of other genes in diverse cell types.

The absence of the DNA-binding difference in early gnathostomes raises the question of which other properties of the two proteins drove their divergence and specialization. One possible contributor is the differential expression of the *Rela* and *Rel* genes^16,17^. Differences in the regulation of Rel and RelA nuclear translocation have also been described^46–50^. In addition, differences in their C-terminal transactivation domains may have contributed to their specialization. The transactivation domains of RelA and Rel are poorly conserved through evolution, and our prior chimeric protein studies showed that the C-terminal transactivation domains of RelA and Rel are interchangeable for *Il12b* activation in *Rel*^−/−^ macrophages^25^. Nevertheless, the RelA transactivation domain supports an interaction with the p300/CBP coactivators^14^^,^^51^, whereas the transactivation domain of Rel does not^22^. The p300/CBP interaction of RelA has been shown to be critical for the activation of a distinct subset of NF-κB target genes^52^.

Whether the Rel–RelA DNA-binding difference is critical in other immune cell types remains unknown. However, in support of this possibility, the first DNA motif proposed to confer Rel selectivity in T cells was the CD28 RE in the *Il2* gene^53^. Although both RelA- and Rel-containing dimers can bind to this motif, it possesses a nonconsensus sequence that may allow preferential binding in vivo by Rel homodimers.

Another unanswered question is why *Il12b* acquired a strong requirement for Rel in macrophages. A general hypothesis is that this allows expression of *Il12b* to be modulated differently from that of other inducible genes in physiological settings in which differential modulation is needed, via differential regulation of Rel activity. Examples include the modulation of *Il12b* in macrophages via selective nuclear translocation of Rel complexes upon induction of caspase 8 activity by microbial pathogens^49^. Selective modulation of Rel by very-long-chain ceramides has also been described^54^, although this study focused on later times after cell activation, when Rel has a broader role in inflammatory gene regulation.

Finally, the absence of the DNA-binding difference between RelA and Rel homodimers in early vertebrates raises the question of whether *Il12b* induction in early vertebrates remains Rel dependent, albeit with the Rel dependence due to a different mechanism. Possibly related to this question, *Il12b* activation in mammals is Rel independent in some dendritic cell populations^55^. Thus, *Il12b* activation in early vertebrates may rely on Rel-independent mechanisms that remain intact in mammals in some cell types but have been enhanced by a Rel-dependent regulatory mechanism in macrophages.

## Methods

### Mice

WT C57BL/6 mice were from The Jackson Laboratory, and *Rel*^−/−^ mice in a C57BL/6 background^18^ were a gift from S. Gerondakis (Monash University). Mice were used for preparation of BMDMs and were maintained by the UCLA Division of Laboratory Animal Medicine under standard housing conditions in accordance with all federal, state and local guidelines. Experiments were performed following approval by the UCLA Chancellor’s Animal Research Committee (protocol no. 1999-073).

### Analysis of NF-κB RHRs from diverse species

The phylogeny was estimated with BAli-Phy v.4.0-beta15 (ref. ^56^) using the LG08 + R4 + HB02 model of amino acid substitutions and the RS07 model of insertions and deletions. The HB02 model^57^ allows for local conservation of amino acid residues on the tree without requiring them to be conserved in all taxa. We ran five chains for 100,000 iterations each and pooled the results. The average standard deviation of split frequencies for the resulting ensemble was 0.009, indicating that convergence of tree topologies was reached.

cDNAs were amplified from messenger RNA (mRNA) or cDNA libraries by RT–PCR or PCR and cloned into the pcDNA3 vector with an N-terminal Flag epitope tag. Rel and RelA RHR cDNAs were from mouse, human, chicken, frog, zebrafish and elephant shark. The sequences were confirmed to match those shown in Supplementary Table 2. The recombinant proteins were overexpressed in HEK 293T cells (ATCC CRL-3216) using Lipofectamine 3000 (Invitrogen). Briefly, 5 μg DNA and 6 μl lipofectamine was used to transfect subconfluent cells in a 35-mm tissue-culture well. Forty-eight hours posttransfection, nuclear extracts were prepared for EMSA^25,45^. Briefly, cells were harvested by trypsinization and washed with Dulbecco’s phosphate-buffered saline (Corning, MT21031CV), followed by resuspension and incubation for 15 min on ice in 400 μl of cold buffer (10 mM HEPES at pH 7.9, 10 mM KCl, 0.1 mM EGTA, 0.1 mM EDTA, 1 mM dithiothreitol (DTT), 1 mM phenylmethylsulfonyl fluoride, 1× SIGMAFAST protease inhibitor cocktail (Sigma, S8830-20TAB)). Then, 10% NP-40 (25 μl) was added, followed by mixing vigorously for 10 s, then centrifugation at 12,000 rpm for 1 min. The pelleted nuclei were suspended in 200 μl cold buffer C (20 mM HEPES at pH 7.9, 420 mM NaCl, 1.5 mM MgCl_2_, 0.2 mM EDTA, 25% glycerol, 1 mM DTT, 1 mM phenylmethylsulfonyl fluoride, 1× SIGMAFAST protease inhibitor cocktail) and subjected to periodic pipetting and agitation for 20 min on ice, followed by centrifugation (12,000 rpm for 5 min at 4 °C). Extract amounts were titrated by EMSA to achieve abundant binding before the off-rate EMSA experiments were performed.

### X-ray crystallography

RelA(C46) RHR chimeric protein was expressed from the pET11a expression vector in *Escherichia coli*, and the protein was purified on a Q-Sepharose High Performance anion-exchange column (GE Healthcare) and a SP Sepharose High Performance cation-exchange column (GE Healthcare)^28^. The protein was crystallized at 18 °C using the hanging-drop, vapor-diffusion method with a reservoir solution of 0.1 M bis-tris propane (pH 6.5), 0.2 M sodium/potassium phosphate, 20% polyethylene glycol 3350, 10 mM DTT and 0.5% beta octyl glucoside. X-ray data were collected at ALS beamline 8.2.2. Data were processed and scaled with the HKL2000 software package^58^. Initial phase information was obtained by the molecular replacement method with Phaser^59^ using the crystal structure of NF-κB RelA (PDB ID: 2RAM)^41^ as the search model. The structural model was refined using PHENIX^60^ and modified with COOT^61^. Data collection, phasing and refinement statistics are summarized in Supplementary Table 6. PyMOL v.2.5.6 was used to generate the figures. The structural information has been deposited in the Protein Data Bank (PDB ID: 8U9L). Comparisons are shown with DNA-bound RelA homodimers (PDB ID: 2RAM)^41^ and Rel homodimers (PDB ID: 1GJI)^27^.

### PBM and SPR experiments

The PBM profiles were prepared from previously reported datasets^28^. SPR was performed on a Biocore T100 (GE Healthcare) instrument using streptavidin chips (Sensor Chip SA)^28^. Synthetic biotinylated oligonucleotides were immobilized on the chips in running buffer (10 mM HEPES, pH 7.5, 150 mM NaCl, 3 mM EDTA, 0.005% Tween 20). Protein samples^28^ were applied at 50 μl min^−1^ at 10 °C. Binding data were collected in the running buffer. The chip surface was regenerated with a 90-s pulse of 2 M NaCl and a 180-s pulse of running buffer. Scrubber 2 (BioLogic Software) was used to obtain dissociation rates by global fitting of the kinetic data and a 1:1 binding model. The half-life values presented are directly proportional to dissociation off-rates (*t*_1/2_ = ln(2)/*k*_off_). Different concentrations of each protein were used, ranging from 1 nM to 1 μM (ref. ^28^). The double-stranded oligonucleotides examined by SPR were as follows:

GGGGGGAGAT: CCTAACATCA**GGGGGGAGAT**TGATGTTAGG

GGGGGTTTTT: CCTAACATCA**GGGGGTTTTT**TGATGTTAGG

κB1: CCTAACATCA**GGAACTTCTT**TGATGTTAGG

κB2: CCTAACATCA**AAAATTCCCC**TGATGTTAGG

κB3: CCTAACATCA**AGAATGTTTT**TGATGTTAGG.

### Transient transfection experiments

The mouse *Il12b* promoter (−355 to +55) was cloned into the pGL4.10 vector (Promega). Motif mutations were introduced using the GENEART site-directed mutagenesis system (Invitrogen). C-terminal single Flag-tag versions of full-length cDNAs encoding mouse RelA, Rel and p50 were cloned into the pcDNA3 vector. HEK 293T cells, grown in Dulbecco’s modified Eagle medium (DMEM) with 10% fetal bovine serum (FBS), were transfected with Lipofectamine 2000 (Thermo Fisher) in 24-well plates. For these transfections, 1–8,000 ng of the expression plasmid was cotransfected with 20 ng of the *Il12b*-pGL4 vector; 1 ng of a TK–Renilla luciferase vector was included as a transfection control. Cells were collected 24 h posttransfection, and firefly and Renilla luciferase were analyzed with a Dual Luciferase Reporter Assay system (Promega). Normalization was performed using western blot analysis for protein concentration, Renilla luciferase for transfection efficiency, and empty vector firefly and Renilla values for background signal.

### Engineering and analysis of mutant *Il12b*–*Gfp* BACs

Recombination in *E. coli*^62^ was used to introduce an *Egfp* cDNA into the second exon of a 200-kb BAC from C57BL/6 mice spanning the *Il12b* locus (RP23-417p8 (Children’s Hospital Oakland Research Institute)). Recombination in *E. coli* was then used to introduce substitution mutations into *Il12b* promoter motifs (Extended Data Fig. 4b). The recombinant WT and mutant *Il12b*–*Egfp* BACs were introduced into a mouse embryonic stem cell (ES cell) line (R1 ES cells (ATCC SCRC-1011)). Clonal lines were expanded, and single-copy integrants were identified by qPCR and Southern blot analyses of genomic DNA isolated from each clone.

BAC-containing ES cells were differentiated into myeloid progenitors and macrophages^63^, with confirmation of differentiation by flow cytometry (Extended Data Fig. 4b). Briefly, R1 ES cells were maintained in ES media (ESMM: knockout DMEM plus 1% l-glutamine, 1% penicillin–streptomycin, 1% nonessential amino acids (Gibco), 15% ES-certified FBS (Omega Scientific) and 1,000 units ml^−1^ ESGRO leukemia inhibitory factor (Millipore)), with mitogen-C-inactivated primary mouse embryonic fibroblasts on gelatin-coated tissue-culture flasks (Corning). Two days before the start of differentiation, cells were plated in Iscove’s modified Dulbecco’s medium (IMDM)-ES (IMDM base medium (CellGro), 15% FBS (Omega Scientific), 1,000 units ml^−1^ ESGRO, 0.15 mM monothioglycerol (Sigma), 1% penicillin–streptomycin and 1% l-glutamine) on gelatin-coated plates without feeder cells. Media was changed the next day.

After 2 days, cells were harvested by trypsinization and washed twice with IMDM-Wash medium (IMDM-ES medium without FBS), and 5 ml of cells (2,000 cells per ml) in EB media (IMDM base medium (CellGro), 15% FBS, 0.4 mM monothioglycerol (Sigma), 1% penicillin–streptomycin, 2% l-glutamine, 300 μg ml^−1^ transferrin (Roche), 50 μg ml^−1^ ascorbic acid (Sigma) and 5% protein-free hybridoma medium (Gibco)) were plated in 60 × 15 mm petri plates (Parter Medical Products Inc.). Embryoid bodies on day 6 were dissociated by trypsinization, washed twice in IMDM-Wash medium and resuspended at 1 million cells per ml in macrophage medium 1 (IMDM base medium, 10% FBS, 0.15 mM monothioglycerol, 1% penicillin–streptomycin, 1% l-glutamine, 5% CMG medium (M-CSF conditioned medium) and 1 ng ml^−1^ IL-3). Cells (10 ml) were plated in 100 × 20mm tissue-culture plates (Corning) for 48 h to promote proliferation of hematopoietic progenitor cells. After 48 h, culture supernatants harboring hematopoietic progenitors were pelleted by centrifugation, resuspended in 10 ml of macrophage medium 2 (macrophage medium 1 without IL-3) and plated in new 100 × 20 mm tissue-culture plates (Corning) for 6 days. On day 6, ES-cell-derived macrophages were evaluated for expression of cell-surface markers (F4/80 and CD11B) by flow cytometry or stimulated with lipid A (100 ng ml^−1^) for 2 h for gene expression analysis by qRT–PCR. The PCR primers were as follows:

*Egfp* forward: ACGACGGCAACTACAAGACC

*Egfp* reverse: GTCCTCCTTGAAGTCGATGC

*Il12b* forward: AGCCACTCACATCTGCTGCT

*Il12b* reverse: AACCGTCCGGAGTAATTTGG.

### EMSAs

Purified proteins (Figs. 2f and 3a,b and Extended Data Fig. 3) or nuclear extracts from transfected HEK 293T cells (Figs. 6a,b and 7a,b and Extended Data Fig. 5) were incubated with limiting amounts of double-stranded ^32^P-radiolabeled probe (about 10^−11^ M) in a total volume of 25 μl containing 10 mM Tris-HCl, pH 7.5, 150 mM NaCl, 1 mM DTT, 1 mM EDTA, 5% glycerol, 80 ng μl^−1^ dI-dC and 200 ng μl^−1^ bovine serum albumin^25,45^. Each reaction was incubated at 4 °C for 30 min before being loaded into wells of 5% native polyacrylamide gels (bis-acrylamide 40%, 19:1, Fisher BP1406-1). Electrophoresis was performed at 150 volts in the gel for 90 min. Dried gels were exposed to a phosphor imager screen (Amersham Bioscience) and scanned in a phosphor imager (Amersham Typhoon, GE Healthcare)^25,45^. Band intensities were quantified using ImageQuant (GE). For off-rate experiments, a 100-fold excess of unlabeled double-stranded consensus oligonucleotide was added to the preformed protein–probe complex, followed by incubation for the indicated time. Double-stranded oligonucleotide probes were as follows (bold text designates the NF-κB recognition motif in some probes):

consensus NF-κB: CCTAACATCA**GGAATTTCC**TGATGTTAGG or

CCTAACATCA**GGGAATTTCC**TGATGTTAGG

κB1: CCTAACATCA**GGAACTTCTT**TGATGTTAGG

κB2: CCTAACATCA**AAAATTCCCC**GATGTTAGG

κB3: CCTAACATCA**AGAATGTTTT**TGATGTTAGG

κB4: CCTAACATCA**CACTAGTTTTT**GATGTTAGG

κB3m: CCTAACATCAA**GAATGAAAT**TGATGTTAGG

κB1 + 2 + 3: AGGAACTTCTTAAAATTCCCCCAGAATGTTTTGACA

κB1m + 2 + 3: ACCAACTTCTTAAAATTCCCCCAGAATGTTTTGACA

κB1 + 2 m + 3: AGGAACTTCTTAAAATTAAACCAGAATGTTTTGACA

κB1 + 2 + 3 m: AGGAACTTCTTAAAATTCCCCCAGAATGAAATGACA

CD28 RE: TGGGGGTTTAAAGAAATTCCAGAGAGTCATCAG.

### Nascent transcript RNA-seq

BMDMs were prepared from 8–10-week old male C57BL/6 and *Rel*^−/−^ mice^38^. Macrophages were activated on day 6 with lipid A for the time indicated in each experiment (Sigma-Aldrich). Nascent transcript RNA-seq was performed, and the data were analyzed^38,64^. Two replicates for each time point and genotype were used in the analysis. Following sequencing, reads were aligned to the NCIB37/mm9 mouse genome (Ensembl v.67) using HISAT v.2.1.1 (refs. ^65,66^). SAMtools v.1.19.2 was used to compress and sort sequencing data^67^. SeqMonk (Babraham Bioinformatics) v.1.48.0 was used to extract raw read counts from BAM files. Raw counts were then normalized by sequencing depth (million reads) and peak size (kbp) to generate RPKMs. For statistical analysis, raw counts for the WT and *Rel*^−/−^ conditions were analyzed by DESeq2 to generate adjusted *P* values^68^. Expressed genes with an RPKM > 3 at any time point in WT cells stimulated with lipid A were plotted (*n* = 2,980 genes). Fold change was calculated by averaging the RPKMs for the two replicates and dividing the average *Rel*^−/−^ RPKM by the average WT RPKM.

### ChIP–seq analysis

ChIP–seq was performed^38^^,^^39^^,^^69^ with anti-RelA, anti-Rel and anti-p50 antibodies (Cell Signaling, Inc., 8242, 68489 and 13586, respectively; 1:1,000 dilutions). Approximately 10 million BMDMs were used per sample from C57BL/6 male mice aged 8–12 weeks. After cross-linking with 1 mM disuccinimidyl glutarate and 1% formaldehyde, cells were sonicated on a Covaris M220 focused ultrasonicator to 200–500-bp DNA fragments. ChIP–seq libraries were prepared and sequenced^38,69^. Reads were aligned using HISAT v.2.1.1 to the NCIB37/mm9 mouse genome (Ensembl v.67)^65,66^. Following alignment, SAM files were compressed and sorted using SAMtools v.1.19.2 (ref. ^67^). Peaks were called using HOMER v.4.11 software^70^. To compare peaks across multiple samples, a master probe was generated with BEDTools v.2.31.0 (ref. ^71^). Each ChIP sample was compared with the input sample generated in parallel for accurate global and local background. Peaks were called if the false discovery rate was less than 0.01. To generate RPKMs, SeqMonk v.1.48.0 was used to extract raw reads from the BAM files. Raw read counts were then normalized to the sequencing depth and size of the peak. Motif analysis was performed with HOMER v.4.11 (ref. ^70^). Motifs were found in a window of ± 200 bp from the center of the peak. Statistical analysis was performed using HOMER v.4.11 with a differential motif discovery algorithm that used a zero or one occurrence per sequence scoring method combined with binomial enrichment calculations to determine motif enrichment^70^. Only reproducible peaks were analyzed. For Rel and p50 ChIP–seq, three biological replicates were analyzed. Reproducibility was defined as a peak score >19 and RPKM > 3 in two of three replicates. For RelA ChIP–seq, we analyzed five biological replicates, with reproducibility defined as peak score >19 and RPKM > 3 in three of five samples.

### Reporting summary

Further information on research design is available in the Nature Portfolio Reporting Summary linked to this article.

## Online content

Any methods, additional references, Nature Portfolio reporting summaries, source data, extended data, supplementary information, acknowledgements, peer review information; details of author contributions and competing interests; and statements of data and code availability are available at 10.1038/s41590-025-02138-2.

## Supplementary information

Supplementary Information Supplementary Tables 1–6.

Reporting Summary

## Source data

Source Data Fig. 6 ImageQuant values from each replicate examining protein off-rates.

Source Data Fig. 7 ImageQuant values from each replicate examining protein off-rates.

Source Data Extended Data Fig. 5 ImageQuant values from each replicate examining protein off-rates.

## Data Availability

All sequencing data referred to in this manuscript are publicly available at the NCBI Gene Expression Omnibus (SuperSeries GSE243012). RNA-seq data are available under accession GSE243011. ChIP–seq data are available under accession GSE243010. Two of the RelA ChIP–seq replicates with lipid A stimulation for 1.0 h are available under accession GSE67357. The X-ray crystallographic data are available at the Protein Data Bank (PDB ID: 8U9L). Source data are provided with this paper.

## Code availability

No custom code was used in this study.
