# Supplementary material for: Stepwise neofunctionalization of the NF-κB family member Rel during vertebrate evolution
Source: Nat Immunol. 2025 Apr 30;26(5):760–74. doi: 10.1038/s41590-025-02138-2 (PMC12043515; doi:10.1038/s41590-025-02138-2)
Supplement: Supplementary file 1 — Supplementary Tables 1–6. [file 41590_2025_2138_MOESM1_ESM.pdf]

nature immunology

Supplementary information

https://doi.org/10.1038/s41590-025-02138-2

Stepwise neofunctionalization of the NF-κB family member Rel during vertebrate evolution

In the format provided by the authors and unedited

Supplementary Table 1. Identifying information for Rel/NF-κB RHR sequences and *Il12b* promoter sequences

Rel/NF-κB RHR Sequences (relevant to Fig. 1a, Fig. 6a, Extended Data Figs. S1 and S2 and Supplementary Table 2)

Species	Protein	Accession number
Human (*Homo sapiens*)	Rel	XP_016860116.1
	RelA	NP_001391587.1
	RelB	NP_006500.2
	Nfkb1	NP_001369557.1
	Nfkb2	NP_001070962.1
Mouse (*Mus musculus*)	Rel	NP_033070.2
	RelA	NP_033071.1
	RelB	NP_033072.2
	Nfkb1	NP_032715.2
	Nfkb2	NP_001170841.1
Chicken (*Gallus gallus*)	Rel	NP_001161198.2
	RelA	XM_02145178.1 (UCSC Genome Browser)
	RelB	AAD41539.1
	Nfkb1	NP_001383324.1
	Nfkb2	XP_046775795.1
Frog (*Xenopus laevis*)	Rel	AAI70187.1
	RelA	XP_018112002.1
	RelB	NP_001079335.1
	Nfkb1	NP_001087808.1
	Nfkb2	XP_018082331.1
Zebrafish (*Danio rerio*)	Rel	NP_001001841.2
	RelA	NP_001001839.2
	RelB	XP_021322023.1
	Nfkb1	XP_021336945.1
	Nfkb2	NP_001001840.2
Elephant shark (*Callorhinchus milii*)	Rel	XP_007890564.1
	RelA	XP_042202691.1
	RelB	cDNA cloned for this study.
	Nfkb1	XP_007890062.2
	Nfkb2	XP_007897340.2
	Nfkb4	XP_07904454.1
Thorny skate (*Amblyraja radiata*)	Rel	XP_032881051.1
	RelA	Not found
	RelB	XP_032870316.1
	Nfkb1	XP_032876253.1
	Nfkb2	XP_032868306.1
	Nfkb3	XP_032885971.1
Lamprey (*Petromyzon marinus*)	Rel1	XP_032811903.1
	Rel2	XP_032830134.1
	Rel3	XP_032804357.1
	Rel4	XP_032818672.1
	Nfkb	XP_032825135.1

Hagfish (*Eptatretus atami*)	Rel1, Rel2, Rel3, Rel4, and Nfkb<br>From NCBI tblastx search and open reading frame analyses of Eptata_v1 genome using lamprey cDNA accession numbers and sequences.<br>BioProject PRJNA953751	
Ciona (*Ciona intestinalis*)	Rel	XP_026692312.1
	Nfkb	NP_001071772.1
Sea Pineapple (*Halocynthia roretzi*)	Rel	BAB47172.1
Lancelet (*Branchiostoma belcheri*)	Rel	XP_019636590.1
Sea urchin (*Strongylcentrotus purpuratus*)	Rel	XP_030854803.1
	Nfkb	NP_999819.1
Drosophila (*Drosophila melanogaster*)	Dorsal	NP_001286014.1
	Dif	NP_001162999.1
	Relish	NP_477094.1

***<u>Il12b</u>* <u>Promoter Sequences (Accessed via UCSC Genome Browser; relevant to Fig. 7c)</u>**

Human	mRNA	NM_002187.3
Homo sapiens	Genome	GRCh38/hg38
Mouse	mRNA	NM_001303244.1
Mus musculus	Genome	GRCm39/mm39
Northern Pacific Minke Whale	mRNA	XM_07171442.1
Balaenoptera acutorostrata scammony	Genome	BalAcu1.0<br>RefSeq: GCF_000493695.1<br>KI537408
Platypus	mRNA	XM_029051623.2
Ornithorhynchus anatinus	Genome	mOrnAna1.pri.v4<br>RefSeq: GCF_004115215.2<br>NC_041749.1
Chicken	mRNA	AJ564201
Gallus gallus	Genome	GRCg6a 2018<br>RefSeq: GCF_000002315.6<br>NC_006100.5
Bald Eagle	mRNA	XM_010579102.1
Haliaeetus leucocephalus	Genome	*Haliaeetus_leucocephaus*-4.0<br>RefSeq: GCF_000737465.1<br>NW_010973092.1
Chinese Alligator	mRNA	Augustus prediction g2915.11
Alligator sinensis	Genome	ASM45574v1 Aug. 2013<br>RefSeq: GCF_000455745.1<br>NW_005841977.1

Green Sea Turtle<br>*Chelonia mydas*	mRNA<br>Genome	XM_007057267.4<br>rCheMyd1.pri.v2 Jul. 2021<br>RefSeq: GCF_015237465.2<br>NC_057854.1
Western Clawed Frog<br>*Xenopus tropicalis*	mRNA<br><br>Genome	Ensemble ENSXETT00000119870<br>(Xenbase.org)<br>*Xenopus tropicalis* 10.0-Xenbase<br>UCB_Xtro_10.0/xenTro10
Common Toad<br>*Bufo bufo*	mRNA<br>Genome	XM_040439804.1<br>aBufBuf1.1 Jan. 2021<br>RefSeq: GCF_905171765.1<br>NC_053389.1
Zebrafish<br>*Danio rerio*	mRNA<br>Genome	NM_001007108.1<br>GRCz11 May 2017<br>RefSeq: GCF_000002035.6<br>NC_007125.7
Crucian Carp<br>*Carassius carassius*	mRNA<br>Genome	XM_059516704.1<br>fCarCar2.1 Aug. 2023<br>RefSeq: GCF_963082965.1<br>NC_081783.1
Pink Salmon<br>*Oncorhynchus gorbuscha*	mRNA<br>Genome	NM_001124392<br>OgorEven_v1.0 Dec. 2021<br>RefSeq: GCF_021184085.1
Lake Whitefish<br>*Coregonus clupeaformis*	mRNA<br>Genome	NM_0418899908.1<br>ASM2061545v1 Nov. 2021<br>RefSeq: GCF_020615455.1<br>NC_059207.1

Supplementary Table 2. RHR Sequences for Phylogenetic Analysis

```
>HumanRel
PYIEIIEQPRQRGMRFRYKCEGRSAGSIPGEHSTDNNRTYPSIQIMNYYGKGKVRITLVTKNDPYKPHPHDLVGKDC
RDGYYEAEFGQERRPLFFQNLGIRCVKKKEVKEAIITRIKAGINPFNVPEKQLNDIEDCDLNVVRLCFQVFLPDEHG
NLTTALPPVVSNPIYDNRAPNTAELRICRVNKNCGSVRGGDEIFLLCDKVQKDDIEVRFVLNDWEAKGIFSQADVHR
QVAIVFKTPPYCKAITEPVTVKMQLRRPSDQEVSESMDFRYLPDEKDTYGNKAKKQ
>HumanRelA
PYVEIIEQPKQRGMRFRYKCEGRSAGSIPGERSTDTTKTHPTIKINGYTGPGTVRISLVTKDPPHRPHPHELVGKDC
RDGFYEAELCPDRCIHSFQNLGIQCVKKRDLEQAISQRIQTNNNPFQVPIEEQRGDYDLNAVRLCFQVTVRDPSGRP
LRLPPVLSHPIFDNRAPNTAELKICRVNRNSGSCLGGDEIFLLCDKVQKEDIEVYFTGPGWEARGSFSQADVHRQVA
IVFRTPPYADPSLQAPVRVSMQLRRPSDRELSEPMEFQYLPDTDDRHRIEEKRK
>HumanRelB
PHLVITEQPKQRGMRFRYECEGRSAGSILGESSTEASKTLPAIELRDCGGLREVEVTACLVWKDWPHRVHPHSLVGK
DCTDGICRVRLRPHVSPRHSFNNLGIQCVRKKEIEAAIERKIQLGIDPYNAGSLKNHQEVDMNVVRICFQASYRDQQ
GQMRRMDPVLSEPVYDKKSTNTSELRICRINKESGPCTGGEELYLLCDKVQKEDISVVFSRASWEGRADFSQADVHR
QIAIVFKTPPYEDLEIVEPVTVNVFLQRLTDGVCSEPLPFTYLPRDHDSYGVDKKRK
>HumanNfkb1
PYLQILEQPKQRGFRFRYVCEGPSHGGLPGASSEKNKKSYPQVKICNYVGPAKVIVQLVTNGKNIHLHAHSLVGKHC
EDGICTVTAGPKDMVVGFANLGILHVTKKKVFETLEARMTEACIRGYNPGLLVHPDLAYLQAEGGGDRQLGDREKEL
IRQAALQQTKEMDLSVVRLMFTAFLPDSTGSFTRRLEPVVSDAIYDSKAPNASNLKIVRMDRTAGCVTGGEEIYLLC
DKVQKDDIQIRFYEEEENGGVWEGFGDFSPTDVHRQFAIVFKTPKYKDINITKPASVFVQLRRKSDLETSEPKPFLY
YPEIKDKEEVQRKRQ
>HumanNfkb2
PYLVIVEQPKQRGFRFRYGCEGPSHGGLPGASSEKGRKTYPTVKICNYEGPAKIEVDLVTHSDPPRAHAHSLVGKQC
SELGICAVSVGPKDMTAQFNNLGVLHVTKKNMMGTMIQKLQRQRLRSRPQGLTEAEQRELEQEAKELKKVMDLSIVR
LRFSAFLRASDGSFSLPLKPVISQPIHDSKSPGASNLKISRMDKTAGSVRGGDEVYLLCDKVQKDDIEVRFYEDDEN
GWQAFGDFSPTDVHKQYAIVFRTPPYHKMKIERPVTVFLQLKRKRGGDVSDSKQFTYYPLVEDKEEVQRKRR
>MouseRel
PYVEIIEQPRQRGMRFRYKCEGRSAGSIPGERSTDNNRTYPSVQIMNYYGKGKIRITLVTKNDPYKPHPHDLVGKDC
RDGYYEAEFGPERRPLFFQNLGIRCVKKKEVKEAIILRISAGINPFNVPEQQLLDIEDCDLNVVRLCFQVFLPDEDG
NFTTALPPIVSNPIYDNRAPNTAELRICRVNKNCGSVRGGDEIFLLCDKVQKDDIEVRFVLNDWEARGVFSQADVHR
QVAIVFKTPPYCKAILEPVTVKMQLRRPSDQEVSESMDFRYLPDEKDAYGNKSKKQ
>MouseRelA
PYVEIIEQPKQRGMRFRYKCEGRSAGSIPGERSTDTTKTHPTIKINGYTGPGTVRISLVTKDPPHRPHPHELVGKDC
RDGYYEADLCPDRSIHSFQNLGIQCVKKRDLEQAISQRIQTNNNPFHVPIEEQRGDYDLNAVRLCFQVTVRDPAGRP
LLLTPVLSHPIFDNRAPNTAELKICRVNRNSGSCLGGDEIFLLCDKVQKEDIEVYFTGPGWEARGSFSQADVHRQVA
IVFRTPPYADPSLQAPVRVSMQLRRPSDRELSEPMEFQYLPDTDDRHRIEEKRK
>MouseRelB
PYLVITEQPKQRGMRFRYECEGRSAGSILGESSTEASKTLPAIELRDCGGLREVEVTACLVWKDWPHRVHPHSLVGK
DCTDGVCRVRLRPHVSPRHSFNNLGIQCVRKKEIEAAIERKIQLGIDPYNAGSLKNHQEVDMNVVRICFQASYRDQQ
GHLHRMDPILSEPVYDKKSTNTSELRICRINKESGPCTGGEELYLLCDKVQKEDISVVFSTASWEGRADFSQADVHR
QIAIVFKTPPYEDLEISEPVTVNVFLQRLTDGVCSEPLPFTYLPRDHDSYGVDKKRK
>MouseNfkb1
PYLQILEQPKQRGFRFRYVCEGPSHGGLPGASSEKNKKSYPQVKICNYVGPAKVIVQLVTNGKNIHLHAHSLVGKHC
EDGVCTVTAGPKDMVVGFANLGILHVTKKKVFETLEARMTEACIRGYNPGLLVHSDLAYLQAEGGGDRQLTDREKEI
IRQAAVQQTKEMDLSVVRLMFTAFLPDSTGSFTRRLEPVVSDAIYDSKAPNASNLKIVRMDRTAGCVTGGEEIYLLC
DKVQKDDIQIRFYEEEENGGVWEGFGDFSPTDVHRQFAIVFKTPKYKDVNITKPASVFVQLRRKSDLETSEPKPFLY
YPEIKDKEEVQRKRQ
>MouseNfkb2
PYLVIVEQPKQRGFRFRYGCEGPSHGGLPGASSEKGRKTYPTVKICNYEGPAKIEVDLVTHSDPPRAHAHSLVGKQC
SELGVCAVSVGPKDMTAQFNNLGVLHVTKKNMMEIMIQKLQRQRLRSKPQGLTEAERRELEQEAKELKKVMDLSIVR
LRFSAFLRASDGSFSLPLKPVISQPIHDSKSPGASNLKISRMDKTAGSVRGGDEVYLLCDKVQKDDIEVRFYEDDEN
GWQAFGDFSPTDVHKQYAIVFRTPPYHKMKIERPVTVFLQLKRKRGGDVSDSKQFTYYPLVEDKEEVQRKRR
>ChickenRel
PYIEIFEQPRQRGMRFRYKCEGRSAGSIPGEHSTDNNKTFPSIQILNYFGKVKIRTTLVTKNEPYKPHPHDLVGKDC
RDGYYEAEFGPERRVLSFQNLGIQCVKKKDLKESISLRISKKINPFNVPEEQLHNIDEYDLNVVRLCFQAFLPDEHG
```

NYTLALPPLISNPIYDNRAPNTAELRICRVNKNCGSVKGGDEIFLLCDKVQKDDIEVRFVLDNWEAKGSFSQADVHR
QVAIVFRTPPFLRDITEPITVKMQLRRPSDQEVSEPMDFRYLPDEKDPYGNKAKRQ
>ChickenRelA
PFVEILEQPKQRGMRFRYKCEGRSAGSIPGEHSTDSARTHPTIRVNHYRGPGRVRVSLVTKDPPHGPHPHELVGRHC
QHGYYEAELSPERCVHSFQNLGIQCVKKRELEAAVAERIRTNNNPFNVPMEERGAEYDLSAVRLCFQVWVNGPGGLC
PLPPVLSQPIYDNRAPSTAELRICRVNRNSGSCQGGDEIFLLCDKVQKEDIEVRFWAEGWEAKGSFAAADVHRQVAI
VFRTPPFRERSLRHPVTVRMELQRPSDRQRSPPLDFRYLPHQGDLQCIEEKRK
>ChickenRelB
PRLIITEQPKKTGMRFRYECEGRSAGSILGESSTEASKTLPAIELLNCQAIPEVQVTACLVWKDWPHRVHPHGLVGK
DCSNGLCQVRLQPHANPRHSFSNLGIQCVKKKEIEAAIEKKLQLGIDPFKAASLKNHQEVDMNVVRICFQASYRDGS
GRTRQLSPVLSEPIFDKKSTNTSELRICRMNKESGPCTGGEELYLLCDKVQKEDIAVVFRKEPWEARADFSQADVHR
QGAIVLRTPPYRCVQLSEPVQVEVFLQRLTDRARSRGCPYTYLPRERDAYGV
KVKRK
>ChickenNfkb1
PYLQIIEQPKQRGFRFRYVCEGPSHGGLPGASSEKNKKSYPQVKICNYVGPAKVIVQLVTNGKYVHLHAHSLVGKFC
EDGVCTVNAGPKDMVVGFANLGILHVTKKKVFETLETRMIDACKKGYNPGLLVHPELGYLQAEGCGDRQLTEREREI
IRQAAVQQTKEMDLSVVRLMFTAFLPDSNGGFTRRLDPVISDAIYDSKAPNASNLKIVRMDRTAGCVTGGEEIYLLC
DKVQKDDIQIRFYEEDENGGMWEGFGDFSPTDVHRQFAIVFKTPKYRDVNITKPASVFVQLRRKSDLETSEPK
FLYYPEIKDKEEVQRKRQ
>ChickenNFkb2
PYLVIIEQPKQRGFRFRYGCEGPSHGGLPGASSEKGHKTYPTVKICNYEGMARIEVDLVTHSDPPRVHAHSLVGKQC
NEAGNCVAIVGPKDMTAQFSNLGVLHVTKKNMMEIMKEKLKKQKTRNTNGLLTEAELREIELEAKELKKVMDLSIVR
LRFTAYLRDSSGNFTLALQPVISDPIHDSKSPGASNLKISRMDKTAGSVRGGDEVYLLCDKVQKDDIEVRFYEDDEN
GWQAFGDFSPTDVHKQYAIVFRTPPYHKPKIDRPVTVFLQLKRKRGGDVSDSKQFTYYPVVEDKEEVERKRK
>FrogRel
PYIEIFEQPRQRGMRYRYKCEGRSAGSILGERSTENNRTYPSIKIMNYTGKGIVRITLVTKNEPHKPHPHDLVGKDC
RDGYYELEFGSDRTVLCFQNLGIQCVRRREVREAIHARIIRKMNPFGVREEQLLTIEDYDLNVVRLCLQVFLPDEHG
NYTRALTPVVSNPIYDNRAPNTAELRICRVNKNCGSVNGGDEIFLLCDKVQKDDIEVRFFTDNWEAKGTFGQADVHR
QVAIVFKTPPFLRSIADAVTVKMQLRRPSDQEVSEPMDFRYLPDPEDPHGNKFKKQ
>FrogRelA
PPVEIIEQPKQRGMRFRYKCEGRSAGSIPGERSTDTSKTHPTIKINNYQGPARIRISLVTKDSPHKPHPHELVGKDC
KDGYYEAELSPDRSIHSFQNLGIQCVKKREVEDAVAHRIRTNNNPFNVSPEELKADYDLNTVCLCFQVFIPDQAAGR
MLPLPFVVSQPIYDNRAPNTAELKICRVNKNSGSCLGGDEIFLLCDKVQKEDIEVIFGLGNWEARGIFSQADVHRQV
AIVFRTPAFQDTKIRQSVKVQMQLRRPSDKEVSEPMEFQYLPDEGDPHHIDEKRK
>FrogRelB
PELNITEQPKQRGMRFRYQCEGRSAGSILGEKSTEHNKTLPEIEIINCDGLEEIHVIVCLVWRDPPHRVHPHGLVGK
DCHNGICEVTLNPQNGVAKHSFSNLGIQCVRKREIDSAVNERLKLNIDPYKAGKWRLHEEVDLNVVRLCFQASCTGP
GFKYDIPPVLSDPIYDKKSTNTSELKISRMNKEYGRCEGGEEVYILCDKVQKEDILVIFGEDKWEARADFSQADVHR
QIAIVLKTPPYHDLHITEPACVRVFLQRITDGIRSEGMPFVYMPRVKDPNGVHSKRK
>FrogNfkb1
PYIEITEQPKQRGFRFRYVCEGPSHGGLPGASSEKNRKSYPQIKIHNYVGQVKVVVQLITNSKDIRLHAHSLVGKNC
EDGICSLTVGPKDTIVGFPNLGILHVTKKKVIEILEARMTDAFKKGHNAALLVHPELNYTNSEDRPLNEREKEIIRQ
AATQQSKDIDLSVVRLMFTAFLLDSEGRFTRSLEPVLSVPIFDSKAPNASNLKIVRMDRTAGCVTGGEEVYLLCDKV
QKDDIQVRFYEEDENGGYWEGFGDFSPTDVHRQFAIVFKTPKYKDVNITKAASVFVQLRRKSDYETSEPKPFLYYPE
IKDKEEVQRKRQ
>FrogNfkb2
AYLSIIEQPKQRGFRFRYVCEGPSHGGLPGASSEKGKKTFPTVKIFNYVGMARIEVDLVTHTDPPRVHAHSLVGKHS
NETGNCIVTVGPEDMTAQFNNLGIVHVTKKSQTEILKEKMKRNILRNTGRNTLTEVEERKIEQEVKDLKKVTDLSIV
RLKFTAYLPDSNGAYTLALPPVISDPIHDSKSPGASNLRISRMDKTAGSVKGGDEVYLLCDKVQKDDIEVQFYEDDE
NGWHAFGDFAPTDVHKQYAIVFRTPPYHTQKIDRPVTVFLQLKRKKGGDVSDSKQFTYYPLEQDKEEVERKRR
>ZebrafishRel
PCVQIFEQPKQRGMRFRYKCEGRSAGSIPGERSSDNNRTYPSIQILNVTGKGKVRVTLVTKSEPYKPHPHDLVGKDC
KDGYYEAEFGPERRAIAFQNLGIQCVRRREVKDAIMQRVTRGINPFNVPREQLLQTEEYDLNVVRLCFQIYLQDESG
MYSTMLPPIVSNPIYDNRAPNTAELRICRVNKNSGSVKGGDEIFLLCDKVQKDDIEVRFFTQTWEAKGSFSQADVHR
QVAIVFKTPAYCDTSITAPVTVRMQLRRPTDQEVSEPMEFRYLPDDKDPYGCREKKR
>ZebrafishRelA

PHVEIIEQPKSRGMRFRYKCEGRSAGSIPGEKSNDTTKTHPAIRVHNYSGPVRVRISLVTKNQPYKPHPHELVGKDC
KHGYYEADLQERRIHSFQNLGIQCVKKKDVGEAVSCRLQTQNNPFKIPDAKIWEEEFDLNAVRLCFQVSITLSSGDL
FPLEPVVSQPIYDNRAPNTAELKICRVNRNSGSCRGGDEIFLLCDKVQKEDIEVRFFLDSWESKGSFSQADVHRQVA
IVFRTPPYCDTNLTEPLRVKMQLRRPSDREVSEPMDFQYLPSDPDEHRLMEKRK
>ZebrafishRelB
PDLVVVEQPKERGMRFRYECEGRSAGSILGASSTDSNKTLPAIEIQGPIDNIKKVMVTVSLVTKDIPYRPHPHCLVG
KDCTDGICVIHINPHSNRRHSFANLGIQCVRRKELDASLQKRRNKNIDPFNTGHSKSIEDMDMNVVRLCFQCELEQK
NGDRITLNPVVSNPIYDKKATTTAELKINRLNVIRGPCTGKTEIYMLCDKVQKDDIEIIFSIEDWEAKAEFAQTDVH
RQIAIVFKSPPFREQDILEETEVNVCLRRMSDRMDSEPVKFTYVPDNADPYGVNRKRK
>ZebrafishNfkb1
PCLQITEQPKQRGFRFRYGCEGPSHGGLPGASSEKNRKSYPQVQICNYQGPARVVVQLVTNSQHPHLHAHSLVGKQC
DKGICISDMQPKDSSISFPNLGILHVTKKNVSKVLEERMMEAYRMGYNYGIFIHPEIDALQGEVRMPRELNEAERSL
ISSAASQQAKEMDLSVVRLMFTAFLPDSDGGFSRRLEPVISEPIYDSKAPNASNLKIVRMDRTAGCVTGGEEVYLLC
DKVQKDDIQVRFYEDDDSGWEAYGDFSPTDVHRQFAIVFKTPKYRDLNLQKPISVFVQLKRKSDNETSEPKPFTYHP
QIIDKEEVQRKRQ
>ZebrafishNfkb2
PYIQIIEEPKQRGFRFRYECEGPSHGGLPGASSERNRRTYPTVKVLNFVGNARVEVQLVTHTDPPRVHAHSLVGRHC
NESGVCSVDVGPSDFTAQFSNLGILHVTKRGVVEVLTKRLKEEKRKVKGPGYKFSDAEENALMQEAKELGKNMDLNI
VRLKFTAYLQDSNGSYTRALKPVVSNPIYDSKSPNASNLKISRMDKTSGSVLGGEEVFLLCDKVQKDDIDIRFYEEE
DEWEALGDFSPTDVHKQYAIVFKTPPYRCTNIDRPVTVFLQLKRKKGGDCSEPKQFTYVPHNQDKEEVQRKRM
>SharkRel
PYVEIAEQPKQRGMRFRYKCEGRSAGSIPGEHSTDNSRTYPSIQIMNYVGRGRVLITLVTKSEPFKPHPHDLVGKDC
REGFYEADFGPDRRVLCFQNLGIQCVRRREVKEAILFRIQRCLNPFNVPQEQLLQIEDYDLNVVRLCFQVFLPDEHG
TFTRALPPVISNPIYDNRAPNTAELKICRINKNTGSVKGGDEIFLLCDKVQKDDIEVRFFTHNWEAKGSFSQADVHR
QVAIVFRTPAYCQTNISEPMTVKMQLRRPSDQEVSEPMEFRYLPDERVNCFHEKRKR
>SharkRelA
PYVRIMEQPKQRGMRFRYKCEGRSAGSILGEKSTDTTRTYPTIEIMNYLGRARIRISLVTKKEPFKPHPHDLVGKDC
KDGYYEADLQERSVHSFQNLGIQCVKKREVSLAIQQRLMKDVNPFNISPEVLANENEYDLNVVKLCFQVFIPDPSGR
CTVPLTPVVSNPIYDNRAPNTAELKICRINKNSGSCRGGDEIFLLCDKVQKEDIEVRFFTSDWEGRGSFSQADVHRQ
VAIVFRTPAYRDLSLERPQLVHLQLRRPSDKEVSDTMEFQYLPHDKDPYSKEEKRR
>SharkRelB
PKLVITEQPKQRGMRFRYECEGRSAGSIPGENTNEHNKTLPTVQIQNWQGEVRIVVSLVTKDRPFKPHPHSLVGKDC
QNGICEVTVSPKCNMKASFSNLGIQCVKKKEVLKAIELRQKLGIDPYNVGDVARCIDDIDMNVVRLCFQAHIVKPHI
NLEPVLSDPIYDKKATNTSELRICRLNKDCGSCTGGEELFLLCDKVQKEDIAIVFSTKNWEAKGVFSQSDVHRQIAI
VFKTPPYCDIEIEEQASVELRLYRPSDKEYSDPFIFRYQPKQI
>SharkNfkb1
PYLQIVEQPKQRGFRFRYGCEGPSHGGLPGASSEKNKKTYPTVKICNYVGNAKIVVQLVTCGKTAVHLHAHSLVGKQ
CEKGICLVQLGPKEMTSVFPNLGILHVTKKNVASTLEQRMVHACIMGYNNGIVIHPEINHRHIETCSMRELTERERE
LIHQASIQQAKEMDLSVVRLMFTALLPDSSCRFTRRLEPVISDPIYDSKAPNASNLRIVRMDKTAGCVTGGDEVYLL
CDKVQKDDIQVRFYEENENGGIWEALGDFGPTDVHRQFAIVFRTPKYRDLTISKPASVFVQLRRRSDNETSEPKPFT
YYPQINDKEEVQRKRQ
>SharkNfkb2
PSLIITEQPKQRGFRFRYGCEGPSHGGVPGASSEKNRKTYPSVKIINYCGNAKIVVSLVTNNKPHMCHAHSLVGKHC
TEEGTCVVSVGPKDMTAQFANLGILHVTKRNVPKVLLRRFLGPTESDPAATKTPVRKNSQDEKLQQLKKESENLAKT
MDLSVVRLQFTAYLPDSTGLYTLELKPVISDPIFDSKAPNASNLRIVRMDKTFGCVTGGEEIYLLCDKVQKEDIQVR
FYEGNAESDWEAFGEFGPTDVHRQFAIVFRTPCYRDTNIRKPVSVYVQLRRRLQVECSEPTAFTYCPKFKEREEIYR
RKK
>SharkNfkb3
PYIEIIEQPRQRGFRFRYGCEGPSHGGLPGVSSERSKKTYPTVKIHNYTGPAKIVVQLVTIDEPALLHVHSLVGRQC
GGGTCVVRIDSEDMTASFPNLGILHVPKKEVATIIEDQLVRAWSLESQDGQESPQDRNSGNPTASSSQKQLRNEDRE
KIHINAQKQAKCMDLSVVRLMFTPYLLGNDDKLMCRLSPAISDPIYDSKAPNASSLRIVRMARTAGSVVGGDEVFLL
CDKVQKDDVQVRFYEEDGNGQMWEAFGKFSPTDVHRQFAIVFRTPKYYDVHIAKPVSVFVQLRRRSDGESSESTPFI
YYPSTQDKDPIQKKWQ
>SkateRel
PYAEMVEQPKPRGMRFRYKCEGRSAGSIPGANTKDNNRTYPAIQVVNYFGRVRVLVTLVTKSEPFKPHPHDLVGKDC
KDGSYEAEFGAERRILCFQNLGIQCVKRKEVKDAILFRIQRQLNPFNVPQEELLQIASYDLNVVSLCFQVFLPDEHG

SFTRALPPVVSNPIFDNRAPNTAELKICRVNKNTGTVKGGDEVFLLCDKVQKDDIEVRFFTTNWEAKGSFSQADVHR
QVAIVFRTPAYCQTNITEPVSVKMQLRRPSDQEVSQPMEFRYLPDERDNYFSEKRKR
>SkateRelB
PRLVITEQPKQRGMRFRYECEGRSAGSIPGENTTEINKTLPTAQIQNWHGEVKMVISLVTKDTPYKPHPHSLVGKDC
QNGICEITVSPKCNMKASFANLGIQCVKKKETTRAVEHRLKIGVDPFNVGDSLNYLEDIDMNVVRLCFQAFIKDPYI
ALTPVLSEPIYDKKATNTSELRICRLNKDCGVCTGGEELFLLCDKVQKEDITVVFSGDSWEARGVFSQTDVHRQIAI
VFKTPPYSDIDIQQPVSVSLRLYRPSDKEYSDPFEFRYTPKDIDYYGIHQKWK
>SkateNfkb1
PYLQIIEQPKQRGFRFRYGCEGPSHGGLPGASSEKNKKAYPAVKICNYVGHAKIVVQLVSGGGDQAHLHAHSLVGKQ
CERGICVVQAGPKDMLSTFPSLGILHVTKKNVASTLEQRMSQALLQGYNAGVLIHPDLDVLHADTCPPAELTTCQRE
LIRGAAVLQAKRMDLSVVRLMFTALLPDSGCRFTRRLIPVISQPIYDSKAPNASNLRIVRMDRTAGCVTGGDEVYLL
CDKVQKDDIQVRFYEEDENGGMWEAFGNFGPTDVHRQFAIVFKTPKYRDVEISKPASVFVQLRRKSDCETSEPKPFT
YYPLIRDKEEVQRKRL
>SkateNfkb2
PFIDICEQPKQRGFRFRYGCEGPSHGGLPGASSEKNRKTYPSVKIYNYTGNAKVLVQLVTAKEPPRLHAHSLVGKQC
TDEGICIVHVGPKDMMAQFTNLGILHVTKKNVPEVLQDRIFNELYQKNPLSSQDFLSKSLLSGQELQSLKQESEQLA
KEMDLSVVRLRFTAFLPDSQGMYTLPLPPVISDPIFDSKAPNASNLKIVRMDKTSGCVMGGDEIYLLCDKVQKDDIQ
IRFYEDDERAWEAYGDFGPTDVHRQFAIVFRTPRYWDISIERPITVFVQLKRRKDGETSEAKAFTYCPRVEDKEEVN
RKRQ
>SkateNfkb3
PYIEIIEEPKQRGFRFRYGCEGPSHGGLPGVSSEKNNKTYPAVKIHNYTGPARVIVQLVTSDEPPLLHVHSLVGRQC
KNGICFAEIDSEDMTASFPNLGILHVPKKDVAAIIEEQLVKSWMSDLNDENPSLSDEDGVELQQNQLQKEDRERIHH
EAQKQTKYMDLSVVRLMFTPYLPGSDGKFTHRLSSVISGPIYDSKAPNASSLRIVRMAKTAGSVVGGEEVFLLCDKV
QKDDIQVRFFQEDESGHIWDAFGKFSAADVHRQFAIVFKTPKYFDMHITKPISVFVQLRRRSDGETSEPKPFIYYPH
KQAKKKIEKKWQ
>LampreyRel1
PQLQIVQQPRQRGMRFRYECEGRSAGSIPGEHSTDSNRTFPAVQILNYKNRAKIRVSLVTKSDPPKPHPHSLVGRDC
RDGICEMDIGPDSMIASFSNLGIQCVRRREIMDALQLRLKKKVDPFNVGNAALDIEDMDLNVVRLCFEAFVYDSAQN
VVALPPVVSHEIRDRRATSTSELKICRVSQNVGSVRGGDEIFLLCDKVQREDIEVRFFDDSGWEARGLFSQTDVHRQ
VAIVFTTPPYRDQAVTREVTVRMQLRRPTDNEVSDSMEFRYLPYDYDPYGIKVKRK
>LampreyRel2
PQLEVYEQPKSRGMRFRYECEGRCAGSILGDRSTDSNKTYPAVKLVNCTGPAKVRVSLVSKNDPHRPHPHSLVGKDC
SDGVCEVDVSSGVTIVQFQNLGIQCVKKKEVADALKLRLQKNVNPYKVSEEQALGTEEIDMNVVRLCFEAFIHERGR
VIALPPIVSQEIRDKKAPNVSELKICRVNLNAGSARGGDEVFLLCDKVQKEDIEVRFFEPDGSWESRGSFSQADVHR
QVAIVFRTPAYRDPGITRPASVRMQLRRPSDGEASEPIEFRYIPVDPDPHKLQEKR
>LampreyRel3
PALVIVEEPKQRGMRFRYQCEGRATGSIFGERSDTSTKTYPAVQVQNYSERVLLRVSLVSKEEPYRPHPHALVGTDC
NDGIFQATLEPPDLRVQFQNLGIQCAKRKDIMSAIRMRVTKQKIDPFNVGALAFEMEGLDLNAVRFCFEAFLINSHG
SIVKALPPVVSNPIYDKKGCNTSELKIIRLNEHSGCAAGGDERYILCDKVQKEDIAVRFFDEDAWEAQGVFSQTDVH
RQVAIVFRTPPYRDGHTAVPVCVRVQLLRPSDGETSEPLEFHYTPVDTDPHHLQQKRK
>LampreyRel4
PFLEVVEQPEARGMRFRYPSEGACAGSLLGASTTDRDKTYPSVRLVNLTGKAEVQVCLVTRDAPHRPHPYSLVGRDC
EDGIFKQLVVQGCSTVQLINVGIQCVKKSMVQRSAEDKIRKINPFKVSDRVVVRPEEMDLTAVRLCFQAKLLPSNQI
LTPVLSEIIFDKKATCASQLKITRMNKTYGSVHGGDEVMLFCEKVQKDDIRVVFSLAGWQAEGIFSTGDVHKQVAIV
FKTPAFHDQSICAPVKVSIHLWRPSDQQQSIPHSFLYKPELDDAFGVERKRK
>LampreyNfkb
PYLQITEEPKQRGFRFRYPCEGPSHGGLPGCSSERNKKTFPTVRICKYFGLAKVVVQLVTENAPYRLHPHSLVGK
NAENGAVSCTVGPHDMTAMFPSLGVLHVTKKSVASVLEKLWSDAQVSGLASIGNIVLENSFNNVNARALTDAEARQI
KNDAQQGAKTIDLSVVRLMFTAYLPDSNGIFTHRLPPVFSNPIYDSKAPNANNLKIVRMDKTSGCVTGGDEVYLLCD
KVQKEDIQVRFFHDDWEAWGNFGPTDVHKQYAIVFKTPRYCNVDISKSVTVFVQLRRKSDSETSEAKPFIYYPQKQD
KEEVARKRQ
>HagfishRel1
PCLEILEQPKQRACDSANECEGRSAGSIPGERSTDTNRTYPAVQIQNYKGPAKIRVSLVTKSNPPRPHPHSLVGREC
KDGICEMELLPGNSTSFPNLGIQCVRRREILDSLRLRLQRNVDPFQVGNAALNLEDIDLNVVRLCFEAFITDPTGNM
AALPPIVTQEIRDRRATSTSELKICRVSRSVGSVQGGDEVFLLCDKVQRGECFSFFFSIDNWEAKGSFSQTDVHRQV
AIVFCTPPFHDQNIYNEMTVRMQLRRPSDGEVSESVDFRYLPRDRDPFGIGIKRQ
>HagfishRel2

PFLEIIEQPKRRAMRFRYECEGRSAGSILGERSTDSFKSYPAVQVVNGIGPARLRVSLVTKSEPYHPHPHALVGKDC
KDGVCELEIPRAATITQCVNLGIQCVKKKEVLASLLQRLRNGINPFVLESELLGVDETDMNVVRLCFEAFVRDGQIW
RPLAPVVSQDIRDKEATNVSELKICRVNKNISQANGGEEIFILCDKVQKDDIEVRFFNESGWEAFGCFAQTDVHRQV
AIVLRTPPYCDPAITTTPATAFMQLRRPSDGEASDPLDFQFIPSD
>HagfishRel3
PRLEILEQPRQRGQRFRYECEGRSAGSILGENSTEQHKTYTAVQVNYAGRVRLVMSLLTHEPPFRPHPHSLVGKNCE
DGVCILELDPPNLYVQFQNLGIQCVKRKHVFDAIKQRLIRRFVVGGRALKMENLHMNAVRLCWQAYLLDNHGNVLRA
LPPVLSNPIYDKEGTNTSELRISRLNINHGSVAGGDECFILCDKVQKEDIEVVFSHGSWTELADLSQTDVHRQVAIV
FRTPPFYQRDITAPVHVRMQLRRPSDRVVSEAVSFFYTPLDSGEFG
>HagfishRel4
MLVVFVEQPQPRGMRFRYPCEGHSAGSLFGENSTEANRTYPTLQVLFNSLPVELMICLVTADQPYKPHPHNLVGKNC
RDGIYRSSLRFSSVGIQCCKKNDVRNIILKRLSDGIDPFLQTESINLNSVRLCVQPVLLYSYGRQVMPPTVSDIIYD
KAENTCASILKIIRFNKIAGSVLGDDEVILLCDKVQKDNIKVRFFLDKWEAWGIFSANDVHRQIAITFKTPPYKDPC
ISSPVEVYLQLFRPSDRVTSEAKPYSFIPQCKGKLNIKTK
>HagfishNfkb
KLGTDPDEILWHGFRFRYSCEGQSHGGLPGKTSGQTRKTYPAVKVINNYTGKAKVRVQLVTNTDPPLLHAHNLVGKK
VESGSITMDVGPHDMTAV**L**YGIGIEHVKRKVHPSVLEERWVFNKVRDQYSNMDISIKDAHVYLSVFELQQIKNKVQE
EVKKINLNVVRLMFTALLPDNHGLFTRRLPSVYSLPIYDAEAPSSTVLQIIRMDRSSGSVIGGEEIYLLCDKVQKDD
IKIRFFDINGWEGFANFGPIDVHRQVAIVFKTPAYSNSEITSPVRVDVELVRQSDSMTSDPKHFTYYPKAEDKKEVA
RKRR
>CionaRel
PVLEIVEQPKQRGMRFRYECEGRSAGSIPGKNTNGDRKTWPSCQVLNYSGVAIMRVSLVSKDDPPRPHPHSLVGRDC
NNGVCQINVDPGNQMLGVFPNLGIQCVRRREVGQAIQDRLNHGVNPFGTMLDGDERSAVDVDLNIVRLCFEAFIPDA
RGKYTQKLEPVVSDPIYDKKATCSSVLKICRVDKTHGSCMGNEEVFLLCDKVQKEDIQVVFYRDNWEALGDFSSVDV
HRQVAIVFRTPPFCNENIQEKVDVQFKLRRPSDMETSKPLVFTYLPVYHEMPQKHKFPT
>CionaNfkb
PYLEIIENPKSRGFRFRYTCEGPSHGGIPGGSSDKNKKTFPAVKICNYQGYARIVVQLVTNEENPRLHPHSLVGKQC
QNGICTVQCGPKDMTATFPNLGIQHVTKKNVATILEERYIAAEMQLSSINDGFPQEVQRNIKDEDRKRIAAKAQSEA
KSIDLSVVRLMFIAYLPDSNGAFTIMLKPVISDAIFDSKAPNAATLKICRMDCNAGSASGGDEVYLLCDKVQKDDIQ
VVFSEEDMQGNNLWEAYGSFSPTDVHRQFAIVFRTPAYKDTEIKMPVNVQVQLRRKSDNEVSESRPFTYLPNKSDLE
LIDRKRR
>SeaPineappleRel
PALVITEQPKQRGMRFRYECEGRSAGSIPGENTTQEKKTWPTVQIQNFRGDVMIRVSLVSKDSPPKPHPHSLVGKDC
ENGICSVRVSPETQMTACFSNLGIQCVKRKEVTEALMERRRLTVDPFKTVVDGDERPNVDIDLNIVRLCFEAFCFTA
NGRLPLQPVVSNPIFDKKSTSSSLLRICRVDKSFGSCKGGDEVYLLCDKVQKDDISVCFFDLNTGWESYGEFSPTDV
HRQVAIVFRTPPYENTHIREATKVMFQLKRSSDGETSDSKDFTYLPLDHEQPQINIPRA
>LanceletRel
PVIEIIEQPKARSLRFRYECEGRSAGSILGENSSPENRTYPSIRLLNCSGPAMILVSLVTKDDPPKPHPHSLVGKGC
IHGICKINVPDCRAPISFPNLGIQCVKRKEITQALAQRLRLGIDPFHTYNRHKGKMDEVDLNTVRLCFQAFLPDPQT
GQCTVSVHPRVSTPIHDKKAPGAAELRICKMNKVSGPVTGGDEVTLLCDKVQRDDIEVIFTAKPMGIKWESRGDFSP
TEVHRQVAIVFKAPAYFNLTIREPVRVQVRLRRPSDGEESEPFDWMYTPVDKDEHSVESKRR
>SeaUrchinRel
PYCEITEQPKQRDHRFRYPVEGRQAGSIAGEKSTSDLPSYPTIKVANLSGRAKVVVSLVTKNDPPLPHPHRLVGDGC
RDGVCTRMIDPQRPEVVFHKIGVQRTMNKEVENSLEERKRAGVKLSMVVNKGKTGKKHNYEMKAVRLFFEVYIETIE
SSGVFDKYLTPVTSTAVYDKKDTVLSICRVNISTGSVEGGDELFILCEKVQSDDIKVKFYGHDAEKNQPWEAFGEFS
PSDVHRQFAIVCKTPRFVNQNIKTAVTVQFHLYRPSDDESSREMPFIYKPRESVHGFRDIEKK
>SeaUrchinNfkb
PHLKILEQPRQRGFRFRYGCEGPSHGGLPGQNSQRGKRSFPSVEICNYKGSARIVVSLVTNEETPRPHAHSLVGKHC
KDGLCTVQVGPKDMTASFPNLGILHVTRKDVVPTLKTRILAQHRLYKDLINNSTPGESHWTEPSDAEIEKKAKEMAK
DMDLSVVRLCFQTYLPDISGHFTRPLDPVISVPVFDSKAPNATTLKICRMDKSAGCCTGGEEVYLLCDKVQKEDIQV
KFFEISADGQMVWQSLAEFGPTDVHRQYAIVFKTPAYKDINIDKPVYVHVQLKRKSDNETSDPKPFTFHPQVPDREG
ILRKRK
>DrosophilaDorsal
PYVKITEQPAGKALRFRYECEGRSAGSIPGVNSTPENKTYPTIEIVGYKGRAVVVVSCVTKDTPYRPHPHNLVGKEG
CKKGVCTLEINSETMRAVFSNLGIQCVKKKDIEAALKAREEIRVDPFKTGFSHRFQPSSIDLNSVRLCFQVFMESEQ
KGRFTSPLPPVVSEPIFDKKAMSDLVICRLCSCSATVFGNTQIILLCEKVAKEDISVRFFEEKNGQSVWEAFGDFQH
TDVHKQTAITFKTPRYHTLDITEPAKVFIQLRRPSDGVTSEALPFEYVPMDSGKHTFWNLHRH

```
>DrosophilaDif
PHLRIVEEPTSNIIRFRYKCEGRTAGSIPGMNSSSETGKTFPTIEVCNYDGPVIIVVSCVTSDEPFRQHPHWLVSKE
EADACKSGIYQKKLPPEERRLVLQKVGIQCAKKLEMRDSLVERERRNIDPFNAKFDHKDQIDKINRYELRLCYQAFI
TVGNSKVPLDPIVSSPIYGKSSELTITRLCSCAATANGGDEIIMLCEKIAKDDIEVRFYETDKDGRETWFANAEFQP
TDVFKQMAIAFKTPRYRNTEITQSVNVELKLVRPSDGATSAPLPFEYYPNPELLTKHNRRVA
>DrosophilaRelish
PQLRIVEQPVEKFRFRYKSEMHGTHGSLNGANSKRTPKTFPEVTLCNYDGPAVIRCSLFQTNLDSPHSHQLVVRKDD
RDVCDPHDLHVSKERGYVAQFINMGIIHTAKKYIFEELCKKKQDRLVFQMNRRELSHKQLQELHQETEREAKDMNLN
QVRLCFEAFKIEDNGAWVPLAPPVYSNAINNRKSAQTGELRIVRLSKPTGGVMGNDELILLVEKVSKKNIKVRFFEE
DEDGETVWEAYAKFRESDVHHQYAIVCQTPPYKDKDVDREVNVYIELIRPSDDERSFPALPFRYKPRSVIVSRKRRR
TGS
```

Supplementary Table 3. NF-κB motifs identified by PBM[28] at NF-κB ChIP-seq peaks near the promoters of *Rel*-dependent genes. Focusing on Rel ChIP-seq peaks between -1500 and +500 relative to the TSS of the Rel-dependent *Il12b*, *Tnfsf9*, *Noct*, *Il4i1*, and *Clcf1* genes, motifs with NF-κB z scores greater than 4 (defined by Siggers et al. 2011,[28] http://thebrain.bwh.harvard.edu/nfkb/index.php) are shaded. The DNA sequences 100 bp upstream and 100 bp downstream of each ChIP-seq peak summit are shown, with z scores provided for three mouse NF-κB dimers: Rel:Rel, RelA:RelA, and RelA:p50. Also shown are the peak number, the location of the peak summit (underlined), and the Rel/RelA ChIP-seq RPKM ratio. As controls at the beginning of the following analysis, PBM Z scores and Rel/RelA ChIP-seq RPKM ratios are shown for consensus and near-consensus motifs at three inducible, *Rel*-independent promoters.

Control consensus and near-consensus motifs at *Rel*-independent promoters:

Nfkbiz promoter: GGAAATTCCC
Rel/RelA ChIP-seq RPKM ratio: 1.5
PBM Z scores: Rel:Rel 15.8; RelA:RelA 12.2; RelA:p50 9.2

Tlr2 promoter: GGGAATTCCC
Rel/RelA ChIP-seq RPKM ratio: 1.7
PBM Z scores: Rel:Rel 17.7; RelA:RelA 13.2; RelA:p50 11.5

Ccl5 promoter: GGGAGTTTCC
Rel/RelA ChIP-seq RPKM ratio: 1.9
PBM Z scores: Rel:Rel 11.7; RelA:RelA 7.2; RelA:p50 9.5

ChIP-seq peak regions near *Re*l-dependent genes

Il12b promoter, Peak 10094
ChIP-seq peak summit relative to annotated mRNA (red): -185 bp
Rel/RelA ChIP-seq RPKM ratio: 3.0
Three PBM Z scores are shown for shaded motifs: Rel:Rel/RelA:RelA/RelA:p50
Note that the κB1, κB3, and κB4 motifs do not have Z scores because these sequences were too divergent from the consensus to be included on the PBM arrays.

CTTAGGCATGATGTAAACAGAAATTAGTATCTCTGCCTCCTTCCTTTTTCCACACCCCGAAGTCATT

6.0/<4/<4
TCCTCTTAACCTGGGATTTCGACGTCTATATT**C**CCTCTGTATGATAGATGCACTCAGGGAGGCAAGG

12.8/8.7/8.3
GGGGGAGGGAGGAACTTCTTAAAATTCCCCCAGAATGTTTTGACACTAGTTTTCAGTGTTGCAATT
κB1 κB2 κB3 (κB4)

Il12b slightly upstream peak, Peak 10093
ChIP-seq peak summit relative to annotated mRNA (red): -1,041 bp
Rel/RelA ChIP-seq RPKM ratio: 2.2
Three PBM Z scores are shown for highlighted motifs: Rel:Rel/RelA:RelA/RelA:p50

<4/<4/4.1
GTGAGGAAGTGTGTGGCTGGGAAGCTCCGCAGTTCTCTCCCCCTCCCCGCGCCCCAGGCAGACTGAC

ACTTGGAAGAAAGTTAGGCCGGAAATCTACAC**C**GCCCGAAACCTGACAGGGTCCAACCTCAGCCCTA

11.5/7.6/5.5
GGAACTTCCCAGGAGGCGGGAACCCAGGCCAGGGACAGGAATGGAGAAGCGGCGGGTTGTGTCCCT

Tnfsf9 promoter, Peak 30385
ChIP-seq peak summit relative to annotated mRNA (red): -108 bp
Rel/RelA ChIP-seq RPKM ratio: 2.3
Three PBM Z scores are shown for highlighted motifs: Rel:Rel/RelA:RelA/RelA:p50

ACACACACACACACACACACACACAAAAAGGGAAGAAGGACGGGCGGGCACAGGGCCTGGACAGGGA

AGGGGAAAGGGGCGAGAAGGGGGAGGGGAAGG**G**GGGAGGAGGAGAGAAAGTTCCGGGAGTCGAGGGG

7.5/<4/<4
CGGAGCGCGGAGCCCGGGAACCCCGCGGCTGCCCTCCCTCCCTTCCCTCCCCGGAAAAGGCGCCTC

Tnfsf9 slightly upstream peak, Peak 30384
ChIP-seq peak summit relative to annotated mRNA (red): -745 bp
Rel/RelA ChIP-seq RPKM ratio: 3.2
Three PBM Z scores are shown for highlighted motifs: Rel:Rel/RelA:RelA/RelA:p50

8.3/6.9/4.0
GCCCCCCCCTTTTGCCTCTACCTCAGGCAAGAACAGTGAAAGAATGGGAAATTGGAAATGTGTCCGC

11.1/7.1/7.0
TGCCTGGGGGAATTTCTCTATCTGGACTCTCC**A**GCCTTCTGTTTCTGGAGTCCTAAGAGACTACTAA

CAGGAAGCCCCAAGAAAGTGGGTGGGGACACACCAGGAAAGGCCCAGGGTAGAGATGGGGTCCTGG

Noct promoter, Peak 42411

ChIP-seq peak summit relative to annotated mRNA (red): 193 bp

Rel/RelA ChIP-seq RPKM ratio: 2.3

Three PBM Z scores are shown for highlighted motifs: Rel:Rel/RelA:RelA/RelA:p50

CCTGGTGCCTAATCTCCTTCAGCACCGCGGAGCGTGGAGCCCACAGCTCGGGGAGGACGTTCTCCCA

11.0/6.2/<4

GACACGCGCTCGCACACACTCAGCGCTGGCAC**C**AACCCGGGATTCCCAGCCTGTGGACGCTCCCATT

GCAGCCGGCGGGAGGGGACAGAGGCTCCCGGAACGCCTCTCTAACGAATCCCCGCGGTGTCAGCCA

Noct slightly upstream peak, Peak 42410

ChIP-seq peak summit relative to annotated mRNA (red): -650 bp

Rel/RelA ChIP-seq RPKM ratio: 2.7

Three PBM Z scores are shown for highlighted motifs: Rel:Rel/RelA:RelA/RelA:p50

15.8/12.2/9.2

GTGTGTGCATCTGACTCTCAGACTATTGAAACAATGGACCCCCCAAGAAATGGGAATTTCCTTCA

10.7/5.1/<4 8.2/7.0/7.2 8.3/7.1/5.1

GGAAACTCCTGCTGGGAAAAGGAAAAGGCCTTTG**C**TTGAGGGGGAATTCTTTCAGGAAGCGGGAATTCAC

ACAGTTCTATCTAGTCAGATCGCTTTGACCCGGACTGCTGAGATGTGCATACCCCTGGCCTTCTA

Il4i1 slightly upstream peak, Peak 58509

ChIP-seq peak summit relative to annotated mRNA (red): -622 bp

Rel/RelA ChIP-seq RPKM ratio: 2.0

Three PBM Z scores are shown for highlighted motifs: Rel:Rel/RelA:RelA/RelA:p50

8.8/4.9/7.1 5.0/6.1/4.5

CATCTACTTAATGGGCTCTGGAGTTCCCCAGCCTGCGCTCTCTGTGATTTCCCACCCTAGCTGTTCT

10.9/7.1/8.4

CCTAACCCATCTTCTCCCCAACCCCAAGGAAA**A**CCCCCTGCATCCTGTTGCCTCACCTCACCAGTTT

TGGCAGAGGCTTCTAGGGTCATTTCTCTGAGGTGTCAGTCTAGAGTCTGCTGCCCAGCAGTACTTA

Clcf1 promoter, Peak 33686
ChIP-seq peak summit relative to annotated mRNA (red): -18 bp
Rel/RelA ChIP-seq RPKM ratio: 2.1
No motifs with PBM Z scores above 4 for any NF-κB dimers

TGCTACAATTTGTGGTTACAGCTTTACACGTCAGAAAAAAAAAAGTTTGCAGAATGTCCCACACCGA

AAAAAAAATGCGAAACCGAGAGAAAAAAACCT**G**CGAGTGGGCCTGGCGGATGGGATTATTAAAGCTT

CGCCGGAGCCGCGGCTCGCCCTCCCACTCCGCCAGCCTCTGGGAGAGGAGCCGCGCCCGGCCGGCC

Clcf1 slightly upstream peak, Peak 33685
ChIP-seq peak summit relative to annotated mRNA (red): -720 bp
Rel/RelA ChIP-seq RPKM ratio: 1.9
Three PBM Z scores are shown for highlighted motifs: Rel:Rel/RelA:RelA/RelA:p50

7.0/<4/<4
AGAATGCTCTTAGCCAAACCACCCCTACCTCACCACCGGAAAAACCAACAACCCAAACCCCCAACAA

AGGGAAGAGGATGCCAAATGTCTCGATGGGGA**A**GCTTTTCACTAACGGGATGCTGGGTCTATCCATG

GCCTTGAATCTTCGAGTTCCCGCTGAGTCACCCGTGAAGCCTCTGGTGTTGAAACTGGTCCGGTGC

Supplementary Table 4. NF-κB motif scores vs. Rel/RelA ChIP-seq RPKM ratios at primary response promoters from Tong et al. 2016[38] (see also Fig. 4). We previously identified promoters of primary response genes that were strongly induced by lipid A in BMDMs and that strongly bound RelA.[38] We divided these promoters into two groups: one group contained consensus or near-consensus NF-κB RelA:p50 motifs as defined by PBM z score, and the other contained divergent motifs. In this table, we examined the relationship between motif score and Rel/RelA ChIP-seq RPKM ratio at these peaks. We calculated the Rel/RelA ChIP-seq RPKM ratios at each of the promoters to determine whether a relationship existed between PBM z score and Rel/RelA ChIP-seq RPKM ratio. This analysis was first performed using PBM z scores for Rel:Rel homodimers, and was then repeated with z scores for RelA:p50 heterodimers and RelA:RelA homodimers. The results demonstrate that RelA and Rel ChIP-seq peaks have, with high statistical significance, larger Rel/RelA ChIP-seq ratios at promoters with weaker motifs than at promoters with strong consensus and near-consensus motifs, despite the fact that Rel homodimers bind both consensus and non-consensus motifs with higher affinity than RelA homodimers. This correlation was the strongest (p<0.001) when peaks were classified on the basis of their RelA:p50 PBM z scores. Notably, only one of these primary response promoters (*Tnfsf9*) is associated with a gene that exhibits Rel-dependent transcription in lipid A-stimulated BMDMs (Fig. 1). This finding demonstrates that a high Rel/RelA ChIP-seq peak ratio, although strongly correlated with Rel-dependent transcription, is not sufficient for Rel-dependence.

For all tables below, the gene list is from Tong et al. (2016);[38] the motif shown for each promoter is the motif within a 200-bp region at each ChIP-seq peak containing the largest NF-κB motif z-score as defined by Siggers et al. (2011);[28] peak numbers are from the ChIP-seq datasets used for the current study; motif scores for mouse RelA:p50 heterodimers and Rel:Rel and RelA:RelA homodimers are from the NF-κB 10-bp motif tool described by Siggers et al. (2011;[28] http://thebrain.bwh.harvard.edu/nfkb/index.php). Rel/RelA ChIP-seq RPKM ratios were calculated using the average ChIP-seq scores from the current study.

SORTED BY Rel:Rel MOTIF Z SCORES

```
p-value between Datasets 1 and 2 below: 0.004
Ratios 1.80 or less:         Dataset 1, n=25,      Dataset 2, n=6
Ratios 2.00 or greater:      Dataset 1, n=4,       Dataset 2, n=14
Ratios between 1.81 and 1.99: Dataset 1, n=11,     Dataset 2, n=5
```

Dataset 1: Promoters with Rel:Rel motif scores >9.0 (n=40)

Gene	Motif	Peak#	RelA: p50	Rel: Rel	RelA: RelA	Rel/RelA Ratio
Cxcl2	GGGAAATTCC	51430	10.3	18.2	13.9	1.69
Cxcl1	GGGAAATTCC	51426	10.3	18.2	13.9	1.68
Cxcl10	GGGAAATTCC	51487	10.3	18.2	13.9	1.97
Ifn1	GGGAAATTCC	47146	10.3	18.2	13.9	1.91
Ier3	TGGAATTTCC	29729	7.9	18.0	13.0	1.75
Acod1(Irg)	TGGAATTTCC	22405	7.9	18.0	13.0	1.56
Bcl2l11	GGGAATTCCC	39793	11.5	17.7	13.2	1.39
Icam1	TGGAAATTCC	64896	7.2	17.0	13.2	1.78
Nfkb1	GGGAATTTCC	44851	9.2	15.8	12.2	1.39
Nfkbia	GGGAATTTCC	14813	9.2	15.8	12.2	1.80
Nfkbiz	GGGAATTTCC	27524	9.2	15.8	12.2	1.46
Fchsd2	GGGAATTTCC	59885	9.2	15.8	12.2	1.64
Gbp5	GGGAATTTCC	45059	9.2	15.8	12.2	2.06
Tlr2	GGGGAATTCC	43113	12.2	14.8	11.6	1.72
Nkbib	GGGGAATTCC	58154	12.2	14.8	11.6	1.38
Sdc4	GGGGAATTCC	41035	12.2	14.8	11.6	1.52
Relb	GGGGAATTCC	57900	12.2	14.8	11.6	1.83
Tnip3	GGGATTTCCC	55196	7.2	12.9	8.4	2.27
Cd40	GGGATTTCCC	41079	7.2	12.9	8.4	1.82
Ptgs2	GGGGATTCCC	03319	9.1	12.7	7.7	1.58
Ier2	GGGTTTTCCC	63225	5.7	12.6	7.6	1.91

Cd44	GGGTTTTCCC	38999	5.7	12.6	7.6	1.89
Rasgef1b	GGGTTTTCCC	51638	5.7	12.6	7.6	1.87
Nfkb2	GGGACTTTCC	35430	8.4	12.4	8.5	1.77
Csf1	GGGACTTTCC	44096	8.4	12.4	8.5	1.59
Btg2	GGGAAAGTCC	02911	6.7	12.2	8.2	2.07
Ccrl2	GGGAAAGTCC	67639	6.7	12.2	8.2	1.76
Nfkbid	GGGGAATTCC	58246	12.2	11.8	11.6	1.79
Ccl5	GGGAGTTTCC	11826	6.8	11.7	7.2	1.89
Tnfaip3	GGGGATTTCC	05530	10.0	11.4	9.2	1.71
Casp4	GGGGATTTCC	64595	10.0	11.4	9.2	1.78
Rel	GGGGATTTCC	09682	10.0	11.4	9.2	1.80
Ebi3	GGGGGTTTCC	30307	10.6	11.2	8.2	1.79
Nfkbie	GGGGGATTCC	29942	8.6	11.0	6.9	2.07
Slfn2	GGGATTCCCA	11801	<4.0	11.0	6.2	1.96
Stx11	GGGGTTTTCC	05245	8.4	10.9	7.1	1.94
Clec4e	GGAAATTCTG	56755	<4.0	10.1	5.7	1.58
Cd83	GGGACTTCCC	17889	6.4	9.6	5.6	1.56
Bcl3	GGGACTTCCC	57917	6.4	9.6	5.6	1.83
Irf1	GGGGAATCCC	10386	7.0	9.4	6.2	1.69

n=40
Mean Rel/RelA ratio=1.76
Standard Deviation=0.1964

Dataset 2: Promoters with Rel:Rel motif scores <9.0 (n=25)

**Gene**	**Motif**	**Peak#**	**RelA: p50**	**Rel: Rel**	**RelA: RelA**	**Rel/RelA Ratio**
Slc2a6	GGGTTTCCCC	36646	5.2	8.9	5.8	2.60
Il1rn	TGGGGAAATT	36501	5.6	8.3	6.9	2.06
Dusp5	GGGTATTTCC	35632	5.7	8.2	5.5	2.24
Cflar	TGGGGTTTTC	01044	4.6	7.9	4.8	2.01
Rapgef2	AGGGGGTTTT	43012	8.0	7.5	5.3	1.59
Traf1	AGGGGATTTT	37086	7.1	7.4	5.3	1.83
Cxcl16	CGGGGATTTC	11135	5.8	7.1	6.8	2.23
Ccl3	GGGAAAATTT	11848	4.9	6.8	7.0	1.77
Kdm6b	GGGGGATTTC	11036	7.4	6.6	5.4	1.59
Gpr84	AGGGGGAATT	25702	6.8	5.9	5.9	1.58
Rela	AGGGGATTTC	33773	6.5	5.2	4.9	1.40
Tfec	TGGGGAAAAA	53859	6.0	4.7	6.1	2.09
Srgn	TGGGGAAAAC	06824	5.2	4.6	6.1	2.03
Zhx2	AGGGGGAAAA	23954	6.1	4.5	6.1	2.07
Gm6377	GGGTGGGAAA	68997	4.5	4.2	5.3	3.01
Il1b	AGGGGGAAAT	39913	5.6	<4.0	5.6	2.00
Tnfsf9	GGGGAGGGAA	30385	4.8	<4.0	4.3	2.32
Nr4a1	GGGGACGGGG	25607	4.7	<4.0	<4.0	2.31
Gem	GGGGCTTTTC	45697	4.5	<4.0	<4.0	1.84
Ccl4	GGGGGAAGAC	11852	<4.0	<4.0	<4.0	2.35
Cybb	GGGGGAAGAC	68192	<4.0	<4.0	<4.0	1.82
Malt1	TGGGGAAATG	32989	<4.0	<4.0	4.8	1.93
Tnf	GGGGGAGAAC	29677	<4.0	<4.0	<4.0	1.83
C5ar1	GGGGAAAAGG	57777	<4.0	<4.0	<4.0	1.45
Plek	GGGGAAAAGG	09432	<4.0	<4.0	<4.0	2.04

n=25
Mean Rel/RelA ratio=2.00
Standard Deviation=0.3566

SORTED BY RelA:p50 MOTIF Z SCORES

p-value between Datasets 1 and 2 below: <0.001
Ratios 1.80 or less: Dataset 1, n=28, Dataset 2, n=3
Ratios 2.00 or greater: Dataset 1, n=4, Dataset 2, n=14
Ratios between 1.81 and 1.99: Dataset 1, n=8, Dataset 2, n=8

Dataset 1: Promoters with consensus motifs (motif score 6.4 or greater; n=40)

RelA: Rel: RelA: Rel/RelA

Gene	Motif	Peak#	p50 Z	Rel Z	RelA Z	RPKM Ratio
Tlr2	GGGGAATTCC	43113	12.2	14.8	11.6	1.72
Nkbib	GGGGAATTCC	58154	12.2	14.8	11.6	1.38
Sdc4	GGGGAATTCC	41035	12.2	14.8	11.6	1.52
Relb	GGGGAATTCC	57900	12.2	14.8	11.6	1.83
Nfkbid	GGGGAATTCC	58246	12.2	11.8	11.6	1.79
Bcl2l11	GGGAATTCCC	39793	11.5	17.7	13.2	1.39
Ebi3	GGGGGTTTCC	30307	10.6	11.2	8.2	1.79
Cxcl2	GGGAAATTCC	51430	10.3	18.2	13.9	1.69
Cxcl1	GGGAAATTCC	51426	10.3	18.2	13.9	1.68
Cxcl10	GGGAAATTCC	51487	10.3	18.2	13.9	1.97
Ifn1	GGGAAATTCC	47146	10.3	18.2	13.9	1.91
Tnfaip3	GGGGATTTCC	05530	10.0	11.4	9.2	1.71
Casp4	GGGGATTTCC	64595	10.0	11.4	9.2	1.78
Rel	GGGGATTTCC	09682	10.0	11.4	9.2	1.80
Nfkb1	GGGAATTTCC	44851	9.2	15.8	12.2	1.39
Nfkbia	GGGAATTTCC	14813	9.2	15.8	12.2	1.80
Nfkbiz	GGGAATTTCC	27524	9.2	15.8	12.2	1.46
Fchsd2	GGGAATTTCC	59885	9.2	15.8	12.2	1.64
Gbp5	GGGAATTTCC	45059	9.2	15.8	12.2	2.06
Ptgs2	GGGGATTCCC	03319	9.1	12.7	7.7	1.58
Nfkbie	GGGGGATTCC	29942	8.6	11.0	6.9	2.07
Nfkb2	GGGACTTTCC	35430	8.4	12.4	8.5	1.77
Csf1	GGGACTTTCC	44096	8.4	12.4	8.5	1.59
Stx11	GGGGTTTTCC	05245	8.4	10.9	7.1	1.94
Rapgef2	AGGGGGTTTT	43012	8.0	7.5	5.3	1.59
Ier3	TGGAATTTCC	29729	7.9	18.0	13.0	1.75
Acod1(Irg)	TGGAATTTCC	22405	7.9	18.0	13.0	1.56
Kdm6b	GGGGGATTTC	11036	7.4	6.6	5.4	1.59
Tnip3	GGGATTTCCC	55196	7.2	12.9	8.4	2.27
Cd40	GGGATTTCCC	41079	7.2	12.9	8.4	1.82
Icam1	TGGAAATTCC	64896	7.2	17.0	13.2	1.78
Traf1	AGGGGATTTT	37086	7.1	7.4	5.3	1.83
Irf1	GGGGAATCCC	10386	7.0	9.4	6.2	1.69
Ccl5	GGGAGTTTCC	11826	6.8	11.7	7.2	1.89
Gpr84	AGGGGGAATT	25702	6.8	5.9	5.9	1.58
Btg2	GGGAAAGTCC	02911	6.7	12.2	8.2	2.07
Ccrl2	GGGAAAGTCC	67639	6.7	12.2	8.2	1.76
Rela	AGGGGATTTC	33773	6.5	5.2	4.9	1.40
Cd83	GGGACTTCCC	17889	6.4	9.6	5.6	1.56
Bcl3	GGGACTTCCC	57917	6.4	9.6	5.6	1.83

n=40
Mean Rel/RelA ratio=1.73
Standard Deviation=0.2012

Dataset 2: Promoters with non-consensus motifs (motif scores <6.4, n=25)

Gene	Motif	Peak#	RelA: p50	Rel: Rel	RelA: RelA	Rel/RelA Ratio
Zhx2	AGGGGGAAAA	23954	6.1	4.5	6.1	2.07
Tfec	TGGGGAAAAA	53859	6.0	4.7	6.1	2.09
Cxcl16	CGGGGATTTC	11135	5.8	7.1	6.8	2.23
Dusp5	GGGTATTTCC	35632	5.7	8.2	5.5	2.24
Ier2	GGGTTTTCCC	63225	5.7	12.6	7.6	1.91
Cd44	GGGTTTTCCC	38999	5.7	12.6	7.6	1.89
Rasgef1b	GGGTTTTCCC	51638	5.7	12.6	7.6	1.87
Il1rn	TGGGGAAATT	36501	5.6	8.3	6.9	2.06
Il1b	AGGGGGAAAT	39913	5.6	<4.0	5.6	2.00
Slc2a6	GGGTTTCCCC	36646	5.2	8.9	5.8	2.60
Srgn	TGGGGAAAAC	06824	5.2	4.6	6.1	2.03
Ccl3	GGGAAAATTT	11848	4.9	6.8	7.0	1.77
Tnfsf9	GGGGAGGGAA	30385	4.8	<4.0	4.3	2.32
Nr4a1	GGGGACGGGG	25607	4.7	<4.0	<4.0	2.31
Cflar	TGGGGTTTTC	01044	4.6	7.9	4.8	2.01
Gm6377	GGGTGGGAAA	68997	4.5	4.2	5.3	3.01
Gem	GGGGCTTTTC	45697	4.5	<4.0	<4.0	1.84
Ccl4	GGGGGAAGAC	11852	<4.0	<4.0	<4.0	2.35
Cybb	GGGGGAAGAC	68192	<4.0	<4.0	<4.0	1.82

Slfn2	GGGATTCCCA	11801	<4.0	11.0	6.2	1.96
Malt1	TGGGGAAATG	32989	<4.0	<4.0	4.8	1.93
Tnf	GGGGGAGAAC	29677	<4.0	<4.0	<4.0	1.83
C5ar1	GGGGAAAAGG	57777	<4.0	<4.0	<4.0	1.45
Plek	GGGGAAAAGG	09432	<4.0	<4.0	<4.0	2.04
Clec4e	GGAAATTCTG	56755	<4.0	10.1	5.7	1.58

n=25
Mean Rel/RelA ratio=2.05
Standard Deviation=0.3126

<u>SORTED BY RelA:RelA MOTIF Z SCORES</u>

p-value between Datasets 1 and 2 below: 0.034
Ratios 1.80 or less: Dataset 1, n=23, Dataset 2, n=8
Ratios 2.00 or greater: Dataset 1, n=3, Dataset 2, n=15
Ratios between 1.81 and 1.99: Dataset 1, n=9, Dataset 2, n=7

Dataset 1: Promoters with RelA:RelA motif scores 7.0 or greater (n=35)

Gene	Motif	Peak#	RelA: p50	Rel: Rel	RelA: RelA	Rel/RelA Ratio
Cxcl2	GGGAAATTCC	51430	10.3	18.2	13.9	1.69
Cxcl1	GGGAAATTCC	51426	10.3	18.2	13.9	1.68
Cxcl10	GGGAAATTCC	51487	10.3	18.2	13.9	1.97
Ifn1	GGGAAATTCC	47146	10.3	18.2	13.9	1.91
Bcl2l11	GGGAATTCCC	39793	11.5	17.7	13.2	1.39
Icam1	TGGAAATTCC	64896	7.2	17.0	13.2	1.78
Ier3	TGGAATTTCC	29729	7.9	18.0	13.0	1.75
Acod1(Irg)	TGGAATTTCC	22405	7.9	18.0	13.0	1.56
Nfkb1	GGGAATTTCC	44851	9.2	15.8	12.2	1.39
Nfkbia	GGGAATTTCC	14813	9.2	15.8	12.2	1.80
Nfkbiz	GGGAATTTCC	27524	9.2	15.8	12.2	1.46
Fchsd2	GGGAATTTCC	59885	9.2	15.8	12.2	1.64
Gbp5	GGGAATTTCC	45059	9.2	15.8	12.2	2.06
Tlr2	GGGGAATTCC	43113	12.2	14.8	11.6	1.72
Nkbib	GGGGAATTCC	58154	12.2	14.8	11.6	1.38
Sdc4	GGGGAATTCC	41035	12.2	14.8	11.6	1.52
Relb	GGGGAATTCC	57900	12.2	14.8	11.6	1.83
Nfkbid	GGGGAATTCC	58246	12.2	11.8	11.6	1.79
Tnfaip3	GGGGATTTCC	05530	10.0	11.4	9.2	1.71
Casp4	GGGGATTTCC	64595	10.0	11.4	9.2	1.78
Rel	GGGGATTTCC	09682	10.0	11.4	9.2	1.80
Nfkb2	GGGACTTTCC	35430	8.4	12.4	8.5	1.77
Csf1	GGGACTTTCC	44096	8.4	12.4	8.5	1.59
Tnip3	GGGATTTCCC	55196	7.2	12.9	8.4	2.27
Cd40	GGGATTTCCC	41079	7.2	12.9	8.4	1.82
Btg2	GGGAAAGTCC	02911	6.7	12.2	8.2	2.07
Ccrl2	GGGAAAGTCC	67639	6.7	12.2	8.2	1.76
Ebi3	GGGGGTTTCC	30307	10.6	11.2	8.2	1.79
Ptgs2	GGGGATTCCC	03319	9.1	12.7	7.7	1.58
Ier2	GGGTTTTCCC	63225	5.7	12.6	7.6	1.91
Cd44	GGGTTTTCCC	38999	5.7	12.6	7.6	1.89
Rasgef1b	GGGTTTTCCC	51638	5.7	12.6	7.6	1.87
Ccl5	GGGAGTTTCC	11826	6.8	11.7	7.2	1.89
Stx11	GGGGTTTTCC	05245	8.4	10.9	7.1	1.94
Ccl3	GGGAAAATTT	11848	4.9	6.8	7.0	1.77

n=35
Mean Rel/RelA ratio=1.76
Standard Deviation=0.1946

Dataset 2: Promoters with RelA:RelA motif scores <7.0 (n=30)

Gene	Motif	Peak#	RelA: p50	Rel: Rel	RelA: RelA	Rel/RelA Ratio
Nfkbie	GGGGGATTCC	29942	8.6	11.0	6.9	2.07
Il1rn	TGGGGAAATT	36501	5.6	8.3	6.9	2.06

Cxcl16	CGGGGATTTC	11135	5.8	7.1	6.8	2.23
Slfn2	GGGATTCCCA	11801	<4.0	11.0	6.2	1.96
Irf1	GGGGAATCCC	10386	7.0	9.4	6.2	1.69
Tfec	TGGGGAAAAA	53859	6.0	4.7	6.1	2.09
Srgn	TGGGGAAAAC	06824	5.2	4.6	6.1	2.03
Zhx2	AGGGGGAAAA	23954	6.1	4.5	6.1	2.07
Gpr84	AGGGGGAATT	25702	6.8	5.9	5.9	1.58
Slc2a6	GGGTTTCCCC	36646	5.2	8.9	5.8	2.60
Clec4e	GGAAATTCTG	56755	<4.0	10.1	5.7	1.58
Cd83	GGGACTTCCC	17889	6.4	9.6	5.6	1.56
Bcl3	GGGACTTCCC	57917	6.4	9.6	5.6	1.83
Il1b	AGGGGGAAAT	39913	5.6	<4.0	5.6	2.00
Dusp5	GGGTATTTCC	35632	5.7	8.2	5.5	2.24
Kdm6b	GGGGGATTTC	11036	7.4	6.6	5.4	1.59
Rapgef2	AGGGGGTTTT	43012	8.0	7.5	5.3	1.59
Traf1	AGGGGATTTT	37086	7.1	7.4	5.3	1.83
Gm6377	GGGTGGGAAA	68997	4.5	4.2	5.3	3.01
Rela	AGGGGATTTC	33773	6.5	5.2	4.9	1.40
Cflar	TGGGGTTTTC	01044	4.6	7.9	4.8	2.01
Malt1	TGGGGAAATG	32989	<4.0	<4.0	4.8	1.93
Tnfsf9	GGGGAGGGAA	30385	4.8	<4.0	4.3	2.32
Nr4a1	GGGGACGGGG	25607	4.7	<4.0	<4.0	2.31
Gem	GGGGCTTTTC	45697	4.5	<4.0	<4.0	1.84
Ccl4	GGGGGAAGAC	11852	<4.0	<4.0	<4.0	2.35
Cybb	GGGGGAAGAC	68192	<4.0	<4.0	<4.0	1.82
Tnf	GGGGGAGAAC	29677	<4.0	<4.0	<4.0	1.83
C5ar1	GGGGAAAAGG	57777	<4.0	<4.0	<4.0	1.45
Plek	GGGGAAAAGG	09432	<4.0	<4.0	<4.0	2.04

n=30
Mean Rel/RelA ratio=1.93
Standard Deviation=0.3839

Supplementary Table 5. Properties of 20 strongest Rel-preferential, RelA-preferential, and p50-preferential promoters based on ChIP-seq RPKM ratios. This analysis lists relevant properties of the top 30 promoters (defined as -1500 to 500 relative to the TSS) exhibiting the greatest preferential ChIP-seq binding for Rel (Rel vs. RelA comparison), RelA (Rel vs. RelA comparison), and p50 (Rel vs. p50 comparison).

Top 30 of 1050 promoters (-1500 to +500) with Rel-preferential ChIP-seq peaks (Rel vs. RelA)

This list of Rel-preferential peaks from a Rel vs. RelA comparison shows the gene name, the ChIP-seq peak number, the Rel/RelA ChIP-seq RPKM ratio, the nascent transcript RNA-seq RPKM for the gene in unstimulated BMDMs, the maximum (Max) nascent transcript RNA-seq RPKM for the gene in a 6-h time course of BMDMs stimulated with lipid A (or, for the one gene that was repressed rather than activated upon lipid A stimulation, the minimum [Min] RPKM achieved in stimulated cells is shown in parenthesis), and the nascent transcript RNA-seq fold-induction for the gene from the unstimulated state to the maximum state in WT BMDMs. This table also shows the maximum nascent transcript RNA-seq RPKM achieved for each gene in *Rel*$^{-/-}$ BMDMs stimulated with lipid A, as well as the statistical significance (p-adj) of *Rel*-dependence. The three genes among the 30 that show the most significant *Rel*-dependence (*Tnfsf9, Il12b,* and *Noct*) are shown in bold. The five additional genes that show lesser *Rel*-dependence (*Plscr1, Tmem39a, Nudt17, Ccl12,* and *Ptafr*) are also in bold. Note that 27 of the 30 genes are expressed at >2 RPKM in stimulated cells and 25 of the 30 genes are induced >2-fold.

Gene	Peak#	Rel/RelA ChIP Ratio	WT Basal RNA (RPKM)	WT Max(Min) RNA (RPKM)	WT Fold Induction	***Rel***$^{-/-}$ **Max(Min) RNA (RPKM)**	***Rel*-Dependence p-adj**
9330179D12Rik	56903	4.04	Not found	–	–	–	–
Msr1	62170	3.70	3.64	13.24	3.6	23.73	0.361
2610001J05Rik	53804	3.62	1.45	3.32	2.3	2.55	0.891
Mir5619	51841	3.61	1.57	5.59	3.6	1.57	1.000
Skap2	54858	3.53	5.18	5.60	1.1	6.78	0.962
Ccl7	11750	3.38	5.35	62.77	11.7	88.27	0.954
Tab2	05074	3.26	3.75	8.42	2.2	7.31	0.991
***Tnfsf9* (–745)**	**30384**	**3.18**	**1.63**	**164.52**	**100.9**	**35.35**	**1.43E–08**
Plscr1	**67088**	**3.17**	**0.69**	**5.75**	**8.3**	**2.14**	**0.053**
Slc44a1	46406	3.09	0.59	9.42	16.0	5.99	0.538
Tmem39a	**27112**	**3.03**	**1.28**	**6.37**	**5.0**	**1.64**	**0.103**
Gm6377	68997	3.01	0.41	8.55	20.9	4.80	0.644
Nudt17	**43710**	**3.00**	**0.09**	**3.35**	**37.2**	**0.76**	**0.197**
Gprc5c	13171	2.97	0.07	0.42	Low Expr.	–	–
***Il12b* (–185)**	**10094**	**2.96**	**0.02**	**22.21**	**1110.5**	**1.03**	**2.90E–17**
Ccl12	**11759**	**2.90**	**0.65**	**8.39**	**12.9**	**6.66**	**0.071**
Samsn1	27792	2.87	0.59	5.18	8.8	5.15	0.481
Tet2	44788	2.82	2.59	12.25	4.7	14.01	0.540
Ptafr	**48436**	**2.78**	**5.67**	**13.57**	**2.4**	**11.01**	**0.001**
Il4ra	60632	2.77	3.95	40.97	10.4	47.88	0.741
Opn3	04307	2.77	0.34	0.21	Low Expr.	–	–
Mdm2	08578	2.76	12.83	53.20	4.1	69.66	0.562
6330407A03Rik	45506	2.73	8.50	19.27	2.3	23.70	0.939
Itgax	60819	2.72	0.28	0.40	Low Expr.	–	–
Nlrp3	10638	2.71	4.35	94.94	21.8	89.53	0.487
Arih2	67523	2.71	1.77	7.77	4.4	5.58	0.930
***Noct* (–650)**	**42410**	**2.69**	**1.70**	**72.09**	**42.4**	**6.48**	**4.33E–06**
Gsr	61956	2.69	1.24	3.77	3.0	5.39	0.783
Map3k5	05604	2.68	0.93	4.83	5.2	1.68	0.232
Stk38	29312	2.68	4.30	(2.00)	0.5	2.43	0.967
Tiparp	42797	2.67	1.89	25.48	13.5	12.19	0.835

Top 30 or 1050 promoters (-1500 to +500) with RelA-preferential ChIP-seq peaks (Rel vs. RelA)

This list of RelA-preferential peaks from a Rel vs. RelA comparison shows the gene name, the ChIP-seq peak number, the Rel/RelA ChIP-seq RPKM ratio, the nascent transcript RNA-seq RPKM for the gene in unstimulated BMDMs, the maximum (Max) nascent transcript RNA-seq RPKM for the gene in a 6-h time course of BMDMs stimulated with lipid A (or, for the seven genes that were repressed rather than activated upon lipid A stimulation, the minimum [Min] RPKM achieved in stimulated cells is shown in parenthesis), and the nascent transcript RNA-seq fold-induction for the gene from the unstimulated state to the maximum state in WT BMDMs (values not shown if the gene did not reach an expression level of 1 RPKM, as RPKM values below this threshold are often unreliable). Note that, in contrast to the findings above with Rel-preferential ChIP-seq peaks, only seven of these 30 genes are expressed at >1 RPKM in stimulated cells and only two are induced by lipid A >2-fold. These results suggest the possibility that RelA-preferential binding observed by ChIP-seq may not have functional relevance.

Gene	Peak#	Rel/RelA ChIP Ratio	WT Basal RNA (RPKM)	WT Max(Min) RNA (RPKM)	WT Fold Induction
Arntl	60292	0.68	0.61	0.25	Low Expr.
Camp	67562	0.84	0.04	0.02	Low Expr.
Csf3	12448	0.85	0.01	0.06	Low Expr.
Kbtbd7	22179	0.89	0.27	0.06	Low Expr.
Timm13	07433	0.93	1.19	0.56	Low Expr.
Sac3d1	33827	0.95	0.34	0.21	Low Expr.
Birc5	13411	0.99	3.28	(0.84)	0.3
Slc39a4	24667	1.00	0.04	0.05	Low Expr.
Blcap	40847	1.03	2.88	5.05	1.8
Smim10l1	57050	1.03	1.28	(0.93)	0.7
6430571L13Rik	67437	1.06	0.03	0.01	Low Expr.
Oxld1	13611	1.07	0.35	0.08	Low Expr.
Meig1	35890	1.08	0.00	0.01	Low Expr.
Gm7008	14534	1.08	0.02	0.04	Low Expr.
Lrch3	26842	1.09	1.56	2.83	1.8
Fpr1	28917	1.09	0.01	0.23	Low Expr.
Pih1d2	65672	1.11	0.04	0.05	Low Expr.
Ncor2	52751	1.12	2.79	(0.47)	0.2
Srd5a3	51198	1.13	0.91	0.42	Low Expr.
2410002F23Rik	58486	1.13	2.08	(0.78)	0.4
Peak1	65887	1.14	1.81	2.48	1.4
Ehf	39027	1.16	0.00	0.00	Low Expr.
Atg10	19008	1.17	0.67	0.26	Low Expr.
Nudt13	20240	1.17	0.90	0.29	Low Expr.
Zc3h12c	65734	1.19	0.76	21.23	27.9
Zc3hav1	54438	1.19	2.95	14.62	5.0
Mrpl17	60049	1.19	0.52	0.32	Low Expr.
E130307A14Rik	06120	1.20	0.49	0.22	Low Expr.
Atg16l2	59899	1.20	2.53	(1.06)	0.4
Rmi1	18453	1.21	1.41	(0.49)	0.3
Pde6d	01871	1.21	1.45	(0.68)	0.5
Mir688	25669	1.21	0.50	0.38	Low Expr.
Raver1	64906	1.21	3.00	4.52	1.5

Top 30 of 651 promoters (-1500 to +50) with p50-preferential ChIP-seq peaks (Rel vs. p50)

This list of p50-preferential peaks from a Rel vs. p50 comparison shows the gene name, the ChIP-seq peak number, the Rel/p50 ChIP-seq RPKM ratio, the nascent transcript RNA-seq RPKM for the gene in unstimulated BMDMs, the maximum (Max) nascent transcript RNA-seq RPKM for the gene in a 6-h time course of BMDMs stimulated with lipid A (or, for the twelve genes that were repressed rather than activated upon lipid A stimulation, the minimum [Min] RPKM achieved in stimulated cells is shown in parenthesis), and the nascent transcript RNA-seq fold-induction for the gene from the unstimulated state to the maximum state in WT BMDMs (values not shown if the gene did not reach an expression level of 1 RPKM, as RPKM values below this threshold are often unreliable). The table also shows the maximum (Max)or minimum for repressed genes (Min, in parenthesis) nascent transcript RNA-seq RPKM for the gene in *Nfkb1*$^{-/-}$ BMDMs stimulated with lipid A,[39] as well as the motif underlying the ChIP-seq peak that exhibits the highest PBM Z score for p50 homodimer binding, revealing that these promoters typically contain a motif with three G:C bps in each half-site separated by five or six intervening sequences. Note that only half of these 30 genes reach an RPKM expression level >1 and only two were induced >2-fold. Moreover, although twelve of the genes were repressed upon lipid A stimulation, similar repression was observed in *Nfkb1*$^{-/-}$ BMDMs, demonstrating that the repression was not *Nfkb1*-dependent for any of the twelve genes. Data from control genes that are known to be strongly induced by lipid A and that exhibit either *Nfkb1*-dependent induction or *Nfkb1*-independent induction are shown at the bottom.

Gene	Peak#	Rel/p50 ChIP Ratio	WT Basal RNA (RPKM)	WT Max(Min) RNA (RPKM)	WT Fold Induction	*Nfkb1*$^{-/-}$ Max(Min) RNA (RPKM)	Motif
H2-Aa	29577	0.14	0.31	1.06	3.3	1.52	AGGGGAACTCCC
2810414N06Rik	03406	0.19	0.06	0.01	Low Expr.	0.01	GGGACAGCTCCC
Akr1b8	54328	0.29	0.60	0.07	Low Expr.	0.04	GGGAAATCTCCC
Mrpl48	59834	0.30	0.36	0.15	Low Expr.	0.19	GGGGACTCTCCC
Zbtb3	33942	0.38	0.20	0.42	Low Expr.	0.40	GGGTAATTTCCC
Stard4	32005	0.38	1.52	2.83	1.9	1.79	AGGGACATTCCC
Myadm	57563	0.39	2.03	5.81	2.9	6.76	AGGGAAAGTCCC
Slc29a2	33733	0.39	0.18	0.02	Low Expr.	0.03	GGGGGAACTCCC
Rbm39	40753	0.41	11.25	18.00	1.6	15.85	AGGGAGACTCCC
Unc119	11552	0.42	1.58	(0.02)	0.1	(0.13)	GGGGAGCGTCCG
Immp2l	14574	0.42	0.14	0.02	Low Expr.	0.02	GGGGACTTCCCC
Barhl1	36737	0.42	0.03	0.01	Low Expr.	0.01	AGGGAAAGTCCC
Hirip3	60722	0.43	2.39	(0.11)	0.0	(0.13)	GGGGGAAGACCC
Golga7	61769	0.44	1.42	(0.61)	0.4	(0.61)	GGGGAGAGTCCC
Tmem206	04809	0.46	4.71	(0.42)	0.1	(0.49)	GGGGAAAGCACC
Dap3	43372	0.48	1.08	(0.79)	0.7	(0.77)	GGGGAAGTTCCC
Plgrkt	34689	0.52	2.75	(1.39)	0.5	(1.44)	GGGGCTCTGCCG
Baz1b	52926	0.53	4.04	(1.18)	0.3	(1.28)	AGGGAATCTCCC
Pafah1b3	58019	0.53	0.60	0.10	Low Expr.	0.07	GGGGACTTCCCT
Sec23a	14852	0.54	0.43	0.09	Low Expr.	0.11	AGGGGCGGCCCC
Pan3	53478	0.55	4.14	(2.67)	0.6	(2.98)	AGGGAGAATCCC
Cd70	30389	0.56	0.01	0.07	Low Expr.	0.02	AGGGAAATCCCC
Gm11627	12675	0.56	0.02	0.02	Low Expr.	0.02	GGGGAAAACCCT
Ubp1	67686	0.57	3.02	(2.11)	0.7	(2.00)	AGGGTATTTCCC
Ptgr2	15606	0.57	0.97	0.07	Low Expr.	0.10	AGGGAATGCCCC
Abhd11	52914	0.59	1.32	(0.34)	0.3	(0.45)	GGGGACTTGCCC
Ilk	60039	0.59	2.81	(1.45)	0.5	(1.71)	CGGGAGTTCCCC
Nr2f6	62817	0.59	1.22	(0.22)	0.2	(0.16)	GGGAAAAGTCCC
Plk4	42276	0.60	1.04	1.37	1.3	1.29	AGGGAAAGCCCC
Gm15972	31816	0.61	0.18	0.04	Low Expr.	0.07	GGGGCTCTCCCG
Foxd2	47718	0.61	0.05	0.02	Low Expr.	0.03	GGGGATGTCCCC
Rida	23434	0.61	0.26	0.01	Low Expr.	0.02	AGGGACAGCCCC

Controls for induction and p50 dependence (Il6 and Il4i1) and independence (Nfkbia):

Il6 (p50–dependent)	0.01	6.84	684.0	1.82
Il4i1 (p50–dependent)	0.36	10.48	29.1	1.34
Nfkbia (p50–independent)	3.36	120.89	36.0	110.68

Supplementary Table 6. Crystallographic data collection and refinement statistics.

PDB ID	**8U9L**
Data collection	
Space group	*P*1
Cell dimensions	
a, b, c (Å)	65.91 110.464 162.836
α, β, γ (°)	82.202 77.983 72.809
Resolution (Å)	47.92-3.10(3.2-3.1)*
R_{merge}	0.08848 (0.5165)*
CC1/2	0.993 (0.819)*
$I/\sigma I$	8.7 (1.7)*
Completeness	98.2 (98.2)*
Redundancy	1.9 (1.9)
Refinement	
**Resolution (Å)**	47.92-3.10
R_{work}/R_{free}	0.2768/0.3288
**No. of atoms**	
Macromolecules	20864
**B-factor**	
Macromolecules	85.58
R. m. s. deviations	
Bond lengths (Å)	0.011
Bond angles (°)	1.35

*Highest-resolution shell is shown in parentheses.
