# Supplementary material for: Stepwise neofunctionalization of the NF-κB family member Rel during vertebrate evolution
Source: Nat Immunol. 2025 Apr 30;26(5):760–74. doi: 10.1038/s41590-025-02138-2 (PMC12043515; doi:10.1038/s41590-025-02138-2)
Supplement: Supplementary file 2 — Reporting Summary [file 41590_2025_2138_MOESM2_ESM.pdf]

nature portfolio

Corresponding author(s): Stephen T. Smale

Last updated by author(s): Mar 15, 2025

Reporting Summary

Nature Portfolio wishes to improve the reproducibility of the work that we publish. This form provides structure for consistency and transparency in reporting. For further information on Nature Portfolio policies, see our Editorial Policies and the Editorial Policy Checklist.

Statistics

For all statistical analyses, confirm that the following items are present in the figure legend, table legend, main text, or Methods section.

n/a	Confirmed	
☐	☒	The exact sample size (*n*) for each experimental group/condition, given as a discrete number and unit of measurement
☐	☒	A statement on whether measurements were taken from distinct samples or whether the same sample was measured repeatedly
☐	☒	The statistical test(s) used AND whether they are one- or two-sided<br>*Only common tests should be described solely by name; describe more complex techniques in the Methods section.*
☒	☐	A description of all covariates tested
☐	☒	A description of any assumptions or corrections, such as tests of normality and adjustment for multiple comparisons
☐	☒	A full description of the statistical parameters including central tendency (e.g. means) or other basic estimates (e.g. regression coefficient) AND variation (e.g. standard deviation) or associated estimates of uncertainty (e.g. confidence intervals)
☒	☐	For null hypothesis testing, the test statistic (e.g. *F*, *t*, *r*) with confidence intervals, effect sizes, degrees of freedom and *P* value noted<br>*Give P values as exact values whenever suitable.*
☒	☐	For Bayesian analysis, information on the choice of priors and Markov chain Monte Carlo settings
☒	☐	For hierarchical and complex designs, identification of the appropriate level for tests and full reporting of outcomes
☒	☐	Estimates of effect sizes (e.g. Cohen's *d*, Pearson's *r*), indicating how they were calculated

Our web collection on statistics for biologists contains articles on many of the points above.

Software and code

Policy information about availability of computer code

Data collection	Sequencing data was collected on a Hiseq 2000 (chromatin associated RNA-seq) or Hiseq 3000 (ChIP-seq).
Data analysis	Software used for the processing and analysis of the ChIP-seq and RNA-seq data is described in the methods section. ChIP-seq and RNA-seq FASTQ files were aligned to the mm9 genome (Ensembl v67) using Hisat2 V2.1.1. Following alignment, SAM files were compressed and sorted using SAMTools V1.19.2. ChIP-seq peaks were called with HOMER V4.11 under the factor mode of operation, compared to a paired input control, with a false discovery rate < 0.01. To generate a complete set of peaks, BEDTools V2.31.0 was used to merge any peaks with direct overlap of > 1bp. For both ChIP-seq and RNA-seq, SeqMonk V1.48.0 was used to extract raw read counts from BAM files. To generate RPKMs, raw counts were divided by the sequencing depth of the sample (million reads) and the size of the gene or peak (kbps), for RNA-seq and ChIP-seq, respectively. For motif analysis, HOMER V4.11 searched for both known and de novo motifs +/- 200bps from the center of the ChIP-seq peaks. To annotate ChIP-seq peaks to genes, HOMER V4.11 was used, which defines promoter peaks as +/- 1000bps from the TSS. For statistical analysis, DESeq2 was used to generate adjusted p-values.

For manuscripts utilizing custom algorithms or software that are central to the research but not yet described in published literature, software must be made available to editors and reviewers. We strongly encourage code deposition in a community repository (e.g. GitHub). See the Nature Portfolio guidelines for submitting code & software for further information.

Data

Policy information about availability of data

All manuscripts must include a data availability statement. This statement should provide the following information, where applicable:

- Accession codes, unique identifiers, or web links for publicly available datasets
- A description of any restrictions on data availability
- For clinical datasets or third party data, please ensure that the statement adheres to our policy

All sequencing data in this manuscript of publicly available on NCBI's Gene Expression Omnibus (SuperSeries GSE243012). RNA-seq data is available at the accession number GSE243011(https://www.ncbi.nlm.nih.gov/geo/query/acc.cgi?acc=GSE243011). ChIP-seq data is available at the accession number GSE243010 (https://www.ncbi.nlm.nih.gov/geo/query/acc.cgi?acc=GSE243010). Two of the RelA ChIP-seq replicates with lipid-A stimulation for 1.0h are available with the accession number GSE67357 (https://www.ncbi.nlm.nih.gov/geo/query/acc.cgi?acc=GSE67357). The X-ray crystallographic data are available at the Protein Data Bank, PDB ID: 8U9L.

Research involving human participants, their data, or biological material

Policy information about studies with human participants or human data. See also policy information about sex, gender (identity/presentation), and sexual orientation and race, ethnicity and racism.

Reporting on sex and gender	N/A
Reporting on race, ethnicity, or other socially relevant groupings	N/A
Population characteristics	N/A
Recruitment	N/A
Ethics oversight	N/A

Note that full information on the approval of the study protocol must also be provided in the manuscript.

Field-specific reporting

Please select the one below that is the best fit for your research. If you are not sure, read the appropriate sections before making your selection.

☒ Life sciences ☐ Behavioural & social sciences ☐ Ecological, evolutionary & environmental sciences

For a reference copy of the document with all sections, see nature.com/documents/nr-reporting-summary-flat.pdf

Life sciences study design

All studies must disclose on these points even when the disclosure is negative.

Sample size	RNA-seq for wild-type and Rel-/- BMDMs stimulated with lipid-A for 0 h, 1 h, and 2 h were generated with two biological replicates. Our interest was in genes that are induced and expressed at high levels and show a strong dependence on Rel (i.e. we did not examine large sets of genes that show small but statistically significant differential expression). Since this was our focus, two replicates for RNA-seq analysis were sufficient to capture statistically significant differences in samples. ChIP-seq for Rel and p50 were generated in biological triplicate with lipid-A stimulation for 1 h. ChIP-seq for RelA was generated with five biological replicates with lipid-A stimulation for 1 h. The number of ChIP-seq replicates was found to be appropriate for reproducibility and significance based on the PCA-based analysis strategy described below (Data exclusion and Replication sections).
Data exclusions	Because ChIP-seq results depend heavily on the amount of protein:protein crosslinker (DSG) and protein:DNA (PFA) used, we performed full ChIP-seq experiments titrating both crosslinkers to determine the optimal conditions for Rel, RelA, and p50 ChIP-seq (Daly et al., in preparation). Given this optimization, we determined the optimal crosslinking conditions of 1.0mM DSG and 1% PFA for NF-kB ChIP-seq. After performing this optimization, we had obtained 4-replicates for Rel, and p50, and 5 biological replicates for RelA at 1.0mM DSG and 1% PFA. To keep the downstream analysis consistent for different members of NF-kB, we performed PCA analysis for all replicates and choose the three most reproducible samples for each p50, Rel, RelA. Our lab had previously generated RelA ChIP-seq in biological replicates. Since these data-set clustered with our new RelA ChIP-seq by PCA, we decided to include these two additional RelA ChIP-seq datasets in the downstream analysis.
Replication	ChIP-seq was carried out with three biological replicates for Rel and p50. Peaks were considered reproducible if their peak score >19 and RPKM >3 in 2/3 replicates. RelA ChIP-seq was carried out with five biological replicates. Peaks were considered reproducible if their peak score >19 and RPKM >3 in 3/5 replicates. RNA-seq was carried out with two biological replicates. For RNA-seq replicates, RPKM values were averaged. Only genes with a wild-type expression at any time-point (0-6 h lipid-A stimulation) with an RPKM >3 were considered in the downstream analyses. As indicated in the Data exclusion section, the analyses were restricted to the datasets generated with crosslinking conditions that were found to be the most optimal, and with datasets found by PCA to be the most consistent. Further evidence of the validity

of this approach was provided by the analysis shown in Supplementary Table 4 and Fig. 4f, which objectively evaluated RPKM ratios from the perspective of previously defined ChIP-seq peaks and their corresponding Protein Binding Microarray z scores.

Randomization

For ChIP-seq and RNA-seq, bone marrow was extracted from male mice aged 10-12 weeks old and differentiated ex vivo into BMDMs. For each experiment the bone marrow from 2-4 randomly selected mice was pooled together. This represents the greatest extent to which randomization can be achieved with the genomic experiments and other experiments described in this study.

Blinding

The high-throughout sequencing performed for the RNA-seq and ChIP-seq experiments was blinded to the extent that the core facility staff had no knowledge of the sample labels or their significance. Protocols for RNA-seq and ChIP-seq preparation are well-defined and we did not anticipate any biases that would affect the outcomes. Since the analysis of gene expression and ChIP-seq signal typically relies on large-scale data processing and computational methods, blinding was not deemed necessary for the statistical analysis or data interpretation. Blinding of non-genomic experiments would have been difficult to achieve due to the nature of the molecular biology and structural experiments.

Reporting for specific materials, systems and methods

We require information from authors about some types of materials, experimental systems and methods used in many studies. Here, indicate whether each material, system or method listed is relevant to your study. If you are not sure if a list item applies to your research, read the appropriate section before selecting a response.

Materials & experimental systems

n/a	Involved in the study
☐	☒ Antibodies
☐	☒ Eukaryotic cell lines
☒	☐ Palaeontology and archaeology
☐	☒ Animals and other organisms
☒	☐ Clinical data
☒	☐ Dual use research of concern
☒	☐ Plants

Methods

n/a	Involved in the study
☐	☒ ChIP-seq
☒	☐ Flow cytometry
☒	☐ MRI-based neuroimaging

Antibodies

Antibodies used

Anti-Rel (67489- D3B8S, Cell Signaling Technologies); Anti-p50 (13586- D4P4D, Cell Signaling Technologies); Anti-RelA (8242S-D14E12, Cell Signaling Technologies). For ChIP-seq all antibodies were diluted 1:1000 during the immunoprecipitation.

Validation

The specificity of the Rel and p50 antibodies were validated in our lab through ChIP-seq experiments performed with BMDMs from mutant mice. In ChIP-seq assays with anti-Rel antibody (67489- D3B8S, Cell Signaling Technologies) performed in Rel-/- BMDMs, fewer than 100 total peaks were detected at any timepoint with lipid A stimulation from 0-6 h. In ChIP-seq assays with anti-p50 antibody (13586- D4P4D, Cell Signaling Technologies) performed in Nfkb1-/- BMDMs stimulated with lipid A for 1h, fewer than 100 total peaks were detected. The Cell Signaling Technologies webpage shows validation of the RelA antibody in the form of immunoblots and ChIP-seq peaks at desired locations and they state that the RelA antibody does not cross react with other NF-kB family members (https://www.cellsignal.com/products/primary-antibodies/nf-kb-p65-d14e12-xp-rabbit-mab/8242?srsltid=AfmBOoqUYnq1k4Fj2o_1LRlJSPpoF8czYVlr3XqMSMZNffXCq6drhatF).

Eukaryotic cell lines

Policy information about cell lines and Sex and Gender in Research

Cell line source(s)

HEK293T cells were obtained from ATCC (CRL-3216). The mouse R1 ESC line was from ATCC (SCRC-1011).

Authentication

Cell lines were not authenticated. The biological properties of the HEK293T cells were only relevant to the extent that they allows overexpression of proteins from transfected plasmids. Macrophages generated from the ESC line were authenticated on the basis of flow cytometry profiles and gene expression of macrophage markers.

Mycoplasma contamination

Cell lines were not routinely tested for mycoplasma contamination.

Commonly misidentified lines
(See ICLAC register)

Name any commonly misidentified cell lines used in the study and provide a rationale for their use.

Animals and other research organisms

Policy information about studies involving animals; ARRIVE guidelines recommended for reporting animal research, and Sex and Gender in Research

Laboratory animals

BMDMs were prepared from 8-10-week old male C57BL/6 mice and Rel-/- mice on a C57BL/6 background. Mice were maintained by the UCLA Division of Laboratory Animal Medicine under standard housing conditions. Animals were used only for the preparation of BMDMs.

Wild animals | No wild animals were used in the study.

Reporting on sex | RNA-seq and ChIP-seq data were collected only with BMDMs from male mice. Given the nature of these mechanistic experiments and their cost, sex differences were not evaluated.

Field-collected samples | No field-collected samples were used in the study.

Ethics oversight | These experiments were performed following approval by the UCLA Chancellor's Animal Research Committee (Protocol #1999-073) in accordance with all federal, state, and local guidelines.

Note that full information on the approval of the study protocol must also be provided in the manuscript.

Plants

Seed stocks | N/A

Novel plant genotypes | N/A

Authentication | N/A

ChIP-seq

Data deposition

☒ Confirm that both raw and final processed data have been deposited in a public database such as GEO.

☒ Confirm that you have deposited or provided access to graph files (e.g. BED files) for the called peaks.

Data access links
May remain private before publication.
The GEO accession number for three biological replicates of Rel, p50, and RelA ChIP-seq is GSE243010. (https://www.ncbi.nlm.nih.gov/geo/query/acc.cgi?acc=GSE243010). Two of the RelA ChIP-seq replicates are accessible at GSE67357 (https://www.ncbi.nlm.nih.gov/geo/query/acc.cgi?acc=GSE67357).

Files in database submission
For the GEO accession number GSE243010: cRel_1.0h_R1.fastq.gz, cRel_1.0h_R2.fastq.gz, cRel_1.0h_R3.fastq.gz, p50_1.0h_R1.fastq.gz, p50_1.0h_R2.fastq.gz, p50_1.0h_R3.fastq.gz, RelA_1.0h_R1.fastq.gz, RelA_1.0h_R2.fastq.gz, RelA_1.0h_R3.fastq.gz, Input_RelA_R3.fastq.gz, Input_cRel_R1.fastq.gz, Input_cRel_R3.fastq.gz, Input_cRel_RelA_R2.fastq.gz, Input_p50_R3.fastq.gz, Input_p50_RelA_R1.fastq.gz, cRel_1.0h_R1.bedGraph.gz, cRel_1.0h_R2.bedGraph.gz, cRel_1.0h_R3.bedGraph.gz, p50_1.0h_R1.bedGraph.gz, p50_1.0h_R2.bedGraph.gz, p50_1.0h_R3.bedGraph.gz, RelA_1.0h_R1.bedGraph.gz, RelA_1.0h_R2.bedGraph.gz, RelA_1.0h_R3.bedGraph.gz, Input_RelA_R3.bedGraph.gz, Input_cRel_R1.bedGraph.gz, Input_cRel_R3.bedGraph.gz, Input_cRel_RelA_R2.bedGraph.gz, Input_p50_R3.bedGraph.gz, Input_p50_RelA_R1.bedGraph.gz. For the GEO accession number GSE67357: RELA-60.fastq.gz, RELA-60-Rep2.fastq.gz, RELA-60.bedGraph.gz, RELA-60-Rep2.bedGraph.gz

Genome browser session
(e.g. UCSC)
N/A

Methodology

Replicates | There are three biological replicates for p50 and Rel ChIP-seq samples. There are five biological replicates for RelA ChIP-seq.

Sequencing depth | RelA ChIP-seq read depths range from 15 million – 26 million reads per sample. Rel ChIP-seq read depth ranges from 24 million – 30 million reads per sample. p50 ChIP-seq read depth ranges from 16 – 30 million reads per sample.

Antibodies | Anti-Rel (67489- D3B8S, Cell Signaling Technologies); Anti-p50 (13586- D4P4D, Cell Signaling Technologies); Anti-RelA (8242S-D14E12, Cell Signaling Technologies).

Peak calling parameters | HOMER software (http://homer.ucsd.edu/homer/ngs/peaks.html) was used to call ChIP-seq peaks. Inputs samples performed in parallel with each sample were used as the background signal during peak calling. Peaks were called with a false discovery rate (FDR) of < 0.01. Any peaks with direct overlap of > 1bp were merged for downstream analysis.

Data quality | The quality of the ChIP-seq data for p50, RelA, and Rel was checked in a variety of ways. Principle component analysis was performed to compare the overall similarity between samples and biological replicates; total peak number and the distribution of peak score and RPKM was evaluated. Rel R1 peak number = 20,338; Rel R2 peak number = 23,816; Rel R3 peak number = 20,278; RelA R1 peak number = 3,203; RelA R2 peak number = 10,050; RelA R3 peak number = 5,759; RelA R4 peak number = 10,245; RelA R5 peak number = 10,157; p50 R1 peak number = 3,563; p50 R2 peak number = 3,690; p50 R3 peak number = 2,478.

Software

Hisat2 V2.1.1 was used to map demultiplexed FASTQ files to the mm9 genome (Ensembl v67). SAMTools V1.19.2 was used to compress and sort files. HOMER software was used to further sorted using makeTagDirectory.pl, while keeping all unique reads. ChIP-seq peaks were called with HOMER V4.11 with the command findPeaks.pl with the factor mode of operation, compared to a paired input control, with a false discovery rate < 0.01. BEDTools V2.31.0 was used to merge any peaks with direct overlap of > 1bp. SeqMonk V1.48.0 was used to extract raw read counts from BAM files. HOMER V4.11 searched for both known and de novo motifs +/- 200bps from the center of the ChIP-seq peaks with the commands findMotifsGenome.pl. To annotate ChIP-seq peaks to genes, HOMER V4.11 was used, with the command annotatePeaks.pl to annotate promoter, exonic, intronic, and intergenic regions.
